# The microRNA expression signature of pancreatic ductal adenocarcinoma by RNA sequencing: anti-tumour functions of the *microRNA-216* cluster

**DOI:** 10.18632/oncotarget.19591

**Published:** 2017-07-26

**Authors:** Keiichi Yonemori, Naohiko Seki, Tetsuya Idichi, Hiroshi Kurahara, Yusaku Osako, Keiichi Koshizuka, Takayuki Arai, Atsushi Okato, Yoshiaki Kita, Takaaki Arigami, Yuko Mataki, Yuko Kijima, Kosei Maemura, Shoji Natsugoe

**Affiliations:** ^1^ Department of Digestive Surgery, Breast and Thyroid Surgery, Graduate School of Medical Sciences, Kagoshima University, Sakuragaoka, Kagoshima 890-8520, Japan; ^2^ Department of Functional Genomics, Chiba University Graduate School of Medicine, Chuo-ku, Chiba 260-8670, Japan

**Keywords:** pancreatic ductal adenocarcinoma, microRNA, expression signature, *miR-216b-3p*, FOXQ1

## Abstract

We analysed the RNA sequence-based microRNA (miRNA) signature of pancreatic ductal adenocarcinoma (PDAC). Aberrantly expressed miRNAs were successfully identified in this signature. Using the PDAC signature, we focused on 4 clustered miRNAs, *miR-216a-5p*, *miR-216a-3p*, *miR-216b-5p* and *miR-216b-3p* on human chromosome 2p16.1. All members of the *miR-216* cluster were significantly reduced in PDAC specimens. Ectopic expression of these miRNAs suppressed cancer cell aggressiveness, suggesting *miR-216* cluster as anti-tumour miRNAs in PDAC cells. The impact of *miR-216b-3p* (passenger strand of pre-*miR-216b*) on cancer cells is still ambiguous. Forkhead box Q1 (*FOXQ1*) was directly regulated by *miR-216b-3p* and overexpression of *FOXQ1* was confirmed in clinical specimens. High expression of *FOXQ1* predicted a shorter survival of patients with PDAC by Kaplan–Meier analysis. Loss-of-function assays showed that cancer cell migration and invasion activities were significantly reduced by si*FOXQ1* transfectants. We investigated pathways downstream from *FOXQ1* by using genome-wide gene expression analysis. Identification of the *miR-216-3p/FOXQ1*-mediated network in PDAC should enhance understanding of PDAC aggressiveness at the molecular level.

## INTRODUCTION

Pancreatic ductal adenocarcinoma (PDAC) is a highly lethal malignancy, as the 5-year survival rate after diagnosis is only 5% [1, 2]. PDAC cells are extremely aggressive, and more than 50% of patients develop local recurrence or distant metastasis after curative resection [3, 4]. Recently developed molecularly targeted therapeutics have not shown beneficial effects in patients with PDAC [5, 6]. The molecular pathogenesis of the aggressive phenotype in PDAC remains unclear. Therefore, in order to improve disease outcomes in PDAC patients, it is necessary to define the molecular pathogenesis underlying the aggressiveness of PDAC using advanced genomic approaches.

microRNAs (miRNAs) belong to a family of short, non-coding RNAs transcribed from the human genome. Their discovery suggested new directions for the study of human cancer pathogenesis [7]. A unique characteristic of miRNAs is the ability of a single miRNA to tune a large number of RNA transcripts in human cells [8]. Thus, dysregulated miRNA expression can disrupt tightly controlled RNA networks and increase the aggressiveness of cancer cells. Numerous studies have indicated that miRNAs are aberrantly expressed in several cancers, including PDAC [9–12].

We have been identified anti-tumour miRNAs and their mediated novel cancer networks based on the miRNA expression signatures in various cancers [13–20]. Construction of miRNA signatures using clinical specimens is a first step towards identification of dysregulated miRNAs and their resultant clinical characteristics. Current RNA sequencing methods offer superior analytical approaches for studying the transcriptome. We recently showed that the passenger strand of *miR-145-3p* acted as an anti-tumour miRNA through regulating of several oncogenic genes in bladder cancer and lung cancer [21, 22]. Moreover, we showed that both strands of *pre*-*miR-139* (*miR-139-5p* and *miR-139-3p*) targeted matrix metalloprotease 11 in bladder cancer [23]. The involvement of passenger strand miRNAs in the regulation of cellular processes is a novel concept in RNA research.

In this study, we analysed the miRNA expression signature of PDAC clinical specimens by RNA sequencing. Using the PDAC signature, we focused on 4 clustered miRNAs, *miR-216a-5p*, *miR-216a-3p*, *miR-216b-5p* and *miR-216b-3p* on human chromosome 2p16.1 and investigated their functional significance in PDAC pathogenesis. Transcription of all members of the *miR-216* cluster was significantly reduced in PDAC specimens, and ectopic expression of these miRNAs suppressed cancer cell aggressiveness. We further investigated the target genes of *miR-216*. The resultant data provide novel insights into the molecular pathogenesis of PDAC.

## RESULTS

### Construction of expression signature and identification of downregulated miRNAs in PDAC

Libraries of small RNAs (from seven PDAC tissues and four normal pancreatic tissues) were analysed by using RNA sequencing technology. The patients’ characteristics are shown in Table 1. Initially, 16,930,437 to 24,733,299 raw sequence reads were produced for the libraries. After filtering for low quality and adaptors sequence reads, from 10,615,842 to 23,407,926 clean small RNA reads were obtained (Table 2). Differentially expressed miRNAs by RNA sequence were summarized in Supplementary Tables 1 and 2.

**Table 1:** Clinical features of PDAC patients

No.	Age	Sex	T	N	M	Stage	Recurrence	Remarks
T1	44	M	3	1	0	IIB	(+)	deep sequencing
T2	76	M	4	1	0	III	(+)	deep sequencing
T3	67	M	3	1	0	IIB	(+)	deep sequencing
T4	65	M	3	1	0	IIB	(+)	deep sequencing
T5	56	F	3	1	1	IV	(+)	deep sequencing
T6	83	F	3	0	0	IIA	(-)	deep sequencing
T7	66	F	3	1	0	IIB	(-)	deep sequencing
N1	77	F						deep sequencing
N2	65	F						deep sequencing
N3	71	F						deep sequencing
N4	83	F						deep sequencing
PCR_T1	67	M	3	1	0	IIB	(+)	RT-PCR
PCR_T2	78	F	3	0	0	IIA	(-)	RT-PCR
PCR_T3	58	F	3	0	0	IIA	(+)	RT-PCR
PCR_T4	42	F	3	1	0	IIB	(+)	RT-PCR
PCR_T5	56	F	3	0	0	IIA	(+)	RT-PCR
PCR_T6	79	F	3	0	0	IIA	(+)	RT-PCR
PCR_T7	63	F	3	0	0	IIA	(+)	RT-PCR
PCR_T8	52	M	3	0	0	IIA	(-)	RT-PCR
PCR_T9	78	F	3	1	0	IIB	(+)	RT-PCR
PCR_T10	66	F	3	1	0	IIB	(-)	RT-PCR
PCR_T11	66	F	3	0	0	IIA	(-)	RT-PCR
PCR_T12	67	M	is	0	0	0	(+)	RT-PCR
PCR_T13	74	M	4	0	0	III	(-)	RT-PCR
PCR_T14	74	F	3	1	0	IIB	(-)	RT-PCR
PCR_T15	65	F	3	1	0	IIB	(-)	RT-PCR
PCR_T16	78	F	3	0	0	IIA	(-)	RT-PCR
PCR_T17	42	M	4	1	0	III	(+)	RT-PCR
PCR_T18	44	M	3	1	0	IIB	(+)	RT-PCR
PCR_T19	76	M	3	1	0	IIB	(+)	RT-PCR
PCR_T20	70	M	3	0	0	IIA	(+)	RT-PCR
PCR_T21	78	F	1	0	0	I	(-)	RT-PCR
PCR_T22	56	M	1	0	0	I	(+)	RT-PCR
PCR_T23	65	M	3	0	0	IIA	(-)	RT-PCR
PCR_T24	70	F	3	1	0	IIB	(+)	RT-PCR
PCR_N1	76	M						RT-PCR
PCR_N2	58	F						RT-PCR
PCR_N3	67	M						RT-PCR
PCR_N4	67	M						RT-PCR
PCR_N5	42	F						RT-PCR
PCR_N6	71	F						RT-PCR
PCR_N7	60	M						RT-PCR
PCR_N8	56	F						RT-PCR
PCR_N9	67	M						RT-PCR
PCR_N10	85	F						RT-PCR
PCR_N11	66	F						RT-PCR
PCR_N12	66	F						RT-PCR
PCR_N13	44	M						RT-PCR
PCR_N14	63	F						RT-PCR
IHC_1	72	M	3	1	0	IIB	(+)	IHC
IHC_2	80	M	3	1	0	IIB	(-)	IHC
IHC_3	75	F	3	1	0	IIB	(-)	IHC
IHC_4	60	M	4	0	0	III	(+)	IHC
IHC_5	52	M	3	0	0	IIA	(+)	IHC
IHC_6	50	M	4	0	0	III	(+)	IHC
IHC_7	68	F	4	0	0	III	(-)	IHC
IHC_8	77	F	4	0	0	III	(+)	IHC
IHC_9	57	M	3	1	0	IIB	(+)	IHC
IHC_10	44	M	3	1	0	IIB	(+)	IHC
IHC_11	68	M	4	1	0	III	(+)	IHC
IHC_12	76	M	4	1	0	III	(+)	IHC
IHC_13	54	M	3	1	0	IIB	(+)	IHC
IHC_14	74	F	3	1	0	IIB	(+)	IHC
IHC_15	67	M	3	1	0	IIB	(+)	IHC
IHC_16	76	F	3	0	0	IIA	(-)	IHC
IHC_17	54	M	3	1	0	IIB	(+)	IHC
IHC_18	75	F	3	0	0	IIA	(-)	IHC
IHC_19	77	F	2	0	0	IB	(-)	IHC
IHC_20	66	M	3	1	0	IIB	(+)	IHC
IHC_21	69	M	3	1	0	IIB	(+)	IHC
IHC_22	69	M	2	0	0	IB	(+)	IHC
IHC_23	50	F	3	1	0	IIB	(+)	IHC
IHC_24	67	F	3	1	0	III	(+)	IHC
IHC_25	78	M	3	1	0	IIB	(+)	IHC
IHC_26	70	M	3	1	0	IIB	(+)	IHC
IHC_27	59	M	3	0	0	IIA	(-)	IHC
IHC_28	74	F	3	1	0	IIB	(+)	IHC
IHC_29	75	M	3	0	0	IIA	(+)	IHC
IHC_30	58	F	3	0	0	IIA	(+)	IHC
IHC_31	74	M	3	1	0	IIB	(+)	IHC
IHC_32	59	F	3	1	0	IIB	(+)	IHC
IHC_33	69	F	3	0	0	IIA	(-)	IHC
IHC_34	65	F	4	1	0	III	(+)	IHC
IHC_35	85	F	3	1	0	IIB	(+)	IHC

**Table 2:** Annotation of reads aligned to small RNAs

PDAC samples	#T1		#T2		#T3		#T4		#T5		#T6		#T7	
	Count	(%)	Count	(%)	Count	(%)	Count	(%)	Count	(%)	Count	(%)	Count	(%)
Total	17591803	100	15426965	100	16586380	100	10615842	100	18965893	100	23407926	100	12845961	100
exon	1018127	5.79	809048	5.24	184851	1.11	155240	1.46	540260	2.85	459079	1.96	263018	2.05
exon_antisense	9	0.00	4	0.00	5	0.00	6	0.00	3	0.00	4	0.00	10	0.00
miRNA	3045486	17.31	4835786	31.35	3464002	20.88	1721101	16.21	2740681	14.45	2664925	11.38	3152583	24.54
rRNA	489091	2.78	1588798	10.30	216971	1.31	440961	4.15	181910	0.96	583477	2.49	490623	3.82
tRNA	829391	4.71	110334	0.72	1076000	6.49	141156	1.33	1067387	5.63	542804	2.32	263598	2.05
snRNA	24109	0.14	31039	0.20	9153	0.06	14372	0.14	18154	0.10	28997	0.12	24883	0.19
snoRNA	848025	4.82	478768	3.10	617055	3.72	260963	2.46	440008	2.32	1211925	5.18	691475	5.38
lcnRNA	77	0.00	56	0.00	30	0.00	24	0.00	273	0.00	78	0.00	85	0.00
ribozyme	18531	0.11	8449	0.05	7111	0.04	6490	0.06	7255	0.04	16746	0.07	10127	0.08
sRNA	15	0.00	17	0.00	17	0.00	17	0.00	29	0.00	27	0.00	28	0.00
Unannotated	1489059	8.46	2684735	17.40	717881	4.33	5430607	51.16	2993173	15.78	8071185	34.48	4184768	32.58
Unmapped	9829883	55.88	4879931	31.63	10293304	62.06	2444905	23.03	10976760	57.88	9828679	41.99	3764763	29.31

Normal pancreas samples	#N1		#N2		#N3		#N4	
	Count	(%)	Count	(%)	Count	(%)	Count	(%)
Total	16535900	100	20967863	100	17370365	100	18447846	100
Exon	281915	1.70	270434	1.29	576712	3.32	963571	5.22
exon_antisense	2	0.00	9	0.00	39	0.00	24	0.00
miRNA	4247206	25.68	4331251	20.66	3534149	20.35	5133067	27.82
rRNA	659176	3.99	310278	1.48	1457154	8.39	810443	4.39
tRNA	334461	2.02	1145451	5.46	418994	2.41	568066	3.08
snRNA	19163	0.12	22558	0.11	28997	0.17	25124	0.14
snoRNA	703693	4.26	880199	4.20	714087	4.11	641479	3.48
lcnRNA	41	0.00	39	0.00	15	0.00	72	0.00
ribozyme	3967	0.02	7398	0.04	11294	0.07	9258	0.05
sRNA	23	0.00	31	0.00	37	0.00	32	0.00
Unannotated	5295677	32.03	966037	4.61	2152804	12.39	1710926	9.27
Unmapped	4990576	30.18	13034178	62.16	8476083	48.80	8585784	46.54

**Figure 1:**
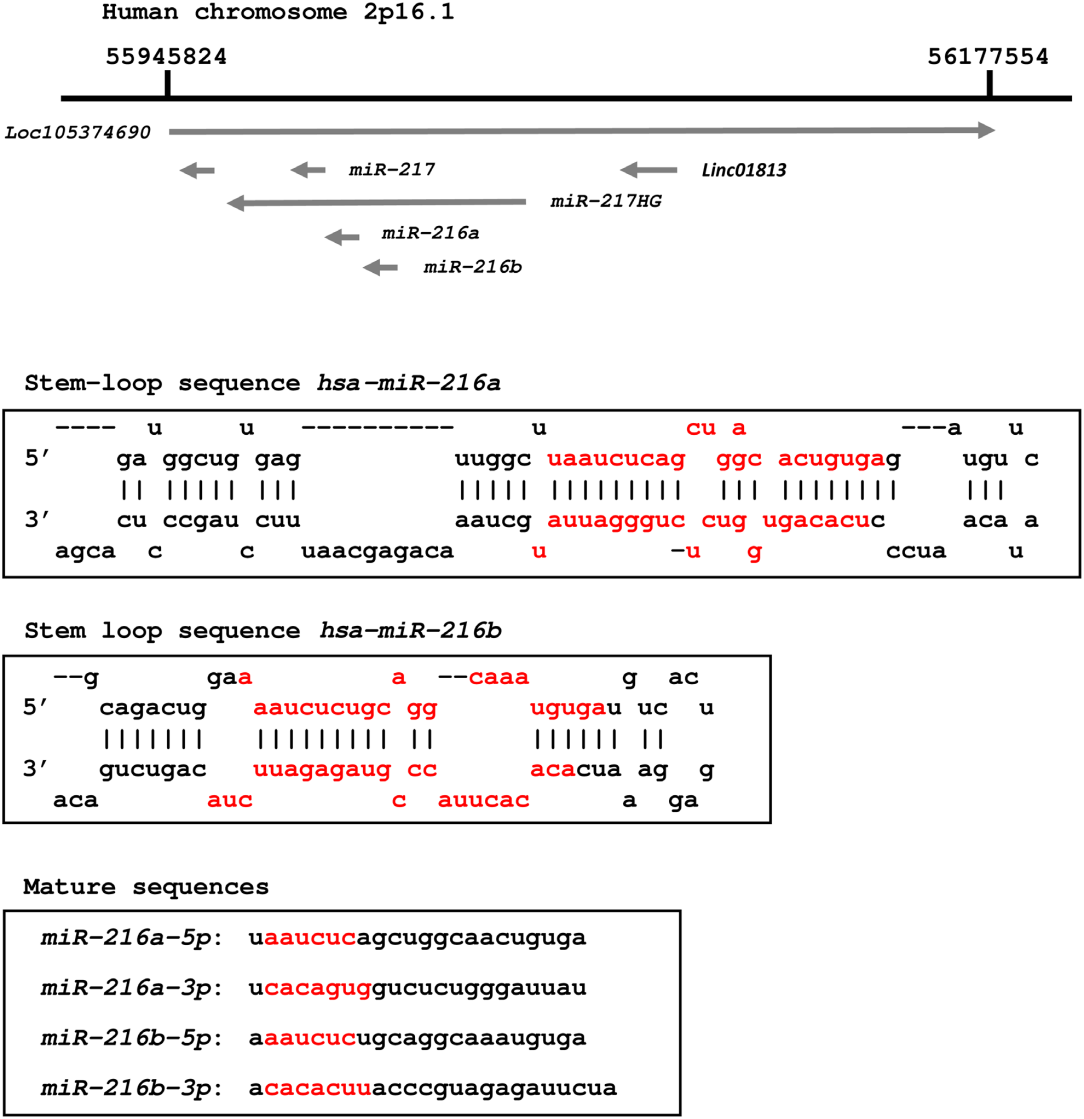
Schematic representation of the human *miR-216* family in chromosomal location The *miR-216* family members and *miR-217* are located on human chromosome 2q16.1. Mature miRNAs, *miR-216a-5p* (guide strand) and *miR-216a-3p* (passenger strand) are derived from pre-*miR-216a*. Likewise pre-*miR-216a*, *miR-216b-5p* (guide strand) and *miR-216b-3p* (passenger strand) are derived from pre-*miR-216b*. Seed sequences of *miR-216a-5p* and *miR-216b-5p* are identical. In contrast, seed sequences of *miR-216a-3p* and *miR-216b-3p* are independent sequences.

We focused on 23 downregulated miRNAs (Table 3), and these miRNAs were mapped on the human genome. We found that *miR-217, miR-216a-5p/-3p* and *miR216b-5p/-3p* were close together and were defined as clustered miRNAs. Because the role of the *miR-216* cluster had not been reported in PDAC, we focused on it in further studies. As shown in Figure 1, *miR-216a-5p/-3p* and *miR216b-5p/-3p* are located within the same chromosomal region (2q16.1).

**Table 3:** Differentially expressed miRNAs in PDAC by RNA seqencing

Downregulated miRNAs in PDAC					
miRNA	miRBase_accession	Locus	LogFC (tumor/normal)	P value	FDR
*hsa-miR-216a-5p*	MIMAT0000273	chr2:56216155-56216176	-3.22	0.0033	0.4602
*hsa-miR-802*	MIMAT0004185	chr21:37093030-37093052	-3.17	0.0001	0.0901
*hsa-miR-3186-3p*	MIMAT0015068	chr17:79418142-79418162	-3.16	0.0072	0.5030
*hsa-miR-217*	MIMAT0000274	chr2:56210155-56210177	-3.13	0.0034	0.4602
*hsa-miR-2114-5p*	MIMAT0011156	chrX:149396251-149396272	-3.08	0.0033	0.4602
*hsa-miR-216b-5p*	MIMAT0004959	chr2:56227899-56227920	-2.77	0.0286	0.9691
*hsa-miR-3186-5p*	MIMAT0015067	chr17:79418178-79418199	-2.70	0.0230	0.9200
*hsa-miR-216b-3p*	MIMAT0026721	chr2:56227859-56227882	-2.58	0.0008	0.1722
*hsa-miR-6510-3p*	MIMAT0025477	chr17:39673418-39673438	-2.57	0.0098	0.6010
*hsa-miR-122-5p*	MIMAT0000421	chr18:56118320-56118341	-2.51	0.0207	0.8791
*hsa-miR-412-3p*	MIMAT0002170	chr14:101531837-101531859	-2.42	0.0393	1.0000
*hsa-miR-129-1-3p*	MIMAT0004548	chr7:127847973-127847994	-2.40	0.0011	0.2082
*hsa-miR-216a-3p*	MIMAT0022844	chr2:56216116-56216137	-2.38	0.0214	0.8791
*hsa-miR-135a-3p*	MIMAT0004595	chr3:52328248-52328269	-2.37	0.0008	0.1722
*hsa-miR-148a-5p*	MIMAT0004549	chr7:25989580-25989601	-2.30	0.0009	0.1722
*hsa-miR-891a-5p*	MIMAT0004902	chrX:145109359-145109380	-2.25	0.0053	0.5030
*hsa-miR-190b*	MIMAT0004929	chr1:154166189-154166209	-2.12	0.0003	0.1429
*hsa-miR-148a-3p*	MIMAT0000243	chr7:25989542-25989563	-2.12	0.0001	0.0901
*hsa-miR-211-5p*	MIMAT0000268	chr15:31357298-31357319	-2.10	0.0084	0.5545
*hsa-miR-1224-5p*	MIMAT0005458	chr3:183959193-183959211	-2.05	0.0450	1.0000
*hsa-miR-338-5p*	MIMAT0004701	chr17:79099723-79099744	-2.02	0.0002	0.0956
*hsa-miR-129-2-3p*	MIMAT0004605	chr11:43603000-43603021	-2.01	0.0090	0.5650
*hsa-miR-137*	MIMAT0000429	chr1:98511647-98511669	-2.01	0.0874	1.0000

**Upregulated miRNAs in PDAC**					
**miRNA**	**miRBase_accession**	**Locus**	**LogFC (tumor/normal)**	**P value**	**FDR**
*hsa-miR-6887-3p*	MIMAT0027675	chr19:35613648-35613668	4.13	0.0007	0.1722
*hsa-miR-4713-5p*	MIMAT0019820	chr15:51534397-51534418	4.04	0.0039	0.4750
*hsa-miR-615-5p*	MIMAT0004804	chr12:54427751-54427772	3.61	0.0053	0.5030
*hsa-miR-1248*	MIMAT0005900	chr3:186504464-186504490	3.50	0.0002	0.0956
*hsa-miR-490-3p*	MIMAT0002806	chr7:136587989-136588010	3.47	0.0386	1.0000
*hsa-miR-184*	MIMAT0000454	chr15:79502182-79502203	3.46	0.0467	1.0000
*hsa-miR-196a-5p*	MIMAT0000226	chr17:46709894-46709915 chr12:54385546-54385567	3.24	0.0103	0.6146
*hsa-miR-187-3p*	MIMAT0000262	chr18:33484798-33484819	3.05	0.0195	0.8676
*hsa-miR-6516-3p*	MIMAT0030418	chr17:75085551-75085572	3.03	0.0046	0.5030
*hsa-miR-490-5p*	MIMAT0004764	chr7:136587952-136587971	2.95	0.0457	1.0000
*hsa-miR-3131*	MIMAT0014996	chr2:219923447-219923469	2.94	0.0112	0.6428
*hsa-miR-4697-3p*	MIMAT0019792	chr11:133768401-133768424	2.90	0.0070	0.5030
*hsa-miR-615-3p*	MIMAT0003283	chr12:54427794-54427815	2.86	0.0001	0.0901
*hsa-miR-196b-5p*	MIMAT0001080	chr7:27209147-27209168	2.73	0.0009	0.1722
*hsa-miR-187-5p*	MIMAT0004561	chr18:33484834-33484855	2.70	0.0610	1.0000
*hsa-miR-6886-3p*	MIMAT0027673	chr19:11224187-11224207	2.68	0.0247	0.9200
*hsa-miR-650*	MIMAT0003320	chr22:23165285-23165305	2.67	0.0138	0.7105
*hsa-miR-492*	MIMAT0002812	chr12:95228203-95228225	2.66	0.0257	0.9200
*hsa-miR-6744-5p*	MIMAT0027389	chr11:1277840-1277858	2.65	0.0169	0.8201
*hsa-miR-6805-5p*	MIMAT0027510	chr19:55899554-55899575	2.55	0.0603	1.0000
*hsa-miR-935*	MIMAT0004978	chr19:54485616-54485638	2.53	0.0059	0.5030
*hsa-miR-4485*	MIMAT0019019	chr11:10529824-10529843	2.53	0.0192	0.8676
*hsa-miR-196a-3p*	MIMAT0004562	chr12:54385583-54385604	2.52	0.0627	1.0000
*hsa-miR-1*	MIMAT0000416	chr18:19408976-19408997 chr20:61151558-61151579	2.48	0.0697	1.0000
*hsa-miR-4768-5p*	MIMAT0019920	chrX:17444012-17444033	2.42	0.0567	1.0000
*hsa-miR-6887-5p*	MIMAT0027674	chr19:35613609-35613631	2.39	0.0749	1.0000
*hsa-miR-181b-3p*	MIMAT0022692	chr1:198828016-198828036	2.34	0.0827	1.0000
*hsa-miR-4745-5p*	MIMAT0019878	chr19:804941-804963	2.32	0.0491	1.0000
*hsa-miR-4724-5p*	MIMAT0019841	chr17:29861911-29861933	2.25	0.0950	1.0000
*hsa-miR-6814-3p*	MIMAT0027529	chr21:43166932-43166953	2.15	0.0664	1.0000
*hsa-miR-203b-3p*	MIMAT0019814	chr14:104583765-104583787	2.10	0.0518	1.0000
*hsa-miR-143-5p*	MIMAT0004599	chr5:148808507-148808528	2.09	0.0019	0.3270
*hsa-miR-6850-5p*	MIMAT0027600	chr8:146017355-146017376	2.09	0.1212	1.0000
*hsa-miR-549a*	MIMAT0003333	chr15:81134334-81134354	2.06	0.1130	1.0000
*hsa-miR-133a-3p*	MIMAT0000427	chr18:19405673-19405694 chr20:61162177-61162198	2.06	0.0275	0.9691
*hsa-miR-3661*	MIMAT0018082	chr5:133561468-133561489	2.03	0.0800	1.0000
*hsa-miR-4649-5p*	MIMAT0019711	chr7:44150450-44150473	2.02	0.0661	1.0000
*hsa-miR-31-3p*	MIMAT0004504	chr9:21512120-21512141	2.02	0.0195	0.8676
*hsa-miR-6862-5p*	MIMAT0027625	chr16:28402346-28402367 chr16:28735578-28735599	2.01	0.0770	1.0000

### Expression levels of the *miR-216* family in PDAC specimens and cell lines

**Figure 2:**
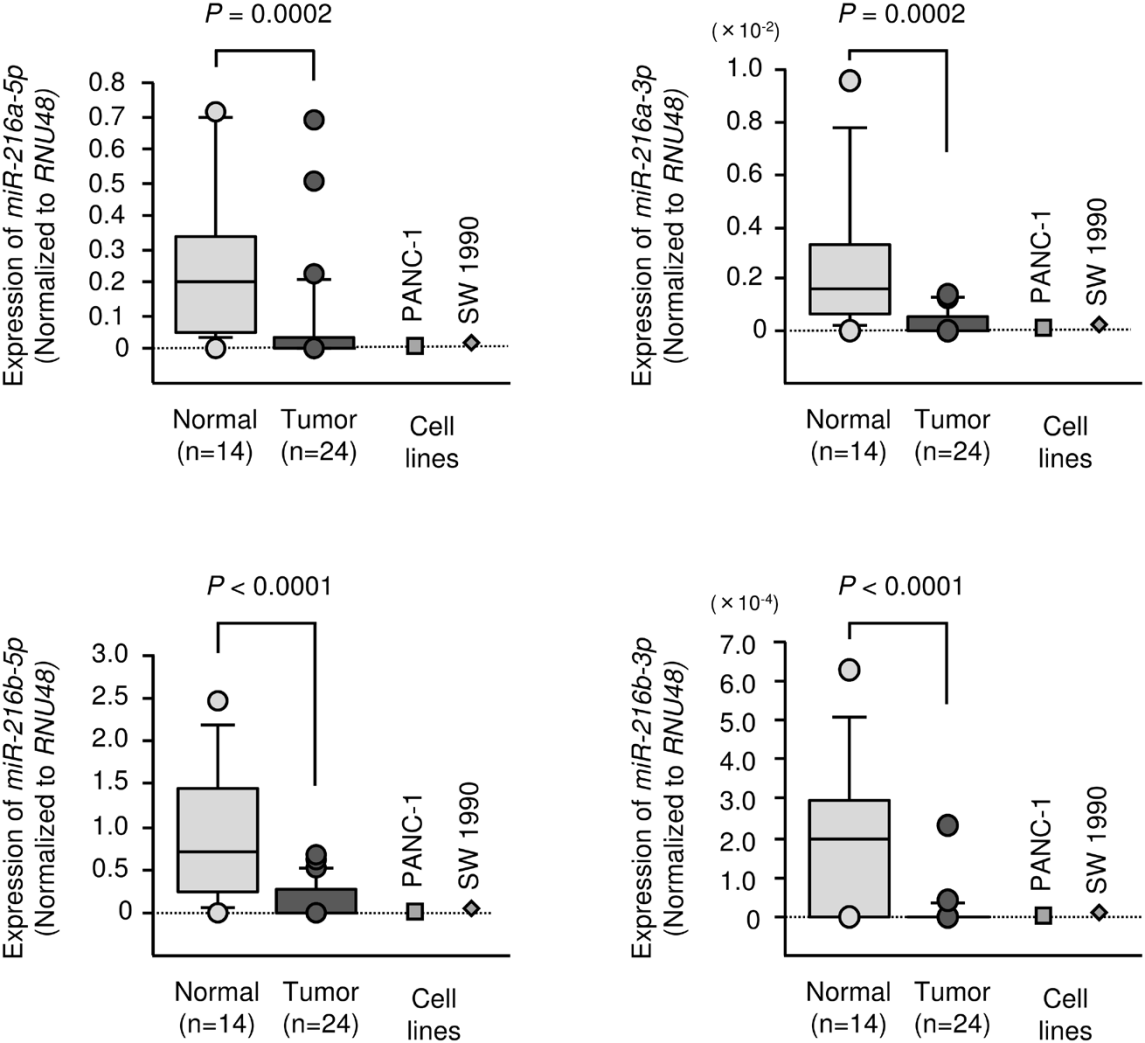
Expression levels of *miR-216* family members Expression levels of *miR-216* family members in clinical specimens and PDAC cell lines were determined using qRT-PCR. Data were normalized to *RNU48* expression.

We evaluated expression levels of the *miR-216 family* in PDAC tissues (n = 24), normal pancreatic tissues (n = 14) and two PDAC cell lines (PANC-1 and SW 1990). Patient clinicopathological features are summarized in Table 1. The expression levels of *miR-216a-5p, miR-216a-3p, miR-216b-5p and miR-216b-3p* were significantly lower in tumour tissues than in normal pancreatic tissues (Figure 2). It was not recognized the significant relationships between any of the clinical parameters (i.e., TNM stage, metastasis or survival rate) and expression levels of any member of the *miR-216*-family (data not shown).

To elucidate molecular mechanisms of low expression of the clustered miRNAs, *miR-216a-5p*, *miR-216a-3p*, *miR-216b-5p* and *miR-216b-3p* in PDAC cells, PANC-1 and SW1990 cells were treated with the demethylating agent [5-aza-2’-deoxycytidine (5-aza-dC)]. Expression levels of all clustered miRNAs in PDAC cells were significantly elevated by 5-aza-dc treatment (Supplementary Figure 1). These data suggested that DNA methylation might cause silencing of these clustered miRNAs in PDAC cells.

### Expression of *miR-216* family members and the effects on cell growth, migration and invasion in PDAC cell lines

**Figure 3:**
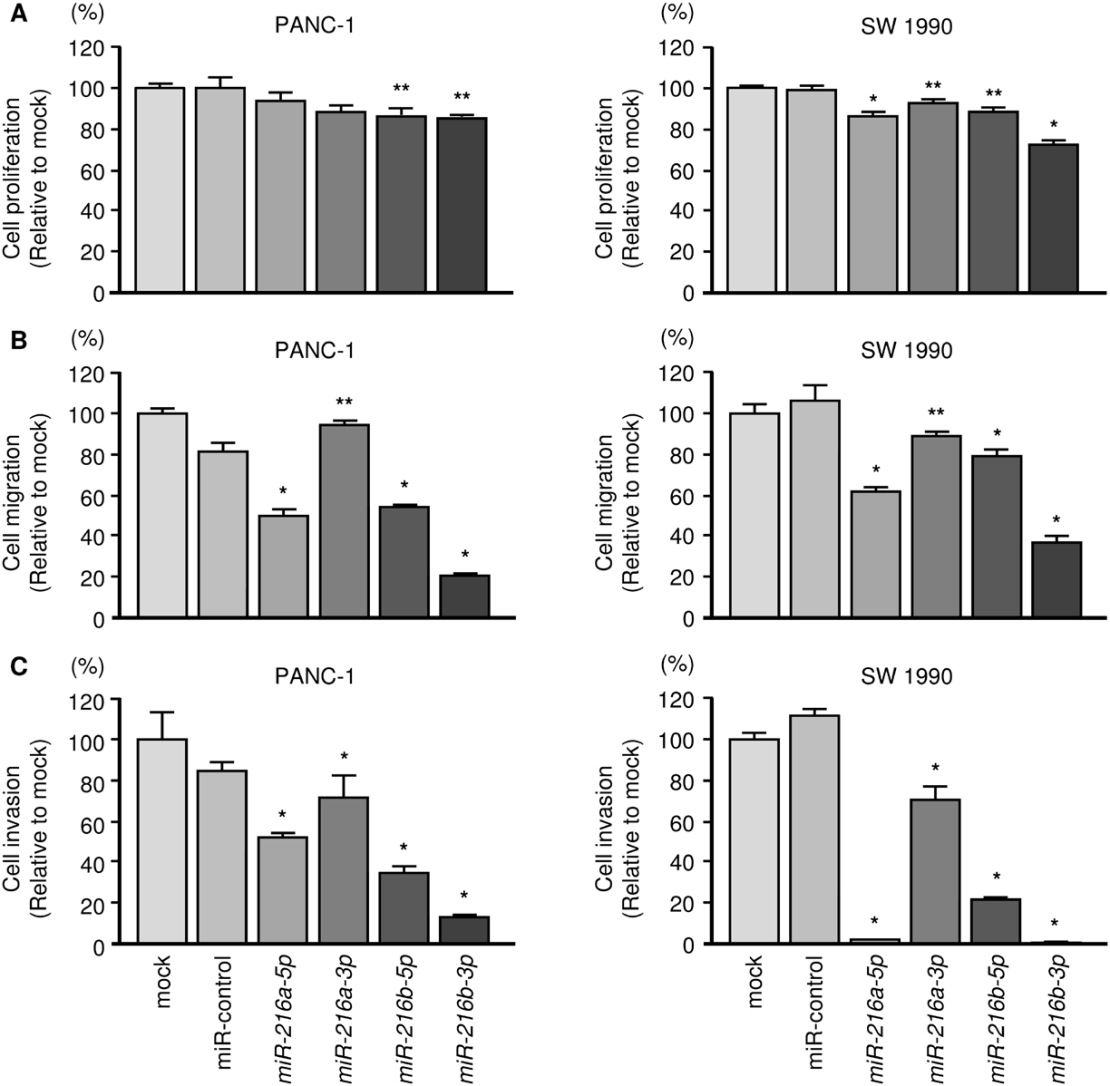
Effects of *miR-216a-5p, miR-216a-3p, miR-216b-5p* and *miR-216b-3p* transfection into PDAC cells **(A)** Cell growth was determined using XTT assays 72 h after transfection with 10 nM *miR-216* family members. *, *P* < 0.0001; **, *P* < 0.05. **(B)** Cell migration activity was determined using BD Falcon Cell Culture Inserts. *, *P* < 0.0001. **(C)** Cell invasion activity was determined using Matrigel invasion assays. *, *P* < 0.0001.

The functional roles of the *miR-216*-family were performed by using restoration of mature miRNAs assays. Functional assays showed that proliferation, migration and invasion activities of cancer cells were significantly suppressed in *miR-216a-5p*, *miR-216a-3p*, *miR-216b-5p* and *miR-216b-3p* transfectants compared with mock or miR-control transfectants (Figure 3). Present results suggested that the *miR-216* family could have anti-tumour functions in PDAC cells. Among the *miR-216* family, *miR-216b-3p* markedly inhibited PDAC cell aggressiveness (Figure 3).

We hypothesized that passenger strand of *miR-216-3p* may be incorporated into and function as part of the RISC structure. To check this hypothesis, we performed immunoprecipitation with antibody targeting Ago2, which plays a pivotal role in the RISC. After transfection with *miR-216-3p*, Ago2-bound miRNAs were isolated, and RT-qPCR was carried out to determine whether *miR-216-3p* bound to Ago2. Expression of *miR-216-3p* levels were significantly higher than those of mock and miRNA control transfected cells (Supplementary Figure 2). Moreover, expression levels of *miR-216a-5p*, *miR-216a-3p* and *miR-216b-5p* were same levels of mock and miRNA control transfectants (Supplementary Figure 2).

### Identification of genes regulated by *miR-216b-3p* in PDAC cells

**Figure 4:**
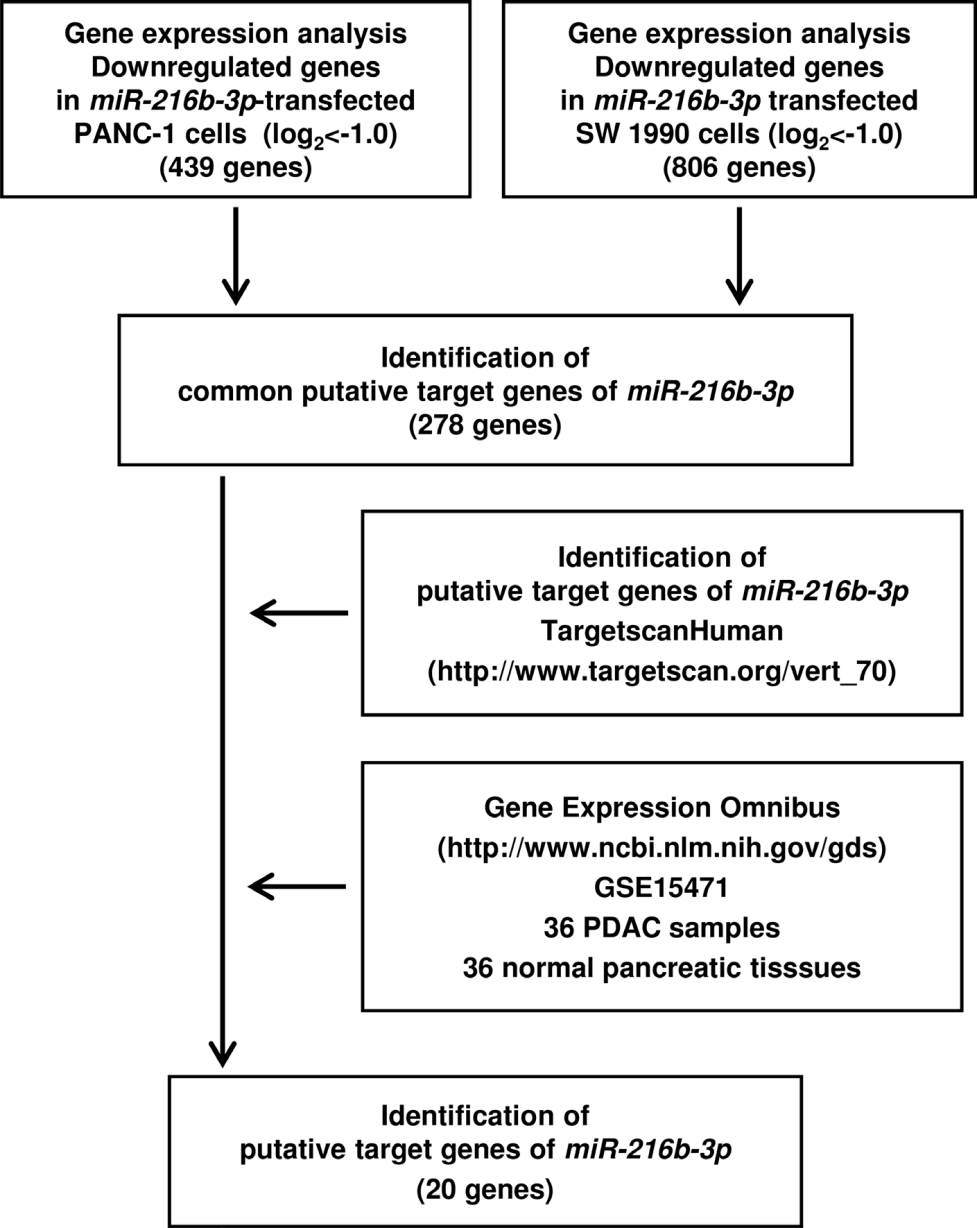
Flow chart illustrating the analytic strategy for *miR-216b-3p* target genes Strategy for identification of putative candidate genes regulated by *miR-216b-3p* in PDAC cells. Approach to identifying *miR-216b-3p* target genes by *in silico* analysis of genome-wide gene expression, TargetScan and GEO database analysis of *miR-216b-3p* transfectants and PDAC clinical specimens.

To elucidate the molecular mechanisms and pathways regulated by anti-tumour *miR-216b-3p* in PDAC cells, we used a combination of gene expression and *in silico* analyses. Figure 4 presents the strategy for narrowing down the target genes of *miR-216b-3p*. In gene expression analyses, 439 and 806 genes were downregulated (log_2_ ratio <-1.0) in PANC-1 and SW 1990 *miR-216b-3p* transfectants, respectively, in comparison with control transfectants (GEO accession number, GSE82108). Next, we pared down the list of genes using the TargetscanHuman and GEO database (GEO accession number, GSE15471). We found that 20 genes were putatively targeted by *miR-216b-3p* in PANC-1 and SW 1990 *miR-216b-3p* transfectants.

Among these candidate genes, 6 genes (*PLAU*, *FOXQ1*, *PGM2L1*, *MBOAT2*, *PERP* and *UHMK1*) were significantly associated with poor prognosis of patients with PDAC (Table 4 and Supplementary Figure 3). Past studies showed that aberrant expressed FOX-family of transcription factors were involved in several types of cancers, however, functional significance of *FOXQ1* in PDAC is still unclear.

**Table 4:** Candidate target genes regulated by miR-216b-3p in PDAC

Entrez gene ID	Gene symbol	Description	Microarray (Log_2_ ratio) *miR-216b-3p*			Target site (Targetscan)	GEO	TCGA-OncoLncKaplan plot P-value
			PANC-1	SW1990	Average	*miR-216b-3p*	FC	
5328	*PLAU*	plasminogen activator, urokinase	-1.03	-1.10	-1.06	(+)	5.07	0.0161
59345	*GNB4*	guanine nucleotide binding protein (G protein), beta polypeptide 4	-1.76	-2.27	2.01	(+)	4.57	0.3330
94234	*FOXQ1*	forkhead box Q1	-1.50	-1.52	-1.51	(+)	3.88	0.0089
9240	*PNMA1*	paraneoplastic Ma antigen 1	-1.24	-1.69	-1.47	(+)	3.27	0.9910
283209	*PGM2L1*	phosphoglucomutase 2-like 1	-1.67	-1.91	-1.79	(+)	3.23	0.0477
60481	*ELOVL5*	ELOVL fatty acid elongase 5	-1.50	-2.77	-2.13	(+)	3.15	0.4970
129642	*MBOAT2*	membrane bound O-acyltransferase domain containing 2	-1.20	-1.40	-1.30	(+)	2.92	0.0158
5912	*RAP2B*	RAP2B, member of RAS oncogene family	-1.31	-1.25	-1.28	(+)	2.74	0.1040
5738	*PTGFRN*	prostaglandin F2 receptor inhibitor	-1.57	-2.00	-1.79	(+)	2.64	0.4350
493	*ATP2B4*	ATPase, Ca++ transporting, plasma membrane 4	-1.45	-2.27	-1.86	(+)	2.55	0.0966
54492	*NEURL1B*	neuralized E3 ubiquitin protein ligase 1B	-1.32	-1.95	-1.64	(+)	2.36	0.0365
64065	*PERP*	PERP, TP53 apoptosis effector	-1.78	-1.98	-1.88	(+)	2.33	<0.0001
160418	*TMTC3*	transmembrane and tetratricopeptide repeat containing 3	-1.85	-2.24	-2.05	(+)	2.30	0.9080
55589	*BMP2K*	BMP2 inducible kinase	-1.04	-1.54	-1.29	(+)	2.29	0.5220
3977	*LIFR*	leukemia inhibitory factor receptor alpha	-1.96	-2.09	-2.03	(+)	2.26	0.0062*
10920	*COPS8*	COP9 signalosome subunit 8	-1.43	-2.04	-1.73	(+)	2.20	0.2740
9637	*FEZ2*	fasciculation and elongation protein zeta 2 (zygin II)	-1.03	-1.28	-1.15	(+)	2.18	0.0974
4286	*MITF*	microphthalmia-associated transcription factor	-1.02	-1.07	-1.05	(+)	2.17	0.0963
29967	*LRP12*	low density lipoprotein receptor-related protein 12	-1.32	-2.22	-1.77	(+)	2.09	0.6200
127933	*UHMK1*	U2AF homology motif (UHM) kinase 1	-1.20	-1.79	-1.50	(+)	2.04	0.0329

* Poor prognosis with a low expression

**Figure 5:**
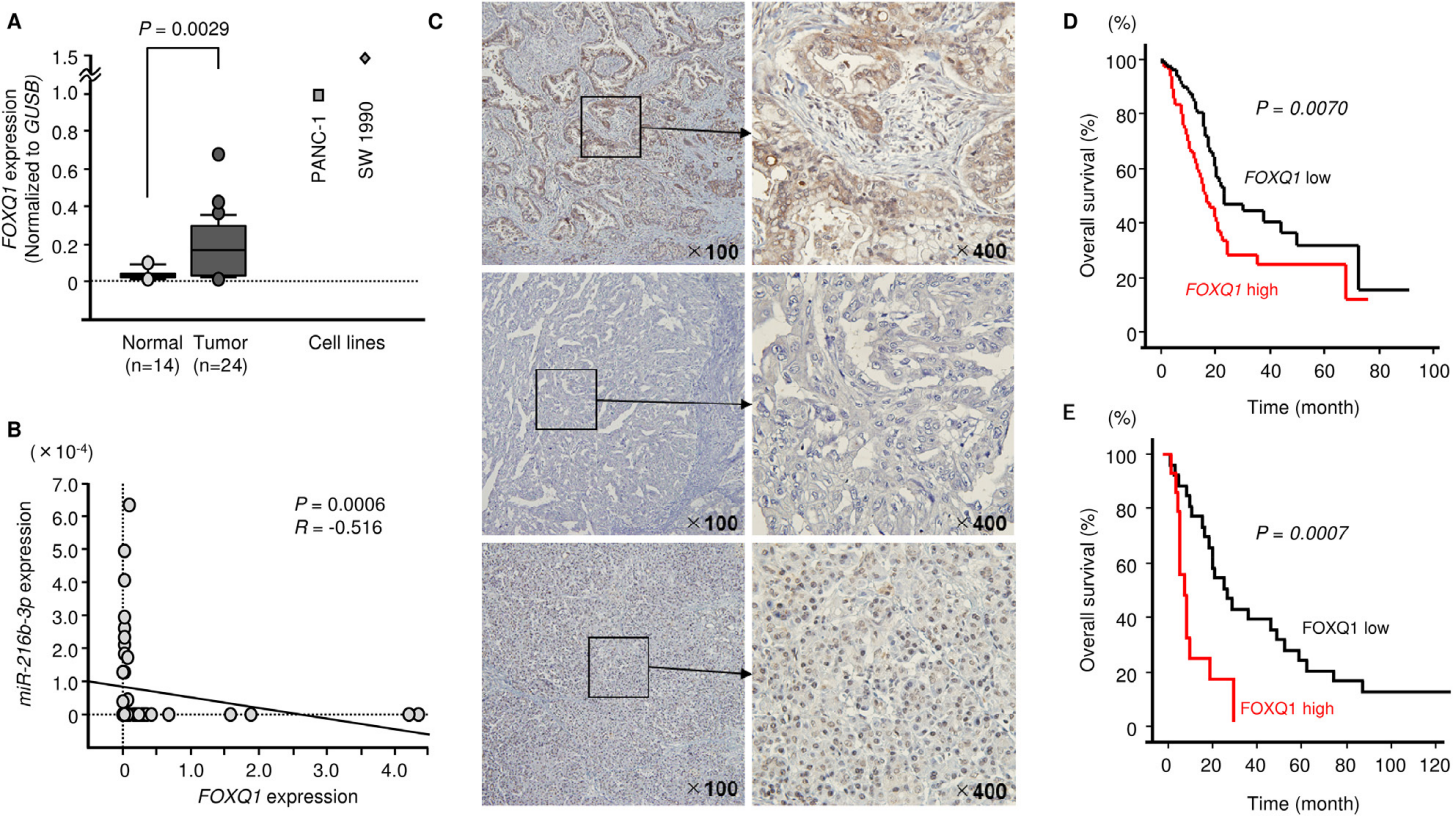
Expression of FOXQ1 (mRNA and protein) in PDAC and association of FOXQ1 with overall survival **(A)** Expression levels of *FOXQ1* in clinical specimens and PDAC cell lines were determined using qRT-PCR. Data were normalized to *GUSB* expression. **(B)** Correlation between *miR-216-3p* and *FOXQ1* expression. **(C)** Immunohistochemical staining of FOXQ1 in PDAC clinical specimens. Overexpression of FOXQ1 was observed in cancer lesions (original magnification ×400). In contrast, negative staining of FOXQ1 was observed in normal tissues. (IHC_13) Positively stained cancer lesion (T3N1M0). (IHC_18) Negatively stained cancer lesion (T3N0M0). (IHC_29) Negatively stained normal pancreas tissue. **(D)** TCGA-based large cohort study of high expression of FOXQ1 in 174 PDAC patients. **(E)** Kaplan-Meier patient survival curves for overall survival rates based on FOXQ1 expression in 35 patients with PDAC. P-values were calculated using the log-rank test.

We determined that *FOXQ1* was upregulated in clinical PDAC samples using qRT-PCR (Figure 5A). Negative correlations between *miR-216b-3p* expression and *FOXQ1* mRNA expression were found using Spearman’s rank test (*R* = -0.5160, *P* = 0.0006, Figure 5B). Kaplan–Meier survival curves constructed from PDAC patients in the TCGA dataset showed that patient groups with high expression of *FOXQ1* had significantly shorter overall survival (*P* = 0.0070; Figure 5D). The TCGA dataset was analysed by OncoLnc database (http://www.oncolnc.org/)[24].

### Expression of FOXQ1 in PDAC clinical specimens

We validated the expression of FOXQ1 in PDAC clinical specimens using immunohistochemistry. A total of 35 specimens were evaluated, and 12 samples were classified as having high expression of FOXQ1. Clinicopathological characteristics are summarized in Table 1. Table 5 shows the correlation between FOXQ1 expression and various clinicopathological factors. High FOXQ1 expression was significantly associated with increased lymph node metastasis. Furthermore, patients with high FOXQ1 expression had significantly shorter OS than those with low FOXQ1 expression (*P =* 0.0007) (Figure 5E).

**Table 5:** Correlation between the expression of FOXQ1 and clinicopathological factors in PDAC (n =35)

Characteristic	FOXQ1		*P*
	Low (n = 23)	High (n = 12)	
Age (y)^a^			^b^NS
≥ 60	17	10	
< 60	6	2	
Gender (n)			
Male (20)	13	7	NS
Female (15)	10	5	
Tumor size (n)			
> 40 mm (5)	2	3	NS
≤ 40 mm (30)	21	9	
Lymph node metastasis (n)			
No (11)	10	1	0.0088
Yes (24)	11	13	
Distant metastasis (n)			
No (35)	23	12	NS
TNM Stage (n)			
I / II (27)	18	9	NS
III / IV (8)	5	3	
Recurrence (n)			
No (8)	6	2	NS
Yes (27)	17	10	

^a^Values are mean ± SD.

^b^NS, not significant.

Moreover, we categorized patients into two groups, with adjuvant chemotherapy or without adjuvant chemotherapy. Regardless of the treatment, patients with high FOXQ1 expression were associated with poor prognosis (Supplementary Figure 4).

We confirmed the expression of FOXQ1 in PDAC clinical specimens using immunohistochemistry. Expression of FOXQ1 was detected in in ductal carcinoma, not in normal ductal cells, acinar cells and islets cells (Supplementary Figure 5).

### *FOXQ1* is a direct target of *miR-216b-3p* in PDAC cells

**Figure 6:**
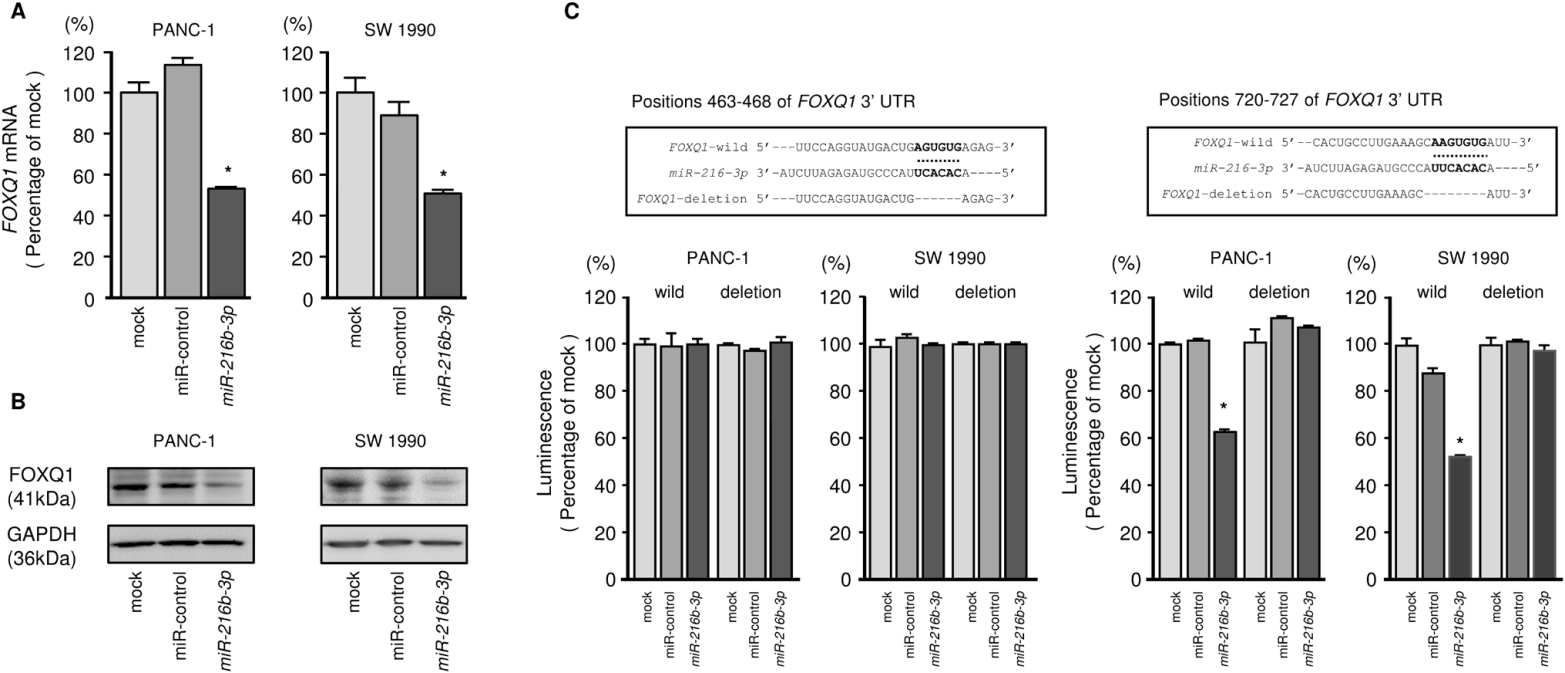
Direct regulation of FOXQ1 by *miR-216b-3p* in PDAC cells **(A)** *FOXQ1* mRNA expression was evaluated using qRT-PCR in PANC-1 and SW 1990 cells 72 h after transfection with *miR-216b-3p*. *GUSB* was used as an internal control. *, *P* < 0.0001. **(B)** FOXQ1 protein expression was evaluated by Western blotting in PANC-1 and SW 1990 cells 72 to 96 h after transfection with *miR-216b-3p*. GAPDH was used as a loading control. **(C)** *miR-216b-3p* binding sites in the 3’-UTR of *FOXQ1* mRNA. Dual luciferase reporter assays using vectors encoding putative *miR-216b-3p* target sites of the *FOXQ1* 3’-UTR (positions 463-468 and 720-727) for both wild-type and deleted regions. Data were normalized by expressing ratios of *Renilla*/firefly luciferase activities. *, *P* < 0.0001.

We performed qRT-PCR to validate *miR-216b-3p*-mediated reduction of *FOXQ1* mRNA expression in PDAC cell lines. Our studies demonstrated that *FOXQ1* mRNA was markedly reduced in *miR-216b-3p* transfectants compared to mock or miR-control transfectants (P < 0.0001, Figure 6A). FOXQ1 protein expression was also repressed in the *miR-216b-3p* transfectants (Figure 6B).

Target prediction databases indicated the presence of 2 putative target sites in the 3'-UTR of *FOXQ1* (Figure 6C). To determine whether *FOXQ1* mRNA had a functional target site, we performed luciferase reporter assays. Compared with the miR-control, luminescence intensity was significantly reduced by transfection with *miR-216b-3p* at its target site, position 720-727 in the 3’-UTR of *FOXQ1* (Figure 6C).

### Effects of silencing *FOXQ1* on PDAC cell lines

**Figure 7:**
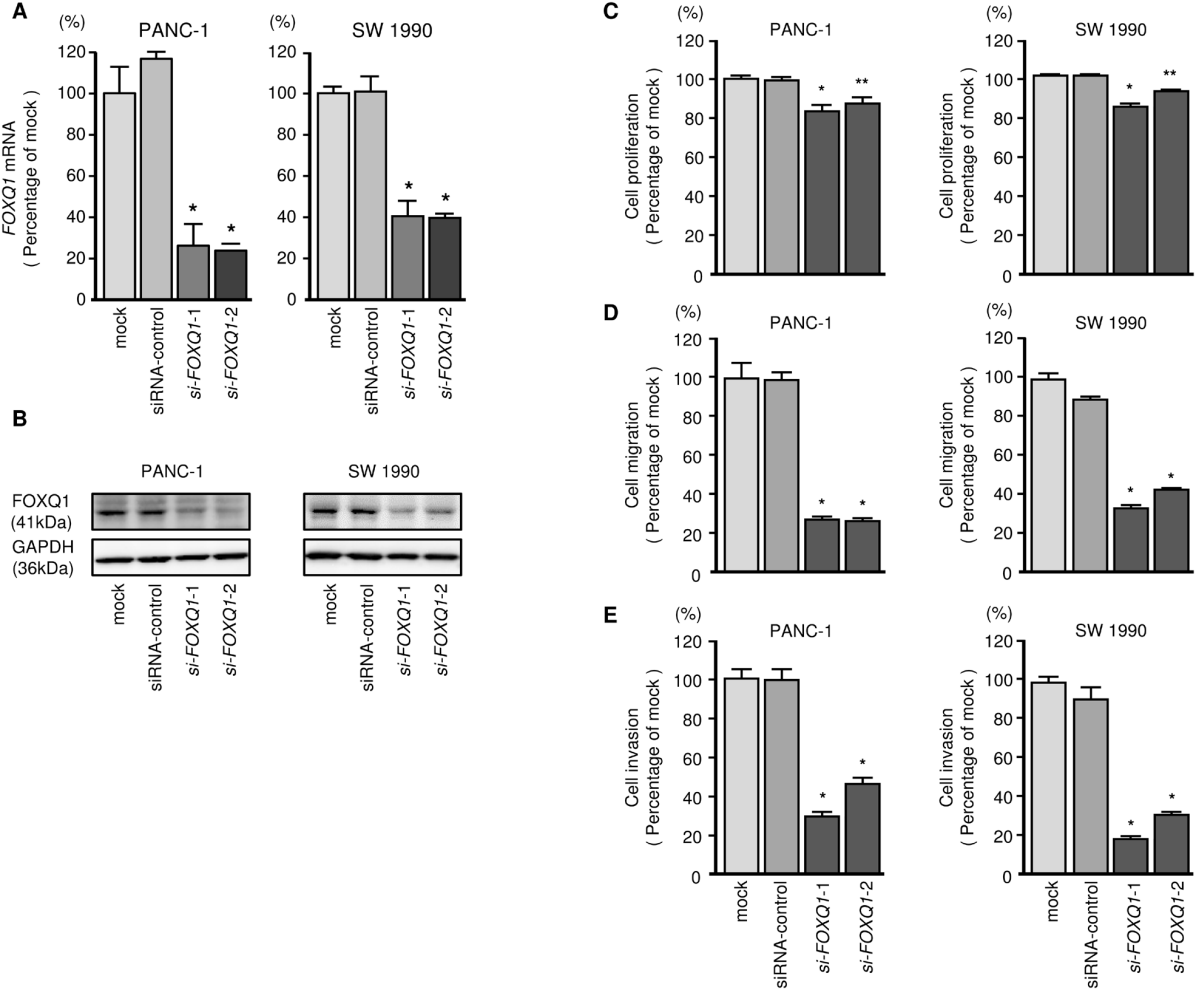
Effects of FOXQ1 silencing on PDAC cell lines **(A)** *FOXQ1* mRNA expression was evaluated using qRT-PCR analysis of PANC-1 and SW 1990 cells 72 h after transfection with *si-FOXQ1*-1 or *si-FOXQ1*-2. *GUSB* was used as an internal control. **(B)** FOXQ1 protein expression was evaluated using Western blotting analysis of PANC-1 and SW 1990 cells 72 to 96 h after transfection with *miR-216b-3p*. GAPDH was used as a loading control. **(C)** Cell proliferation was determined using XTT assays 72 h after transfection with 10 nM *si-FOXQ1*-1 or *si-FOXQ1*-2. *, *P* < 0.0001; **, *P* < 0.05. **(D)** Cell migration activity was determined using BD Falcon Cell Culture Inserts. *, *P* < 0.0001. **(E)** Cell invasion activity was determined using Matrigel invasion assays. *, *P* < 0.0001.

To investigate the oncogenic functions of *FOXQ1* in PDAC cells, we carried out knockdown assays by using si-*FOXQ1*. First, we evaluated the knockdown efficiency achieved by si-*FOXQ1* transfection of PDAC cell lines. We used 2 types of si-*FOXQ1* (si-*FOXQ1-1* and si-*FOXQ1-2*) in this study. Both siRNAs effectively downregulated *FOXQ1* expression in bothcell lines (Figure 7A and 7B). Functional assays showed that si-*FOXQ1* transfection significantly reduced cell proliferation, migration and invasion compared with mock- or siRNA-control-transfected cells (Figure 7C-7E).

In contrast to loss-of-function assays, overexpression of *FOXQ1* enhanced cancer cell migration and invasion in PANC-1 cells (Supplementary Figure 6).

### Investigation of *FOXQ1*-mediated downstream genes in PDAC cells

Genome-wide gene expression (GEO accession number, GSE77790) and *in silico* analyses were performed to investigate the downstream genes mediated by *FOXQ1* in a PDAC cell line (PANC-1) transfected with si-*FOXQ1*. A total of 18,277 genes were commonly downregulated (log_2_ ratio < 0). Further GEO analyses showed that 1,003 genes upregulated expression (log_2_ ratio > 1.0) in GEO accession number GSE15471. Next, we assigned genes to KEGG pathways using the GENECODIS program and identified 261 pathways. In the top 3 pathways, 41 genes were significantly enriched (Supplementary Tables 3A and 3B).

TCGA database analyses demonstrated that high expression of 13 genes were significantly associated with poor survival in patients with PDAC (Supplementary Figure 7).

## DISCUSSION

miRNA fine-tunes the expression control of protein coding/noncoding RNAs by repressing translation or cleaving RNA transcripts. Dysregulated miRNAs can disrupt tightly controlled RNA networks and thereby enhance the aggressiveness of cancer cells. Identification of dysregulated miRNAs and their targeted genes can improve our understanding of the molecular pathogenesis of human cancers. In that regard, we have identified novel RNA networks in several cancers [13–15].

In the present study, we newly constructed a miRNA expression signature of PDAC by RNA sequencing methods. Several miRNA signatures of PDAC were reported in which formalin-fixed paraffin-embedded tissue or fine-needle aspiration biopsies specimens were used for PCR or microarray-based applications [25–27]. Recently, we summarized dysregulated miRNAs in PDAC using information in public databases [12]. Downregulation of *miR-217*, *miR-141*, *miR-148a* and *miR-375* in PDAC tissues was commonly observed in PDAC tissues [12]. More recently, we demonstrated the anti-tumour roles of *miR-375* through its targeting of *ZFP36L2* in PDAC cells [28]. Our present signature included several anti-tumour miRNAs that had been previously identified in PDAC, suggesting that our RNA-based signature was effective for identification of novel oncogenes and anti-tumour miRNAs in PDAC cells. These data may provide a benchmark for future studies of PDAC.

Our signature revealed that novel miRNAs were aberrantly expressed in PDAC cells. Among them, we focused on 4 clustered miRNAs, *miR-216a-5p*, *miR-216a-3p*, *miR-216b-5p* and *miR-216b-3p* located on human chromosome 2p16.1. Here, we showed that the clustered *miR-216* family has anti-tumour activity in PDAC. Those results suggest that the *miR-216* family is important in PDAC oncogenesis. Past studies showed that guide strands of *miR-216a-5p* and *miR-216b-5p* acted as anti-tumour miRNAs in several cancers [29, 30]. On the other hand, the functional significance of passenger strands *miR-216a-3p* and *miR-216b-3p* has not been adequately analysed.

In miRNA biogenesis, the RNA guide strand from the duplex is recruited to the RNA-induced silencing complex (RISC) to target messenger RNAs, whereas the passenger strand is degraded and no functional activity [31, 32]. Nevertheless, our present data showed that passenger strands *miR-216a-3p* and *miR-216b-3p* have anti-tumour functions in PDAC cells. Recently, we demonstrated that both strands of *pre-miR-145* (*miR-145-5p*, guide strand, and *miR-145-3p*, passenger strand) were incorporated into RISC and function anti-tumour miRNAs by targeting several oncogenes in bladder and lung cancer cells [21, 22]. The involvement of passenger strand miRNAs in the regulation of cellular processes is a novel concept in RNA research.

The *Kras*^G12D^ oncogene is a key oncogenic mutation that is associated with development of PDAC [33, 34]. A recent study evaluated miRNA expression in the *Kras*^G12D^ transgenic mouse model of PDAC [35]. In that study, 3 miRNAs (*miR-216a*, *miR-216b*, and *miR-217*) were reduced in expression in the *Kras*^G12D^ pancreas. Other study showed that significantly downregulation of *miR-216a* and *miR-217* was detected in Kras^G12d^;Pdx1-Cre mouse at 25 weeks [36]. Due to the lack of human clinical specimens from early stages of PDAC, the investigation is important to analyze expression patterns of miRNAs in early stages of PDAC by using mouse model. The molecular mechanisms of downregulation of these clustered miRNAs are still obscure.

To better understand and identify the anti-tumour targets of *miR-216b-3p* in PDAC cells, we have applied *in silico* database and genome-wide gene expression analyses. In this study, *FOXQ1* (a member of the forkhead transcription factor family) was identified as a direct target of *miR-216b-3p* regulation in PDAC cells. Moreover, our functional studies revealed that expression of *FOXQ1* promoted cancer cell aggressiveness, and overexpression of *FOXQ1* was involved in PDAC pathogenesis. The FOX-family consists of a large number of transcription factors that have been categorized in 19 subfamilies, FOXA-FOXS [37, 38]. FOX transcription factors play pivotal roles in regulating the expression of genes involved in several biological processes [37, 38]. Numerous studies have demonstrated that *FOXQ1* (as well as other FOX-family member) is associated with the progression, metastasis and drug resistance of human cancers [39].

The epithelial-mesenchymal transition (EMT) is pivotal in cancer cell invasion, metastasis and chemoresistance [40, 41]. Many EMT-promoting transcription factors have been implicated in these events in cancer cells [40, 41]. In lung cancer cells, elimination of *FOXQ1* affected the re-expression of epithelial markers and decreases mesenchymal markers *in vitro* and *in vivo*[42]. Similar studies reported that upregulation of *FOXQ1* triggered EMT and contributed to metastasis and chemoresistance in several cancers [43–45]. The existence of cancer stem cells (CSCs) is an important concept in cancer cell aggressiveness, metastasis and chemoresistance [46]. In PDAC, CSCs were isolated based on expression of CD44, CD133 and EpCAM. Overexpression of FOXQ1 was detected in CSCs and CSCs behaver have more aggressiveness for cell growth, compared with parental MiaPaCa-2 cells [47].

A large number of cohort analyses of the TCGA dataset showed that high expression of *FOXQ1* predicted shortened survival of patients with PDAC. Likewise, there are many reports that expression of *FOXQ1* indicates poor prognosis [44, 45, 48]. In this study, we identified “ECM-receptor interaction”, “regulation of actin cytoskeleton” and “focal adhesion pathways” as *FOXQ1*-mediated downstream pathways in PDAC cells. Interestingly, among 41 downstream genes of *FOXQ1*, 13 genes were associated with poor prognosis of patients with PDAC by TCGA analyses. These findings suggest that expression of *FOXQ1* and *FOXQ1*-mediated pathways contribute significantly to PDAC aggressiveness.

Our miRNA signature showed that *miR-217* was also markedly reduced in PDAC tissues. This miRNA is located in the same genomic region as the *miR-216* cluster. Our data showed that ectopic expression of *miR-217* significantly inhibited cancer cell aggressiveness in PDAC cells by targeting focal adhesion pathways (data not shown). Past studies pointed to the anti-tumour function of *miR-217* in several cancers, including the targeting of the *KRAS* oncogene in PDAC [49].

In conclusion, downregulation of 4 clustered miRNAs (*miR-216a-5p*, *miR-216a-3p*, *miR-216b-5p* and *miR-216b-3p*) was detected by RNA sequencing-based miRNA signature analysis. The tumour-suppressor functions of these miRNAs were shown in PDAC cells. The expression of *FOXQ1* was directly regulated by *miR-216b-3p* and its overexpression was involved in PDAC pathogenesis. Identification of novel cancer networks mediated by aberrantly expressed miRNAs and tumour-suppressor miRNAs may improve our understanding of PDAC molecular pathogenesis. Our newly created RNA sequencing-based miRNA signature provides a basis for further PDAC research.

## MATERIALS AND METHODS

### Clinical specimens and cell lines

We used fresh (n = 24) and preserved (formalin-fixed, paraffin-embedded blocks) (n = 35) specimens for the current study. Tissues were obtained from PDAC patients who underwent surgery at Kagoshima University Hospital between 1991 and 2014. Normal pancreatic tissue specimens (n = 14) were collected from noncancerous tumour-adjacent tissue. Each surgical specimen was histologically classified according to the TNM classification system [50]. All patients in this study provided informed consent and the study protocol was approved by the Institutional Review Board of Kagoshima University. Patient information and clinicopathological features are shown in Table 1.

Two human PDAC cell lines were used in this study. PANC-1 cells were obtained from the RIKEN Cell Bank (Tsukuba, Ibaraki, Japan) and SW 1990 cells were obtained from the ATCC (Manassas, Virginia, USA).

### Construction of the miRNA expression signature in PDAC

Total RNAs from 7 PDAC tissues and 4 normal pancreatic tissues were subjected to RNA sequencing. The construction procedure of miRNA signature included the following steps: small RNA isolation, cDNA library preparation and sequencing as described previously [14]

### Quantitative real-time RT-PCR

Quantification of miRNA was performed using qRT-PCR as previously described [20, 28, 51, 52]. We used the following probes and primers: *miR-216a-5p* (product ID: 2220; Thermo Fisher Scientific, Waltham, MA, USA), *miR-216a-3p* (product ID: 475580_mat), *miR-216b-5p* (product ID: 2326), *miR-216b-3p* (product ID: 467177_mat), *PLAU* (product ID: Hs01547054_m1), *GNB4* (product ID: Hs00222147_m1) and *FOXQ1* (product ID: Hs00536425_s1. Human *GUSB* (product ID: Hs99999908_m1) and *RNU48* (product ID: 001006) were used as internal controls.

### Transfection of miRNA mimic and small interfering RNA (siRNA) into PDAC cell lines

PDAC cell lines were transfected with a miRNA mimic for gain-of-function experiments and siRNA for loss-of-function experiments. Pre-miR^™^ miRNA precursors for *miR-216a-5p* (product ID: PM10545; Thermo Fisher Scientific), *miR-216a-3p* (product ID: PM24316), *miR-216b-5p* (product ID: PM12302), *miR-216b-3p* (product ID: PM28358), negative control miRNA (product ID: AM 17111), two *FOXQ1* siRNAs (product IDs: s41290 and s41291) and control siRNA (product ID: D-001810-10) were purchased from Thermo Fisher Scientific. The transfection efficiencies of miRNA in PANC-1 and SW 1990 cells were calculated as described in previous studies [20, 28, 51, 52].

### Cell proliferation, migration and invasion assays

Cell proliferation, migration and invasion assays were described previously [20, 28, 51, 52].

### Western blot analyses

Mouse anti-FOXQ1 antibodies (product ID: sc-166266; Santa Cruz Biotechnology, Inc., Dallas, TX, USA) were diluted 1:500 for immunoblotting. Anti-GAPDH antibodies at a 1:1000 dilution (product ID: 010-25521; Wako, Osaka, Japan) were used as an internal loading control.

### Immunohistochemistry

Tissue sections were incubated overnight at 4°C with anti-FOXQ1 antibodies diluted 1:300 (product ID: ab51340; Abcam, Cambridge, UK). Cytoplasmic staining of FOXQ1 in at least 1% of cancer cells was classified as high. If no cancer cells were stained, specimens were classified as negative for FOXQ1 staining. The expression of FOXQ1 was evaluated in 10 fields of 100 cells each using high-power microscopy (400×).

### Genome-wide gene expression and *in silico* analyses

To identify *miR-216b-3p* target genes, a combination of genome-wide gene expression and *in silico* analyses was conducted as described previously [20, 28, 51, 52]. The microarray data were deposited into the Gene Omnibus database (GEO) repository under the accession number GSE82108. Next, we selected putative miRNA target genes using the TargetScanHuman (August, 2015 release, http://www.targetscan.org/vert_70) database.

### Construction of plasmid vectors and dual luciferase reporter assays

Wild-type sequences of the 3^׳^-UTR of *FOXQ1* containing the 2 *miR-216b-3p* target sites (positions 463-468 or 720-727) sequences or lacking a *miR-216b-3p* target site were inserted in the psiCHECK-2 vector (product ID: C8021; Promega, Madison, WI, USA). The procedure was described previously [20, 28, 51, 52].

### Identification of downstream targets regulated by *FOXQ1* in PDAC

Gene expression analyses of si-*FOXQ1*-transfected PANC-1 cells revealed molecular pathways and targets regulated by *FOXQ1* in PDAC cells. This method was described in detail in previous studies [15, 20]. Microarray results were deposited in the GEO database (accession number GSE77790).

### Statistical analysis

Relationships between expression values in 2 conditions or variables were analysed using the Mann-Whitney U test, and relationships between expression values in 3 conditions or variables were analysed using Bonferroni-adjusted Mann-Whitney U test. The correlation between the expression levels of *miR-216b-3p* and *FOXQ1* was evaluated using Spearman's rank test. Associations between different categories were assessed using Fisher’s exact test and the chi-squared test. Overall survival (OS) after surgery was gauged using Kaplan–Meier curves. Patients were divided into two groups based on *FOXQ1* expression, and differences in survival were estimated using the log-rank test. We used Expert StatView software (version 5.0 SAS Institute Inc., Cary, NC, USA) for these analyses.

## SUPPLEMENTARY MATERIALS FIGURES AND TABLES






