# Supplementary material for: The microRNA expression signature of pancreatic ductal adenocarcinoma by RNA sequencing: anti-tumour functions of the microRNA-216 cluster
Source: Oncotarget. 2017 Jul 26;8(41):70097–115. doi: 10.18632/oncotarget.19591 (PMC5642539; doi:10.18632/oncotarget.19591)
Supplement: Supplementary file 1 [file oncotarget-08-70097-s001.pdf]

The microRNA expression signature of pancreatic ductal adenocarcinoma by RNA sequencing: anti-tumour functions of the *microRNA-216* cluster

SUPPLEMENTARY MATERIALS

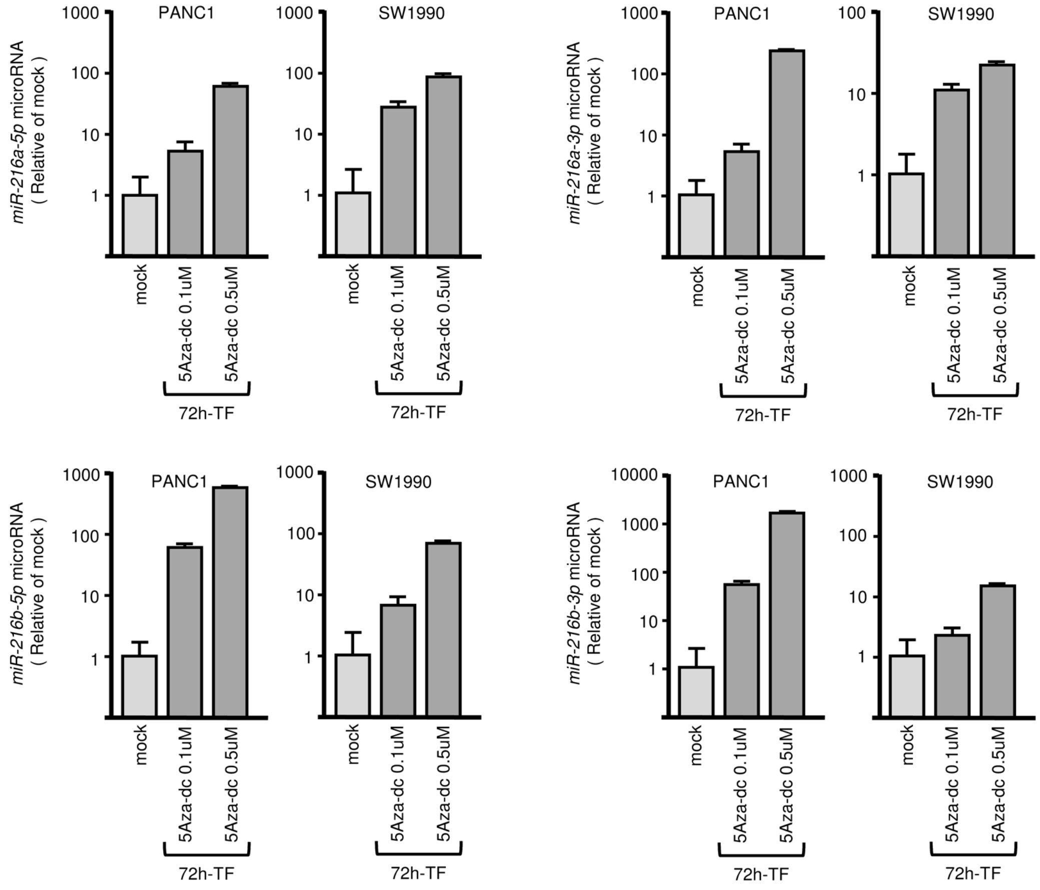

Supplementary Figure 1: Demethylation regulation induced high expression of clustered miRNAs in PDAC cell lines. Effect of demethylating agent [5-aza-2′-deoxycytidine (5-aza-dC)] (Wako, Osaka, Japan) 72 h treatment 0.1 µM and 0.5 µM on PDAC cell lines (PANC-1, SW1990). The expression level of *miR-216a-5p*, *miR-216a-3p*, *miR-216b-5p* and *miR-216b-3p* were normalized to *RNU48*.

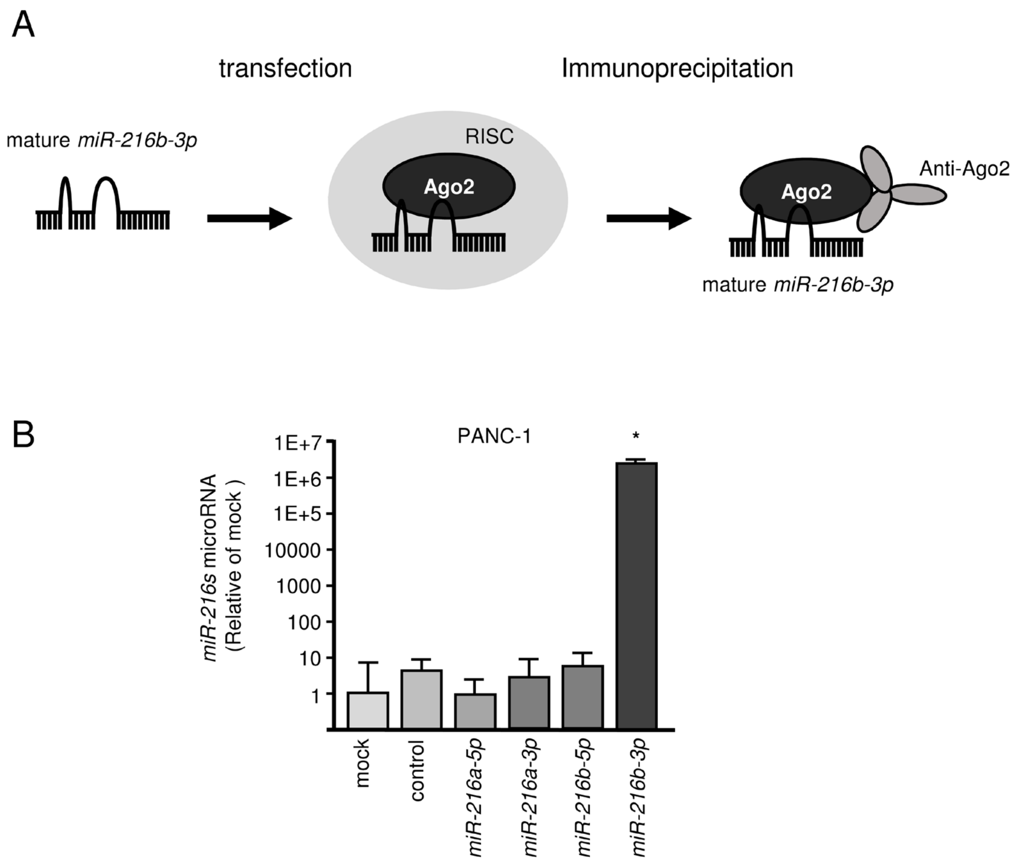

Supplementary Figure 2: *miR-216b-3p* was incorporated into RISC. **(A)** Schematic illustration of miRNA detection method. Isolation of RISC-incorporated miRNAs by Ago-2 immunoprecipitation. **(B)** Expression level of *miR-216b-3p* after transfection with *miR-216b-3p* following immunoprecipitation by Ago2 (*$P < 0.0001$).

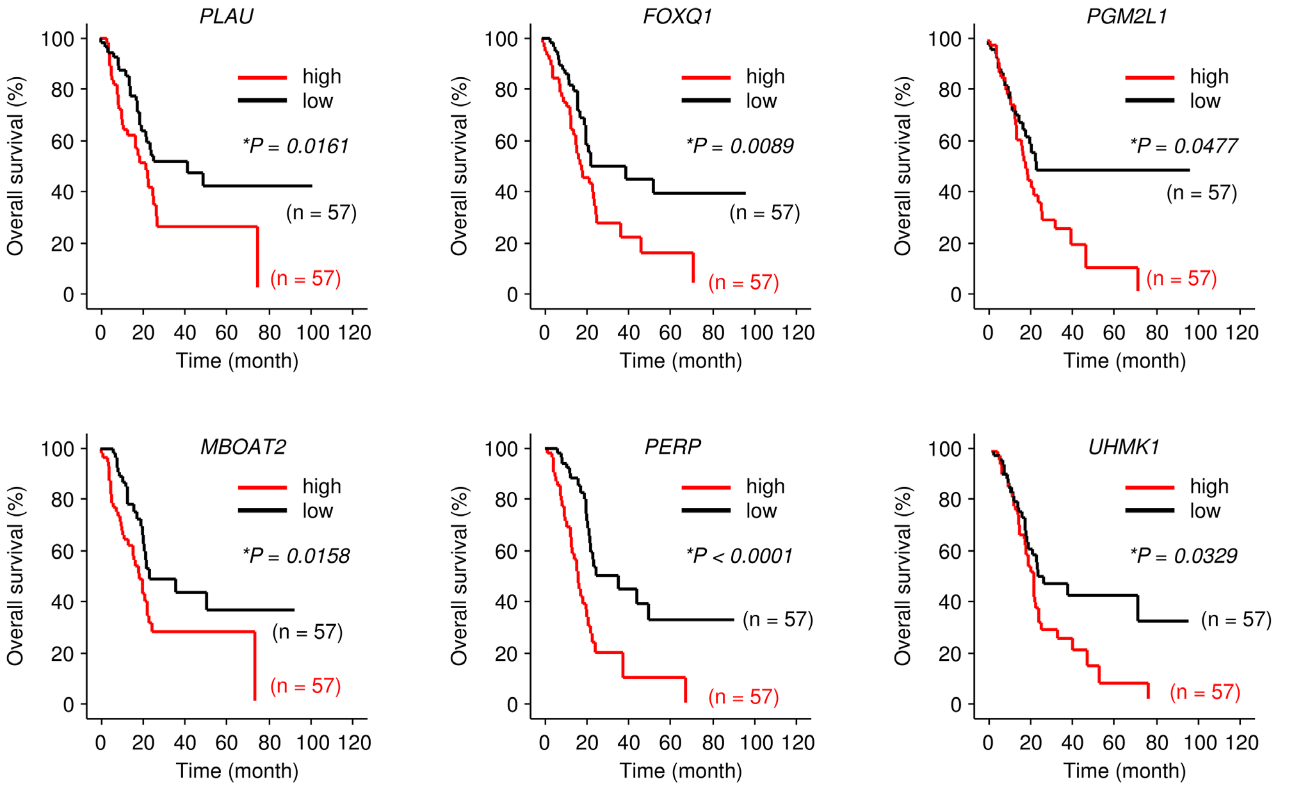

Supplementary Figure 3: TCGA database analysis of candidacy *miR-216b-3p* target genes. Kaplan–Meier plots overall survival with log-rank tests between those with high and low candidacy *miR-216-3p* target 6 genes expression in the PDAC TCGA database.

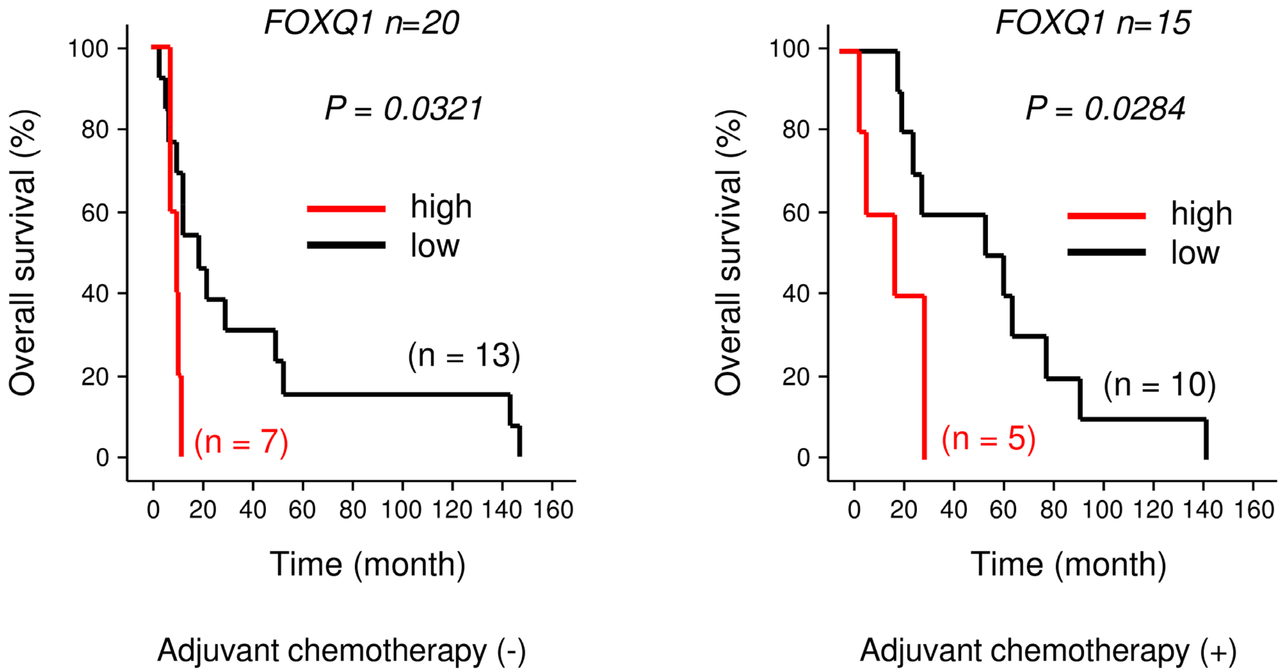

Supplementary Figure 4: Immunohistochemical staining of FOXQ1 in PDAC clinical specimens and association of FOXQ1 with overall survival. Kaplan–Meier survival curves for overall survival rates based on FOXQ1 expression. Patients were categorized into two groups, with or without adjuvant chemotherapy. *P*-values were calculated using the log-rank test.

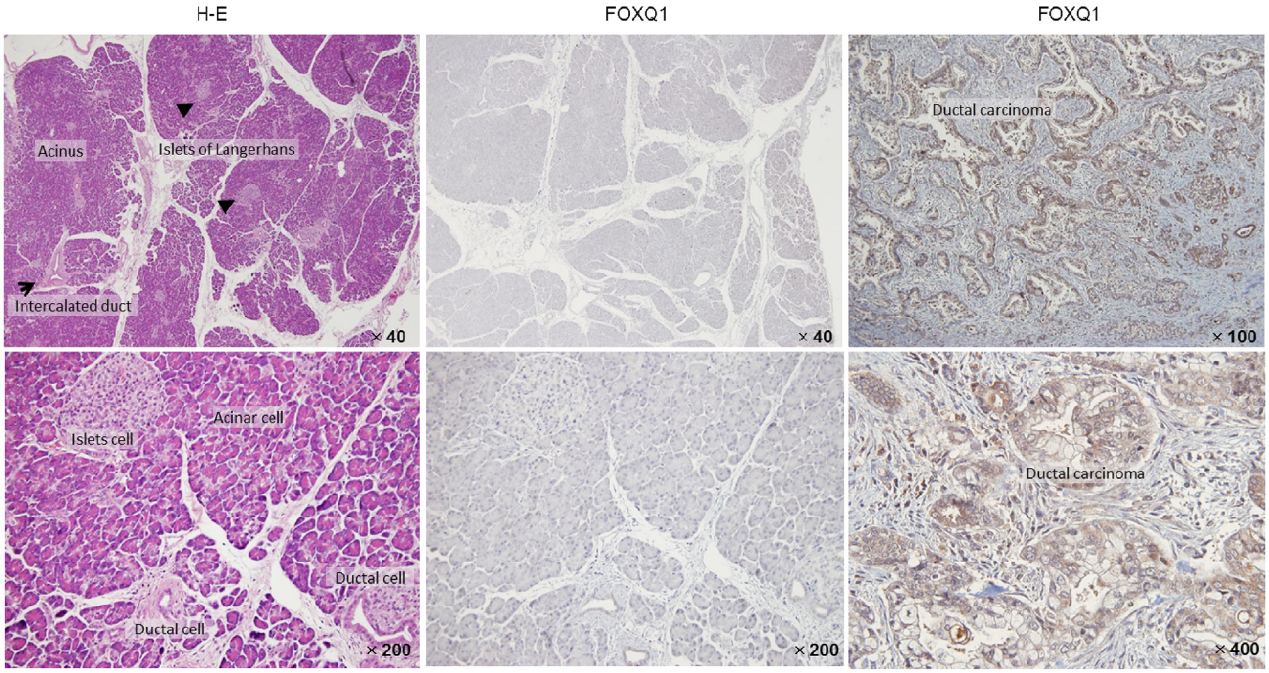

Supplementary Figure 5: Expression of FOXQ1 in PDAC clinical specimens. Immunohistochemical staining of FOXQ1 in PDAC clinical specimens. Over expression of FOXQ1 was observed in ductal adenocarcinoma (right side). In contrast, negative staining of FOXQ1 was observed in normal tissues (middle row).

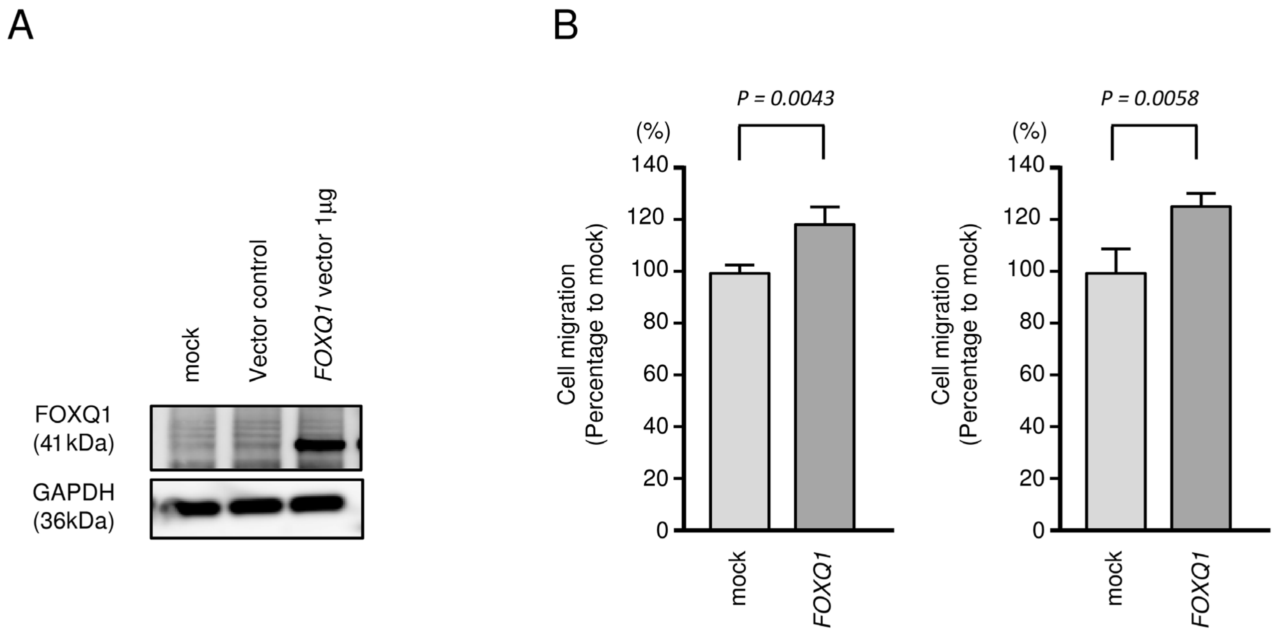

Supplementary Figure 6: Effects of overexpression of FOXQ1 in PANC-1 cells. For FOXQ1 overexpression studies, PANC-1 cell line was transfected with pCMV6-vecter of *FOXQ1* cDNA clone (Origene Technologies, TrueORF Gold, Accession No: NM_033260) using Lipofectamine 3000 (Life Technologies). **(A)** FOXQ1 protein overexpression in PANC-1 was evaluated by Western blot analyses 72 h after *FOXQ1* cDNA plasmid. *GAPDH* was used as a loading control. **(B)** Cell migration and invasion activities of 72 h transfection with 1.0 µg/well *FOXQ1* cDNA.

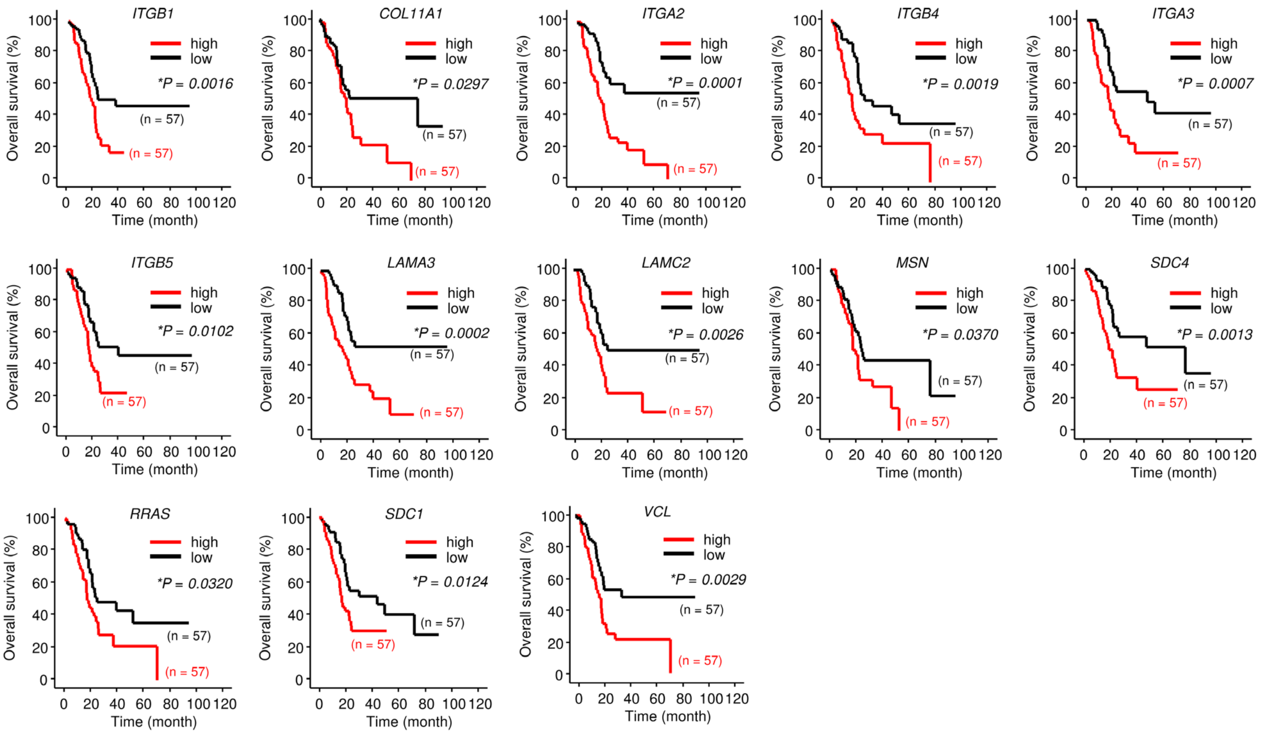

Supplementary Figure 7: TCGA database analysis of candidacy FOXQ1 downstream genes. Kaplan–Meier plots overall survival with log-rank tests between those with high and low candidacy *FOXQ1* target 13 genes expression in the PDAC TCGA database.

Supplementary Table 1: Downregulated miRNAs in PDAC by RNA sequencing

See supplementary File 1

Supplementary Table 2: Upregulated miRNAs in PDAC by RNA sequencing

See supplementary File 2

Supplementary Table 3A: Enriched KEGG pathways Top 3 downregulated by si-*FOXQ1*-transfected PDAC cell line (PANC-1)

Items	Items_Details	Support	Hyp	Hyp_c	Genes
Kegg:04512	ECM-receptor interaction	18	4.29E-13	6.39E-11	*SDC1, SDC4, COMP, LAMA3, ITGA3, ITGA2, HSPG2, ITGA5, COL1A1, TNC, LAMC2, ITGB1, COL5A1, ITGB4, ITGB8, LAMB1, ITGB5, COL11A1*
Kegg:04810	Regulation of actin cytoskeleton	27	3.07E-13	9.17E-11	*ARPC5, ACTN1, TIAM1, ITGB2, MYLK, F2R, ITGA3, MYH10, RAC2, ITGA2, MSN, CHRM3, GNA13, ITGA5, RRAS, VCL, PFN1, CD14, PDGFRB, PTK2, ITGB1, CFL1, ITGB4, ITGB8, MYL9, ITGB5, SSH1*
Kegg:04510	Focal adhesion	25	3.47E-12	3.45E-10	*ZYX, COMP, LAMA3, ACTN1, MYLK, ITGA3, RAC2, ITGA2, FLNA, ITGA5, VCL, COL1A1, TNC, PDGFRB, LAMC2, PTK2, ITGB1, FYN, COL5A1, ITGB4, ITGB8, LAMB1, MYL9, ITGB5, COL11A1*

Supplementary Table 3B: Downregulated genes in si-*FOXQ1*-trasufected PDAC cell line (PANC-1)

Entrez Gene ID	Gene symbol	Description	Expression in si-*FOXQ1* transfectanted PANC-1 FC (log2)	GEO GSE15471 FC (log2)	*TCGA-PAADOncoLnc 33:33 P-value
3696	*ITGB8*	integrin, beta 8	-1.633	1.574	0.0740
5159	*PDGFRB*	platelet-derived growth factor receptor, beta polypeptide	-1.293	1.799	0.4810
1277	*COL1A1*	collagen, type I, alpha 1	-1.180	4.251	0.5970
1289	*COL5A1*	collagen, type V, alpha 1	-1.149	3.496	0.3840
1131	*CHRM3*	cholinergic receptor, muscarinic 3	-1.128	1.532	0.6410
10398	*MYL9*	myosin, light chain 9, regulatory	-0.971	2.181	0.5160
3688	*ITGB1*	integrin, beta 1 (fibronectin receptor, beta polypeptide, antigen CD29 includes MDF2, MSK12)	-0.883	1.687	0.0016
4638	*MYLK*	myosin light chain kinase	-0.872	1.404	0.6250
1301	*COL11A1*	collagen, type XI, alpha 1	-0.815	4.416	0.0297
3673	*ITGA2*	integrin, alpha 2 (CD49B, alpha 2 subunit of VLA-2 receptor)	-0.770	2.619	0.0001
2149	*F2R*	coagulation factor II (thrombin) receptor	-0.706	2.294	0.8510
87	*ACTN1*	actinin, alpha 1	-0.607	1.499	0.1750
3691	*ITGB4*	integrin, beta 4	-0.602	1.232	0.0019
3371	*TNC*	tenascin C	-0.601	1.779	0.7040
3339	*HSPG2*	heparan sulfate proteoglycan 2	-0.521	1.080	0.9800
3678	*ITGA5*	integrin, alpha 5 (fibronectin receptor, alpha polypeptide)	-0.495	1.055	0.2530
10672	*GNA13*	guanine nucleotide binding protein (G protein), alpha 13	-0.468	1.109	0.3480
3675	*ITGA3*	integrin, alpha 3 (antigen CD49C, alpha 3 subunit of VLA-3 receptor)	-0.384	1.329	0.0007
3693	*ITGB5*	integrin, beta 5	-0.378	1.454	0.0102
7074	*TIAM1*	T-cell lymphoma invasion and metastasis 1	-0.354	1.192	0.1060
1311	*COMP*	cartilage oligomeric matrix protein	-0.348	3.572	0.6300
2316	*FLNA*	filamin A, alpha	-0.344	1.426	0.2080
2534	*FYN*	FYN proto-oncogene, Src family tyrosine kinase	-0.328	1.102	0.0176
929	*CD14*	CD14 molecule	-0.307	1.792	0.9390
3689	*ITGB2*	integrin, beta 2 (complement component 3 receptor 3 and 4 subunit)	-0.290	1.923	0.2720
3909	*LAMA3*	laminin, alpha 3	-0.236	2.262	0.0002
7791	*ZYX*	zyxin	-0.233	1.228	0.8170
10092	*ARPC5*	actin related protein 2/3 complex, subunit 5, 16kDa	-0.216	1.038	0.1870
5216	*PFN1*	profilin 1	-0.181	1.003	0.9830
1072	*CFL1*	cofilin 1 (non-muscle)	-0.168	1.174	0.0504
3918	*LAMC2*	laminin, gamma 2	-0.110	2.761	0.0026
4478	*MSN*	moesin	-0.099	1.740	0.0370
6385	*SDC4*	syndecan 4	-0.091	1.030	0.0013
5880	*RAC2*	ras-related C3 botulinum toxin substrate 2 (rho family, small GTP binding protein Rac2)	-0.080	1.323	0.1680
4628	*MYH10*	myosin, heavy chain 10, non-muscle	-0.055	1.142	0.0863
6237	*RRAS*	related RAS viral (r-ras) oncogene homolog	-0.045	1.147	0.0320
5747	*PTK2*	protein tyrosine kinase 2	-0.043	1.103	0.1650
6382	*SDC1*	syndecan 1	-0.022	1.384	0.0124
54434	*SSH1*	slingshot protein phosphatase 1	-0.007	1.266	0.3540
3912	*LAMB1*	laminin, beta 1	-0.005	1.678	0.7270
7414	*VCL*	vinculin	-0.005	1.050	0.0029

*Kaplan Meier survival analysis P<0.05 Poor Prognosis
