# Supplementary material for: The microRNA expression signature of pancreatic ductal adenocarcinoma by RNA sequencing: anti-tumour functions of the microRNA-216 cluster
Source: Oncotarget. 2017 Jul 26;8(41):70097–115. doi: 10.18632/oncotarget.19591 (PMC5642539; doi:10.18632/oncotarget.19591)
Supplement: Supplementary file 2 [file oncotarget-08-70097-s002.docx]

**Supplementary Table 2**: Upregulated miRNAs in PDAC

| **miRNA** | **locus** | **N1** | **N2** | **N3** | **N4** | **T1** | **T2** | **T3** | **T4** | **T5** | **T6** | **T7** | **logFC** | **PValue** | **FDR** |
| --- | --- | --- | --- | --- | --- | --- | --- | --- | --- | --- | --- | --- | --- | --- | --- |
| *hsa-miR-6887-3p* | chr19:35613648-35613668 | 0.678 | 0.000 | 0.000 | 0.267 | 1.379 | 0.317 | 2.305 | 1.249 | 4.741 | 11.020 | 17.431 | 4.1281 | 0.0007 | 0.1722 |
| *hsa-miR-4713-5p* | chr15:51534397-51534418 | 0.169 | 0.000 | 0.000 | 0.000 | 1.379 | 0.000 | 0.000 | 1.249 | 1.248 | 1.050 | 11.206 | 4.0365 | 0.0039 | 0.4750 |
| *hsa-miR-615-5p* | chr12:54427751-54427772 | 0.000 | 0.174 | 0.000 | 0.000 | 3.908 | 0.317 | 1.048 | 0.000 | 1.497 | 0.525 | 5.395 | 3.6140 | 0.0053 | 0.5030 |
| *hsa-miR-1248* | chr3:186504464-186504490 | 8.981 | 7.816 | 4.346 | 1.870 | 37.009 | 2.856 | 37.925 | 35.381 | 251.299 | 64.284 | 18.884 | 3.4987 | 0.0002 | 0.0956 |
| *hsa-miR-490-3p* | chr7:136587989-136588010 | 2.033 | 0.521 | 7.450 | 1.202 | 0.000 | 1.904 | 0.000 | 0.000 | 16.970 | 0.787 | 163.938 | 3.4687 | 0.0386 | 1.0000 |
| *hsa-miR-184* | chr15:79502182-79502203 | 12.201 | 24.490 | 97.674 | 41.408 | 1.609 | 52.205 | 6.076 | 6.244 | 0.749 | 3123.162 | 0.830 | 3.4566 | 0.0467 | 1.0000 |
| *hsa-miR-196a-5p* | chr17:46709894-46709915; | 44.229 | 9.379 | 1.449 | 5.477 | 96.315 | 0.793 | 293.761 | 8.741 | 72.120 | 7.872 | 397.394 | 3.2357 | 0.0103 | 0.6146 |
|  | chr12:54385546-54385567 |  |  |  |  |  |  |  |  |  |  |  |  |  |  |
| *hsa-miR-187-3p* | chr18:33484798-33484819 | 3.728 | 17.195 | 6.415 | 6.412 | 2.988 | 14.916 | 2.514 | 5.411 | 15.472 | 1.574 | 329.744 | 3.0532 | 0.0195 | 0.8676 |
| *hsa-miR-6516-3p* | chr17:75085551-75085572 | 1.864 | 4.169 | 6.622 | 1.736 | 10.344 | 2.380 | 1.886 | 37.878 | 1.248 | 88.161 | 51.464 | 3.0318 | 0.0046 | 0.5030 |
| *hsa-miR-490-5p* | chr7:136587952-136587971 | 1.017 | 0.174 | 2.690 | 1.469 | 0.000 | 2.222 | 0.000 | 0.416 | 5.740 | 0.000 | 56.652 | 2.9481 | 0.0457 | 1.0000 |
| *hsa-miR-3131* | chr2:219923447-219923469 | 0.678 | 0.000 | 0.000 | 0.000 | 0.000 | 0.317 | 1.676 | 2.081 | 6.738 | 3.673 | 0.415 | 2.9437 | 0.0112 | 0.6428 |
| *hsa-miR-4697-3p* | chr11:133768401-133768424 | 2.711 | 0.174 | 1.862 | 1.603 | 1.149 | 1.587 | 9.848 | 1.249 | 13.975 | 53.002 | 4.358 | 2.8983 | 0.0070 | 0.5030 |
| *hsa-miR-615-3p* | chr12:54427794-54427815 | 16.946 | 16.327 | 7.657 | 26.448 | 272.164 | 66.169 | 110.422 | 62.853 | 40.427 | 38.833 | 233.663 | 2.8592 | 0.0001 | 0.0901 |
| *hsa-miR-196b-5p* | chr7:27209147-27209168 | 18.471 | 3.300 | 6.001 | 2.805 | 31.492 | 5.078 | 112.727 | 24.559 | 31.444 | 78.978 | 57.275 | 2.7349 | 0.0009 | 0.1722 |
| *hsa-miR-187-5p* | chr18:33484834-33484855 | 0.000 | 0.174 | 0.000 | 0.000 | 0.000 | 0.159 | 0.000 | 0.000 | 0.000 | 0.000 | 5.810 | 2.7006 | 0.0610 | 1.0000 |
| *hsa-miR-6886-3p* | chr19:11224187-11224207 | 0.169 | 0.000 | 0.000 | 0.267 | 2.299 | 1.269 | 0.419 | 1.665 | 1.248 | 1.312 | 1.453 | 2.6795 | 0.0247 | 0.9200 |
| *hsa-miR-650* | chr22:23165285-23165305 | 0.169 | 0.347 | 0.000 | 0.668 | 0.460 | 0.000 | 0.629 | 2.914 | 8.734 | 2.886 | 2.075 | 2.6716 | 0.0138 | 0.7105 |
| *hsa-miR-492* | chr12:95228203-95228225 | 0.339 | 0.000 | 0.000 | 0.267 | 0.690 | 0.000 | 0.838 | 0.832 | 6.488 | 1.574 | 0.830 | 2.6592 | 0.0257 | 0.9200 |
| *hsa-miR-6744-5p* | chr11:1277840-1277858 | 0.169 | 0.000 | 0.207 | 0.401 | 3.448 | 0.952 | 1.886 | 0.000 | 1.747 | 3.936 | 1.038 | 2.6549 | 0.0169 | 0.8201 |
| *hsa-miR-6805-5p* | chr19:55899554-55899575 | 0.000 | 0.000 | 0.000 | 0.000 | 1.379 | 0.159 | 0.838 | 0.416 | 0.499 | 0.525 | 0.415 | 2.5534 | 0.0603 | 1.0000 |
| *hsa-miR-935* | chr19:54485616-54485638 | 13.048 | 2.953 | 2.690 | 1.870 | 5.747 | 7.299 | 88.631 | 5.827 | 17.469 | 63.759 | 12.866 | 2.5349 | 0.0059 | 0.5030 |
| *hsa-miR-4485* | chr11:10529824-10529843 | 17.454 | 17.022 | 17.590 | 62.913 | 21.608 | 13.646 | 15.505 | 156.093 | 22.959 | 98.132 | 707.838 | 2.5310 | 0.0192 | 0.8676 |
| *hsa-miR-196a-3p* | chr12:54385583-54385604 | 0.847 | 0.000 | 0.000 | 0.000 | 1.379 | 0.000 | 0.419 | 0.000 | 0.000 | 0.000 | 9.131 | 2.5198 | 0.0627 | 1.0000 |
| *hsa-miR-1* | chr18:19408976-19408997; | 0.000 | 0.000 | 0.000 | 0.000 | 0.000 | 0.000 | 0.000 | 0.000 | 0.499 | 0.000 | 3.320 | 2.4774 | 0.0697 | 1.0000 |
|  | chr20:61151558-61151579 |  |  |  |  |  |  |  |  |  |  |  |  |  |  |
| *hsa-miR-4768-5p* | chrX:17444012-17444033 | 0.000 | 0.174 | 0.000 | 0.134 | 1.149 | 0.159 | 0.629 | 0.832 | 1.248 | 1.574 | 1.038 | 2.4249 | 0.0567 | 1.0000 |
| *hsa-miR-6887-5p* | chr19:35613609-35613631 | 0.169 | 0.000 | 0.000 | 0.000 | 0.460 | 0.000 | 0.419 | 0.416 | 0.749 | 0.787 | 2.283 | 2.3878 | 0.0749 | 1.0000 |
| *hsa-miR-181b-3p* | chr1:198828016-198828036 | 0.000 | 0.000 | 0.000 | 0.000 | 0.230 | 0.635 | 0.838 | 1.665 | 0.000 | 0.262 | 0.000 | 2.3433 | 0.0827 | 1.0000 |
| *hsa-miR-4745-5p* | chr19:804941-804963 | 0.169 | 0.174 | 0.000 | 0.267 | 3.448 | 0.317 | 1.048 | 2.497 | 1.248 | 0.000 | 0.415 | 2.3230 | 0.0491 | 1.0000 |
| *hsa-miR-4724-5p* | chr17:29861911-29861933 | 0.169 | 0.000 | 0.000 | 0.000 | 0.690 | 0.159 | 0.210 | 0.832 | 1.497 | 1.312 | 0.000 | 2.2476 | 0.0950 | 1.0000 |
| *hsa-miR-6814-3p* | chr21:43166932-43166953 | 0.339 | 0.174 | 0.000 | 0.134 | 0.919 | 1.428 | 0.629 | 0.416 | 1.747 | 1.837 | 1.038 | 2.1536 | 0.0664 | 1.0000 |
| *hsa-miR-203b-3p* | chr14:104583765-104583787 | 0.508 | 0.347 | 0.000 | 0.267 | 0.000 | 0.635 | 1.048 | 1.249 | 3.244 | 3.936 | 1.038 | 2.1019 | 0.0518 | 1.0000 |
| *hsa-miR-143-5p* | chr5:148808507-148808528 | 8.473 | 17.716 | 19.452 | 32.859 | 69.880 | 74.579 | 86.536 | 55.777 | 46.666 | 21.516 | 202.328 | 2.0863 | 0.0019 | 0.3270 |
| *hsa-miR-6850-5p* | chr8:146017355-146017376 | 0.000 | 0.000 | 0.207 | 0.000 | 2.069 | 0.000 | 0.000 | 0.000 | 0.998 | 0.000 | 1.245 | 2.0852 | 0.1212 | 1.0000 |
| *hsa-miR-549a* | chr15:81134334-81134354 | 0.000 | 0.174 | 0.000 | 0.134 | 0.230 | 0.476 | 1.467 | 0.416 | 0.499 | 1.050 | 0.830 | 2.0632 | 0.1130 | 1.0000 |
| *hsa-miR-133a-3p* | chr18:19405673-19405694; | 100.319 | 100.046 | 231.356 | 191.010 | 162.747 | 753.091 | 138.709 | 154.011 | 227.092 | 68.220 | 2557.847 | 2.0598 | 0.0275 | 0.9691 |
|  | chr20:61162177-61162198 |  |  |  |  |  |  |  |  |  |  |  |  |  |  |
| *hsa-miR-3661* | chr5:133561468-133561489 | 0.169 | 0.000 | 0.000 | 0.534 | 1.609 | 1.587 | 0.838 | 1.249 | 1.497 | 0.262 | 0.830 | 2.0341 | 0.0800 | 1.0000 |
| *hsa-miR-4649-5p* | chr7:44150450-44150473 | 0.000 | 0.347 | 0.828 | 0.267 | 7.586 | 0.635 | 3.143 | 0.000 | 0.250 | 0.787 | 0.415 | 2.0240 | 0.0661 | 1.0000 |
| *hsa-miR-31-3p* | chr9:21512120-21512141 | 2.881 | 1.737 | 1.035 | 5.610 | 30.113 | 4.284 | 10.896 | 0.416 | 27.451 | 8.396 | 2.698 | 2.0170 | 0.0195 | 0.8676 |
| *hsa-miR-6862-5p* | chr16:28402346-28402367; | 0.339 | 0.347 | 0.000 | 0.534 | 5.057 | 2.222 | 0.000 | 0.000 | 0.000 | 0.000 | 3.735 | 2.0104 | 0.0770 | 1.0000 |
|  | chr16:28735578-28735599 |  |  |  |  |  |  |  |  |  |  |  |  |  |  |
| *hsa-miR-4741* | chr18:20513370-20513392 | 0.169 | 0.695 | 0.000 | 0.668 | 6.206 | 0.317 | 0.838 | 1.665 | 2.496 | 0.525 | 0.830 | 1.9526 | 0.0586 | 1.0000 |
| *hsa-miR-3607-5p* | chr5:85916325-85916346 | 0.508 | 1.042 | 0.207 | 0.000 | 0.690 | 0.000 | 0.000 | 1.665 | 0.499 | 7.609 | 2.698 | 1.9223 | 0.0753 | 1.0000 |
| *hsa-miR-3654* | chr7:132719620-132719638 | 1.017 | 0.000 | 0.000 | 0.401 | 0.460 | 0.793 | 0.838 | 2.497 | 1.497 | 3.936 | 1.660 | 1.9044 | 0.0645 | 1.0000 |
| *hsa-miR-10a-3p* | chr17:46657226-46657247 | 7.117 | 6.253 | 4.553 | 8.816 | 18.160 | 3.650 | 28.706 | 22.477 | 28.199 | 36.996 | 30.505 | 1.8869 | 0.0025 | 0.4048 |
| *hsa-miR-31-5p* | chr9:21512157-21512177 | 93.710 | 26.054 | 138.441 | 388.164 | 870.512 | 269.278 | 774.842 | 30.386 | 1493.567 | 501.154 | 431.011 | 1.8801 | 0.0257 | 0.9200 |
| *hsa-miR-1275* | chr6:33967795-33967811 | 19.488 | 6.253 | 5.587 | 5.610 | 14.022 | 40.622 | 3.772 | 17.066 | 7.986 | 5.510 | 124.925 | 1.8628 | 0.0417 | 1.0000 |
| *hsa-miR-937-5p* | chr8:144895180-144895199 | 0.169 | 0.000 | 0.000 | 0.000 | 0.460 | 0.000 | 1.886 | 0.000 | 0.250 | 0.525 | 0.208 | 1.8578 | 0.1734 | 1.0000 |
| *hsa-miR-92b-3p* | chr1:155165028-155165049 | 1851.839 | 790.117 | 3210.219 | 1816.332 | 2731.758 | 4443.016 | 2723.051 | 7253.939 | 3308.307 | 21998.309 | 5687.816 | 1.8417 | 0.0074 | 0.5045 |
| *hsa-miR-511-3p* | chr10:18134089-18134108 | 0.000 | 0.347 | 0.000 | 0.000 | 0.000 | 0.317 | 1.257 | 0.832 | 0.499 | 1.050 | 0.415 | 1.8360 | 0.1619 | 1.0000 |
| *hsa-miR-1269b* | chr17:12820630-12820651 | 0.000 | 0.000 | 0.000 | 0.000 | 0.000 | 0.317 | 1.886 | 0.000 | 0.000 | 0.000 | 0.000 | 1.8213 | 0.1674 | 1.0000 |
| *hsa-miR-4442* | chr3:25706366-25706382 | 0.847 | 0.000 | 0.207 | 0.668 | 0.919 | 3.808 | 0.210 | 3.746 | 0.250 | 3.411 | 0.830 | 1.8005 | 0.0707 | 1.0000 |
| *hsa-miR-4444* | chr2:178077496-178077513; | 1.695 | 1.042 | 0.828 | 0.267 | 0.460 | 0.317 | 1.676 | 4.162 | 4.991 | 8.921 | 3.735 | 1.7784 | 0.0422 | 1.0000 |
|  | chr3:75263669-75263686 |  |  |  |  |  |  |  |  |  |  |  |  |  |  |
| *hsa-miR-4496* | chr12:109029625-109029646 | 0.000 | 0.000 | 0.000 | 0.000 | 0.230 | 0.159 | 0.000 | 0.000 | 1.248 | 0.262 | 0.208 | 1.7725 | 0.1770 | 1.0000 |
| *hsa-miR-6877-3p* | chr9:135927422-135927442 | 0.000 | 0.000 | 0.000 | 0.000 | 0.000 | 0.793 | 0.000 | 0.000 | 0.250 | 1.050 | 0.000 | 1.7586 | 0.1792 | 1.0000 |
| *hsa-miR-4505* | chr14:74225452-74225469 | 0.000 | 0.000 | 0.000 | 0.000 | 0.000 | 0.635 | 0.210 | 1.249 | 0.000 | 0.000 | 0.000 | 1.7386 | 0.1817 | 1.0000 |
| *hsa-miR-6784-3p* | chr17:43191735-43191756 | 0.000 | 0.347 | 0.621 | 0.401 | 1.609 | 1.111 | 1.467 | 1.249 | 1.747 | 1.050 | 1.660 | 1.7239 | 0.0967 | 1.0000 |
| *hsa-miR-3609* | chr7:98479323-98479346 | 9.998 | 36.301 | 21.521 | 11.220 | 222.283 | 12.060 | 38.344 | 27.889 | 37.183 | 66.646 | 26.977 | 1.7200 | 0.0177 | 0.8431 |
| *hsa-miR-4443* | chr3:48238062-48238078 | 7.626 | 1.563 | 1.035 | 1.870 | 2.529 | 7.299 | 12.362 | 12.904 | 8.734 | 11.807 | 14.941 | 1.7189 | 0.0109 | 0.6377 |
| *hsa-miR-1254* | chr10:70519092-70519115; | 2.711 | 2.779 | 2.069 | 6.679 | 20.688 | 6.982 | 15.086 | 7.492 | 6.488 | 11.545 | 14.111 | 1.7090 | 0.0062 | 0.5030 |
|  | chr10:23682337-23682360 |  |  |  |  |  |  |  |  |  |  |  |  |  |  |
| *hsa-miR-1225-3p* | chr16:2140196-2140217 | 0.169 | 0.174 | 0.828 | 0.267 | 1.149 | 0.793 | 0.419 | 0.000 | 1.996 | 2.886 | 2.698 | 1.7082 | 0.1048 | 1.0000 |
| *hsa-miR-6720-3p* | chr6:1390559-1390580 | 0.000 | 0.000 | 0.207 | 0.401 | 0.690 | 0.793 | 0.419 | 0.416 | 0.000 | 0.787 | 2.283 | 1.7078 | 0.1663 | 1.0000 |
| *hsa-miR-550b-3p* | chr7:30329430-30329449; | 0.000 | 0.000 | 0.000 | 0.000 | 0.000 | 0.635 | 0.000 | 0.000 | 0.499 | 0.000 | 0.830 | 1.7035 | 0.1915 | 1.0000 |
|  | chr7:32772613-32772632 |  |  |  |  |  |  |  |  |  |  |  |  |  |  |
| *hsa-miR-513a-5p* | chrX:146295056-146295073; | 0.000 | 0.000 | 0.000 | 0.000 | 0.919 | 0.000 | 0.000 | 0.000 | 0.499 | 0.525 | 0.000 | 1.6911 | 0.1940 | 1.0000 |
|  | chrX:146307418-146307435 |  |  |  |  |  |  |  |  |  |  |  |  |  |  |
| *hsa-miR-3175* | chr15:93447638-93447659 | 0.000 | 0.000 | 0.000 | 0.000 | 0.460 | 0.476 | 0.000 | 0.832 | 0.000 | 0.000 | 0.208 | 1.6899 | 0.1927 | 1.0000 |
| *hsa-miR-6798-3p* | chr19:49513209-49513229 | 0.169 | 0.174 | 0.621 | 0.134 | 1.379 | 0.793 | 1.886 | 0.416 | 0.998 | 1.312 | 1.245 | 1.6840 | 0.1260 | 1.0000 |
| *hsa-miR-7151-3p* | chr10:69163109-69163129 | 0.000 | 0.000 | 0.000 | 0.134 | 0.230 | 1.111 | 0.000 | 0.416 | 0.499 | 0.262 | 0.208 | 1.6820 | 0.2175 | 1.0000 |
| *hsa-miR-509-3p* | chrX:146342068-146342089; | 3.559 | 7.816 | 1.862 | 3.206 | 28.274 | 1.904 | 11.315 | 14.985 | 5.989 | 25.976 | 1.868 | 1.6741 | 0.0250 | 0.9200 |
|  | chrX:146340293-146340314; |  |  |  |  |  |  |  |  |  |  |  |  |  |  |
|  | chrX:146341180-146341201 |  |  |  |  |  |  |  |  |  |  |  |  |  |  |
| *hsa-miR-518b* | chr19:54206041-54206062 | 0.000 | 0.000 | 0.207 | 0.000 | 1.379 | 0.000 | 0.629 | 0.000 | 0.499 | 0.525 | 0.000 | 1.6631 | 0.2209 | 1.0000 |
| *hsa-miR-4770* | chrX:6301948-6301965 | 0.000 | 0.000 | 0.000 | 0.000 | 0.000 | 1.111 | 0.000 | 0.832 | 0.000 | 0.000 | 0.000 | 1.6615 | 0.1978 | 1.0000 |
| *hsa-miR-6732-3p* | chr1:37945868-37945890 | 0.339 | 1.216 | 0.207 | 0.000 | 1.839 | 1.904 | 2.095 | 2.914 | 0.749 | 1.050 | 1.038 | 1.6561 | 0.0918 | 1.0000 |
| *hsa-miR-4435* | chr2:87929283-87929304; | 0.678 | 0.347 | 1.242 | 2.404 | 11.034 | 1.269 | 8.381 | 1.665 | 2.496 | 1.050 | 1.660 | 1.6357 | 0.0578 | 1.0000 |
|  | chr2:112078638-112078659 |  |  |  |  |  |  |  |  |  |  |  |  |  |  |
| *hsa-miR-612* | chr11:65211944-65211968 | 0.000 | 0.174 | 0.414 | 0.134 | 1.609 | 0.000 | 0.000 | 0.416 | 3.244 | 0.262 | 0.208 | 1.6291 | 0.1808 | 1.0000 |
| *hsa-miR-548az-5p* | chr8:120337430-120337451 | 0.339 | 0.347 | 0.000 | 0.000 | 0.230 | 0.159 | 0.838 | 0.000 | 0.250 | 0.000 | 3.735 | 1.6262 | 0.1949 | 1.0000 |
| *hsa-miR-6773-5p* | chr16:68267375-68267397 | 0.169 | 0.000 | 0.000 | 0.000 | 0.690 | 0.317 | 1.257 | 0.000 | 0.000 | 0.000 | 0.415 | 1.6081 | 0.2394 | 1.0000 |
| *hsa-miR-3150b-3p* | chr8:96085152-96085172 | 0.678 | 0.347 | 0.621 | 1.336 | 3.218 | 2.380 | 0.210 | 1.249 | 0.000 | 0.787 | 8.716 | 1.5794 | 0.1073 | 1.0000 |
| *hsa-miR-147b* | chr15:45725296-45725317 | 2.542 | 3.821 | 1.035 | 0.668 | 7.126 | 1.587 | 18.648 | 4.995 | 5.740 | 3.149 | 0.623 | 1.5698 | 0.0481 | 1.0000 |
| *hsa-miR-378i* | chr22:42319275-42319295 | 0.339 | 0.000 | 0.000 | 0.000 | 0.460 | 0.793 | 0.210 | 0.416 | 0.998 | 0.000 | 0.623 | 1.5676 | 0.2368 | 1.0000 |
| *hsa-miR-4519* | chr16:30886622-30886639 | 1.017 | 0.695 | 0.000 | 0.000 | 0.000 | 1.269 | 1.048 | 4.579 | 1.996 | 1.050 | 0.830 | 1.5651 | 0.1220 | 1.0000 |
| *hsa-miR-6891-5p* | chr6:31323074-31323092 | 0.169 | 0.000 | 0.000 | 0.000 | 1.839 | 0.000 | 0.000 | 0.000 | 0.749 | 0.000 | 0.000 | 1.5609 | 0.2525 | 1.0000 |
| *hsa-miR-5090* | chr7:102106199-102106221 | 0.169 | 0.174 | 0.207 | 0.134 | 1.149 | 1.111 | 0.419 | 0.832 | 0.998 | 0.787 | 0.000 | 1.5592 | 0.1977 | 1.0000 |
| *hsa-miR-92b-5p* | chr1:155164987-155165008 | 4.406 | 4.863 | 8.691 | 5.209 | 17.470 | 9.838 | 6.495 | 4.995 | 5.989 | 59.824 | 11.206 | 1.5523 | 0.0376 | 1.0000 |
| *hsa-miR-135b-3p* | chr1:205417451-205417472 | 1.186 | 1.042 | 0.000 | 0.134 | 2.529 | 0.159 | 3.562 | 1.249 | 2.246 | 2.624 | 1.038 | 1.5444 | 0.0992 | 1.0000 |
| *hsa-miR-3190-3p* | chr19:47730245-47730267 | 0.000 | 0.174 | 0.000 | 0.401 | 0.919 | 0.159 | 0.210 | 1.665 | 0.499 | 0.787 | 0.415 | 1.5438 | 0.2157 | 1.0000 |
| *hsa-miR-8072* | chr12:123849361-123849380 | 0.508 | 0.174 | 0.000 | 0.134 | 0.690 | 1.111 | 0.419 | 0.000 | 0.998 | 1.312 | 1.245 | 1.5404 | 0.1931 | 1.0000 |
| *hsa-miR-5001-5p* | chr2:233415240-233415263 | 0.339 | 0.347 | 0.000 | 0.267 | 1.379 | 1.587 | 0.419 | 0.832 | 0.499 | 0.787 | 1.038 | 1.5382 | 0.1783 | 1.0000 |
| *hsa-miR-133b* | chr6:52013786-52013807 | 2.033 | 0.695 | 3.104 | 1.469 | 0.460 | 14.122 | 1.257 | 2.497 | 2.496 | 0.787 | 15.979 | 1.5135 | 0.0966 | 1.0000 |
| *hsa-miR-3619-5p* | chr22:46486939-46486960 | 0.000 | 0.347 | 0.000 | 0.267 | 0.230 | 0.159 | 1.048 | 1.665 | 0.499 | 0.262 | 0.830 | 1.5131 | 0.2227 | 1.0000 |
| *hsa-miR-3687* | chr21:9826237-9826260 | 5.253 | 3.474 | 3.104 | 10.686 | 27.584 | 4.760 | 7.124 | 5.411 | 37.433 | 16.005 | 15.771 | 1.5106 | 0.0304 | 1.0000 |
| *hsa-miR-4769-3p* | chrX:47446875-47446896 | 0.000 | 0.000 | 0.000 | 0.000 | 0.230 | 0.317 | 0.000 | 0.832 | 0.000 | 0.262 | 0.000 | 1.5105 | 0.2335 | 1.0000 |
| *hsa-miR-320d* | chr13:41301964-41301982; | 14.065 | 7.990 | 6.208 | 12.957 | 17.930 | 12.853 | 16.343 | 15.817 | 12.977 | 47.229 | 68.273 | 1.5065 | 0.0257 | 0.9200 |
|  | chrX:140008337-140008355 |  |  |  |  |  |  |  |  |  |  |  |  |  |  |
| *hsa-miR-203a* | chr14:104583806-104583827 | 43.381 | 33.349 | 28.350 | 60.776 | 25.975 | 27.451 | 97.641 | 158.174 | 142.244 | 271.305 | 95.042 | 1.4987 | 0.0214 | 0.8791 |
| *hsa-miR-3610* | chr8:117886979-117886998 | 0.508 | 0.174 | 0.207 | 0.000 | 0.230 | 1.269 | 0.210 | 1.665 | 0.998 | 1.050 | 0.623 | 1.4924 | 0.1992 | 1.0000 |
| *hsa-miR-6723-5p* | chr1:567762-567783 | 6.609 | 16.153 | 10.140 | 18.700 | 18.619 | 9.679 | 15.086 | 12.487 | 9.733 | 81.077 | 80.101 | 1.4835 | 0.0534 | 1.0000 |
| *hsa-miR-577* | chr4:115577930-115577950 | 3.559 | 3.300 | 1.449 | 1.736 | 3.678 | 2.698 | 9.848 | 12.904 | 14.724 | 0.262 | 6.225 | 1.4774 | 0.0427 | 1.0000 |
| *hsa-miR-6754-5p* | chr11:71184554-71184575 | 0.000 | 0.000 | 0.000 | 0.134 | 1.379 | 0.000 | 0.419 | 0.416 | 0.000 | 0.000 | 0.000 | 1.4716 | 0.2801 | 1.0000 |
| *hsa-miR-320c* | chr18:19263520-19263539; | 34.061 | 59.749 | 37.249 | 79.610 | 110.567 | 72.040 | 81.717 | 47.036 | 50.160 | 167.664 | 389.093 | 1.4707 | 0.0334 | 1.0000 |
|  | chr18:21901680-21901699 |  |  |  |  |  |  |  |  |  |  |  |  |  |  |
| *hsa-miR-3187-5p* | chr19:813590-813612 | 0.000 | 0.000 | 0.000 | 0.000 | 0.230 | 0.317 | 0.000 | 0.000 | 0.000 | 0.787 | 0.208 | 1.4700 | 0.2454 | 1.0000 |
| *hsa-miR-194-5p* | chr1:220291548-220291569; | 1462.594 | 635.706 | 299.231 | 1096.772 | 994.641 | 793.713 | 1352.725 | 983.176 | 4346.193 | 4410.682 | 3288.512 | 1.4626 | 0.0311 | 1.0000 |
|  | chr11:64658876-64658897 |  |  |  |  |  |  |  |  |  |  |  |  |  |  |
| *hsa-miR-143-3p* | chr5:148808541-148808561 | 11020.037 | 11653.052 | 7897.142 | 14834.669 | 22315.181 | 24109.390 | 19800.582 | 50800.880 | 32550.531 | 12180.409 | 54515.380 | 1.4571 | 0.0043 | 0.5030 |
| *hsa-miR-3617-5p* | chr20:44333789-44333810 | 0.169 | 0.000 | 0.000 | 0.000 | 0.000 | 0.000 | 0.419 | 1.249 | 0.250 | 0.000 | 0.415 | 1.4436 | 0.2888 | 1.0000 |
| *hsa-miR-23a-5p* | chr19:13947444-13947465 | 2.372 | 12.679 | 2.069 | 28.050 | 131.025 | 16.820 | 17.810 | 19.564 | 12.977 | 16.005 | 11.621 | 1.4412 | 0.0794 | 1.0000 |
| *hsa-miR-4315* | chr17:43552774-43552791; | 0.000 | 0.000 | 0.000 | 0.000 | 0.000 | 0.000 | 0.000 | 0.000 | 1.497 | 0.000 | 0.000 | 1.4398 | 0.2526 | 1.0000 |
|  | chr17:62818193-62818210 |  |  |  |  |  |  |  |  |  |  |  |  |  |  |
| *hsa-miR-6881-5p* | chr15:74703747-74703768 | 0.000 | 0.000 | 0.000 | 0.000 | 0.919 | 0.159 | 0.210 | 0.000 | 0.000 | 0.000 | 0.208 | 1.4396 | 0.2528 | 1.0000 |
| *hsa-miR-7106-5p* | chr12:113596958-113596977 | 0.000 | 0.000 | 0.000 | 0.000 | 0.690 | 0.000 | 0.000 | 0.000 | 0.250 | 0.525 | 0.000 | 1.4218 | 0.2575 | 1.0000 |
| *hsa-miR-10a-5p* | chr17:46657266-46657288 | 11028.340 | 22133.868 | 9530.292 | 10010.262 | 36480.142 | 6468.079 | 48649.506 | 17396.216 | 36435.054 | 57365.891 | 21790.671 | 1.4046 | 0.0154 | 0.7610 |
| *hsa-miR-21-3p* | chr17:57918672-57918692 | 1171.126 | 1231.637 | 278.538 | 1213.782 | 2983.694 | 2039.820 | 3204.970 | 4388.910 | 2085.254 | 920.182 | 2512.193 | 1.3995 | 0.0064 | 0.5030 |
| *hsa-miR-5571-3p* | chr22:23228511-23228529 | 0.169 | 1.390 | 0.207 | 1.336 | 0.230 | 6.665 | 1.467 | 4.162 | 3.494 | 0.262 | 0.208 | 1.3920 | 0.1258 | 1.0000 |
| *hsa-miR-4791* | chr3:19356399-19356416 | 0.000 | 0.000 | 0.000 | 0.000 | 0.000 | 0.000 | 0.000 | 0.416 | 0.000 | 0.787 | 0.208 | 1.3885 | 0.2661 | 1.0000 |
| *hsa-miR-1228-5p* | chr12:57588287-57588307 | 0.000 | 0.000 | 0.207 | 0.134 | 0.230 | 0.159 | 0.210 | 0.000 | 1.248 | 0.262 | 0.830 | 1.3837 | 0.3008 | 1.0000 |
| *hsa-miR-6881-3p* | chr15:74703702-74703723 | 0.000 | 0.347 | 0.000 | 0.401 | 2.758 | 0.000 | 0.419 | 0.416 | 0.000 | 0.787 | 0.415 | 1.3834 | 0.2586 | 1.0000 |
| *hsa-miR-3176* | chr16:593336-593354 | 2.542 | 3.126 | 2.276 | 4.942 | 18.849 | 11.108 | 15.505 | 0.832 | 8.235 | 4.461 | 2.075 | 1.3803 | 0.0546 | 1.0000 |
| *hsa-miR-1184* | chrX:154115651-154115673; | 0.000 | 0.000 | 0.000 | 0.000 | 0.000 | 1.428 | 0.000 | 0.000 | 0.000 | 0.000 | 0.000 | 1.3761 | 0.2664 | 1.0000 |
|  | chrX:154612765-154612787; |  |  |  |  |  |  |  |  |  |  |  |  |  |  |
|  | chrX:154687238-154687260 |  |  |  |  |  |  |  |  |  |  |  |  |  |  |
| *hsa-miR-133a-5p* | chr18:19405710-19405731; | 0.000 | 0.000 | 0.000 | 0.000 | 0.000 | 0.000 | 0.000 | 0.000 | 0.000 | 0.525 | 0.830 | 1.3670 | 0.2736 | 1.0000 |
|  | chr20:61162140-61162161 |  |  |  |  |  |  |  |  |  |  |  |  |  |  |
| *hsa-miR-4758-5p* | chr20:60907590-60907612 | 0.000 | 0.000 | 0.000 | 0.000 | 0.230 | 0.000 | 0.210 | 0.416 | 0.000 | 0.525 | 0.000 | 1.3649 | 0.2717 | 1.0000 |
| *hsa-miR-4728-3p* | chr17:37882790-37882814 | 3.728 | 4.690 | 10.140 | 5.744 | 20.688 | 11.108 | 11.524 | 4.579 | 9.233 | 26.763 | 22.412 | 1.3627 | 0.0287 | 0.9691 |
| *hsa-miR-513c-5p* | chrX:146271271-146271292 | 0.000 | 0.174 | 0.000 | 0.000 | 0.690 | 0.000 | 0.419 | 0.000 | 0.499 | 0.525 | 0.000 | 1.3599 | 0.3193 | 1.0000 |
| *hsa-miR-1284* | chr3:71591190-71591211 | 0.169 | 0.000 | 0.000 | 0.000 | 0.460 | 0.793 | 0.210 | 0.000 | 0.250 | 0.000 | 0.415 | 1.3523 | 0.3202 | 1.0000 |
| *hsa-miR-7706* | chr15:85923870-85923893 | 21.352 | 46.375 | 46.768 | 55.299 | 288.255 | 69.343 | 74.802 | 77.006 | 69.874 | 89.735 | 88.817 | 1.3503 | 0.0124 | 0.6808 |
| *hsa-miR-4677-5p* | chr1:243509490-243509511 | 0.000 | 0.174 | 0.000 | 0.000 | 0.690 | 0.159 | 0.000 | 0.000 | 0.749 | 0.525 | 0.000 | 1.3503 | 0.3221 | 1.0000 |
| *hsa-miR-6893-3p* | chr8:145660934-145660955 | 0.000 | 0.000 | 0.000 | 0.000 | 0.460 | 0.159 | 0.000 | 0.000 | 0.250 | 0.262 | 0.208 | 1.3414 | 0.2785 | 1.0000 |
| *hsa-miR-6819-5p* | chr22:36682927-36682948 | 0.000 | 0.000 | 0.000 | 0.134 | 0.230 | 0.476 | 0.210 | 0.832 | 0.000 | 0.000 | 0.208 | 1.3357 | 0.3236 | 1.0000 |
| *hsa-miR-877-5p* | chr6:30552109-30552128 | 23.555 | 32.480 | 31.661 | 40.606 | 284.347 | 81.561 | 59.087 | 42.457 | 36.435 | 46.180 | 33.410 | 1.3318 | 0.0320 | 1.0000 |
| *hsa-miR-4658* | chr7:99754234-99754256 | 0.339 | 0.000 | 0.000 | 0.000 | 1.609 | 0.000 | 0.419 | 0.000 | 0.250 | 0.525 | 0.000 | 1.3258 | 0.3214 | 1.0000 |
| *hsa-miR-1282* | chr15:44085930-44085949 | 0.169 | 0.000 | 0.000 | 0.000 | 0.690 | 0.000 | 0.000 | 0.416 | 0.000 | 0.525 | 0.415 | 1.3199 | 0.3326 | 1.0000 |
| *hsa-miR-190a-5p* | chr15:63116170-63116191 | 1.186 | 2.258 | 2.069 | 2.939 | 2.758 | 0.635 | 3.562 | 6.660 | 5.740 | 9.446 | 8.093 | 1.3149 | 0.0666 | 1.0000 |
| *hsa-miR-4758-3p* | chr20:60907546-60907568 | 1.017 | 0.347 | 1.449 | 0.401 | 3.218 | 0.635 | 5.238 | 2.914 | 0.998 | 1.050 | 1.245 | 1.3110 | 0.1370 | 1.0000 |
| *hsa-miR-145-3p* | chr5:148810262-148810283 | 21.352 | 32.306 | 29.178 | 36.332 | 47.353 | 63.630 | 66.840 | 93.656 | 87.842 | 35.422 | 114.549 | 1.3084 | 0.0058 | 0.5030 |
| *hsa-miR-514a-3p* | chrX:146360784-146360804; | 0.000 | 0.000 | 0.000 | 0.000 | 0.000 | 0.000 | 1.257 | 0.000 | 0.000 | 0.000 | 0.000 | 1.2920 | 0.2925 | 1.0000 |
|  | chrX:146363475-146363495; |  |  |  |  |  |  |  |  |  |  |  |  |  |  |
|  | chrX:146366173-146366193 |  |  |  |  |  |  |  |  |  |  |  |  |  |  |
| *hsa-miR-6886-5p* | chr19:11224155-11224175 | 0.000 | 0.347 | 0.000 | 0.134 | 1.609 | 0.635 | 0.000 | 0.832 | 0.000 | 0.262 | 0.000 | 1.2822 | 0.3233 | 1.0000 |
| *hsa-miR-548h-5p* | chr14:64561802-64561823; | 0.000 | 0.000 | 0.000 | 0.000 | 0.000 | 0.000 | 0.000 | 0.000 | 1.248 | 0.000 | 0.000 | 1.2791 | 0.2952 | 1.0000 |
|  | chr16:11400348-11400369; |  |  |  |  |  |  |  |  |  |  |  |  |  |  |
|  | chr17:13446914-13446935; |  |  |  |  |  |  |  |  |  |  |  |  |  |  |
|  | chr8:26906443-26906464; |  |  |  |  |  |  |  |  |  |  |  |  |  |  |
|  | chr6:132113314-132113335 |  |  |  |  |  |  |  |  |  |  |  |  |  |  |
| *hsa-miR-3652* | chr12:104324203-104324220 | 0.508 | 0.174 | 0.414 | 0.401 | 1.379 | 0.000 | 0.838 | 1.249 | 1.497 | 1.574 | 1.038 | 1.2770 | 0.2284 | 1.0000 |
| *hsa-miR-4761-3p* | chr22:19951337-19951357 | 0.000 | 0.000 | 0.000 | 0.000 | 0.230 | 0.476 | 0.000 | 0.000 | 0.000 | 0.525 | 0.000 | 1.2634 | 0.2990 | 1.0000 |
| *hsa-miR-4648* | chr7:2566708-2566727 | 0.000 | 0.000 | 0.207 | 0.267 | 0.230 | 0.476 | 0.210 | 0.000 | 0.499 | 0.525 | 1.245 | 1.2623 | 0.3347 | 1.0000 |
| *hsa-miR-548a-3p* | chr6:18572075-18572096; | 0.000 | 0.000 | 0.000 | 0.000 | 0.000 | 0.000 | 0.000 | 1.249 | 0.000 | 0.000 | 0.000 | 1.2618 | 0.2975 | 1.0000 |
|  | chr6:135560358-135560379; |  |  |  |  |  |  |  |  |  |  |  |  |  |  |
|  | chr8:105496612-105496633 |  |  |  |  |  |  |  |  |  |  |  |  |  |  |
| *hsa-miR-4654* | chr1:162126906-162126927 | 0.169 | 0.174 | 0.414 | 0.134 | 0.460 | 0.159 | 0.210 | 1.249 | 0.250 | 1.574 | 1.038 | 1.2607 | 0.2892 | 1.0000 |
| *hsa-miR-4526* | chr18:13611166-13611187 | 0.169 | 0.174 | 0.000 | 0.267 | 0.690 | 1.904 | 0.000 | 0.000 | 0.250 | 0.787 | 0.208 | 1.2592 | 0.3191 | 1.0000 |
| *hsa-miR-6788-3p* | chr18:10759582-10759603 | 0.339 | 0.000 | 0.207 | 0.267 | 0.919 | 0.317 | 0.210 | 0.832 | 1.497 | 0.262 | 0.623 | 1.2583 | 0.2958 | 1.0000 |
| *hsa-miR-6780b-5p* | chr6:43402285-43402307 | 0.000 | 0.000 | 0.207 | 0.000 | 0.230 | 0.635 | 0.000 | 0.832 | 0.000 | 0.000 | 0.415 | 1.2559 | 0.3532 | 1.0000 |
| *hsa-miR-627-3p* | chr15:42491791-42491810 | 0.169 | 0.174 | 0.000 | 0.267 | 0.460 | 0.635 | 0.629 | 0.416 | 0.998 | 0.000 | 0.623 | 1.2511 | 0.3241 | 1.0000 |
| *hsa-miR-4707-5p* | chr14:23426207-23426229 | 0.000 | 0.000 | 0.000 | 0.000 | 0.230 | 0.000 | 0.000 | 0.000 | 0.499 | 0.262 | 0.208 | 1.2502 | 0.3040 | 1.0000 |
| *hsa-miR-4708-5p* | chr14:65801873-65801893 | 0.339 | 0.000 | 0.000 | 0.000 | 0.230 | 0.000 | 0.210 | 0.000 | 0.998 | 0.525 | 0.623 | 1.2477 | 0.3529 | 1.0000 |
| *hsa-miR-4479* | chr9:139781229-139781250 | 0.000 | 0.347 | 0.000 | 0.267 | 1.149 | 0.476 | 0.838 | 0.000 | 0.000 | 1.050 | 0.208 | 1.2460 | 0.3271 | 1.0000 |
| *hsa-miR-636* | chr17:74732548-74732570 | 0.508 | 1.563 | 1.035 | 0.668 | 1.379 | 2.698 | 2.305 | 2.914 | 0.998 | 3.149 | 3.113 | 1.2369 | 0.1344 | 1.0000 |
| *hsa-miR-320e* | chr19:47212551-47212568 | 0.678 | 0.695 | 0.414 | 0.134 | 0.919 | 1.428 | 1.048 | 0.832 | 0.499 | 2.361 | 1.868 | 1.2336 | 0.2183 | 1.0000 |
| *hsa-miR-6855-5p* | chr9:132631889-132631913 | 0.169 | 0.000 | 0.000 | 0.267 | 1.149 | 0.476 | 0.000 | 0.000 | 0.250 | 0.262 | 0.830 | 1.2299 | 0.3503 | 1.0000 |
| *hsa-miR-4783-3p* | chr2:128181122-128181144 | 0.169 | 0.347 | 0.000 | 0.134 | 0.460 | 3.015 | 0.210 | 0.000 | 0.000 | 0.262 | 0.000 | 1.2281 | 0.3266 | 1.0000 |
| *hsa-miR-6836-3p* | chr7:2297150-2297170 | 0.000 | 0.521 | 0.000 | 0.000 | 0.690 | 0.476 | 0.210 | 0.416 | 0.499 | 0.787 | 0.208 | 1.2275 | 0.3440 | 1.0000 |
| *hsa-miR-6840-5p* | chr7:99954279-99954302 | 0.000 | 0.000 | 0.207 | 0.000 | 1.609 | 0.159 | 0.000 | 0.000 | 0.000 | 0.262 | 0.000 | 1.2275 | 0.3656 | 1.0000 |
| *hsa-miR-6758-3p* | chr12:57906514-57906533 | 0.000 | 0.000 | 0.000 | 0.000 | 0.230 | 0.000 | 0.210 | 0.000 | 0.250 | 0.262 | 0.208 | 1.2232 | 0.3118 | 1.0000 |
| *hsa-miR-3116* | chr1:62544502-62544523; | 0.000 | 0.347 | 0.000 | 0.267 | 0.000 | 1.269 | 0.838 | 1.665 | 0.000 | 0.000 | 0.000 | 1.2229 | 0.3325 | 1.0000 |
|  | chr1:62544466-62544487 |  |  |  |  |  |  |  |  |  |  |  |  |  |  |
| *hsa-miR-1247-5p* | chr14:102026699-102026720 | 176.575 | 87.366 | 344.551 | 229.346 | 159.759 | 707.233 | 318.905 | 323.840 | 455.432 | 1296.966 | 258.150 | 1.2157 | 0.0474 | 1.0000 |
| *hsa-miR-4518* | chr16:30515289-30515314 | 0.000 | 0.000 | 0.000 | 0.000 | 0.000 | 0.000 | 0.000 | 0.416 | 0.749 | 0.000 | 0.000 | 1.2155 | 0.3123 | 1.0000 |
| *hsa-miR-323b-5p* | chr14:101522570-101522592 | 0.000 | 0.174 | 0.000 | 0.000 | 0.000 | 1.904 | 0.000 | 0.000 | 0.000 | 0.000 | 0.000 | 1.2140 | 0.3678 | 1.0000 |
| *hsa-miR-4753-5p* | chr1:235353401-235353422 | 0.000 | 0.000 | 0.000 | 0.000 | 0.690 | 0.000 | 0.210 | 0.000 | 0.250 | 0.000 | 0.000 | 1.2104 | 0.3147 | 1.0000 |
| *hsa-miR-1229-3p* | chr5:179225278-179225300 | 1.864 | 2.432 | 6.001 | 2.404 | 13.332 | 5.712 | 5.867 | 7.909 | 5.740 | 7.084 | 7.056 | 1.2101 | 0.0443 | 1.0000 |
| *hsa-miR-1914-5p* | chr20:62572864-62572885 | 0.169 | 0.347 | 0.000 | 0.134 | 0.460 | 1.269 | 0.419 | 0.416 | 0.499 | 0.525 | 0.208 | 1.2047 | 0.3388 | 1.0000 |
| *hsa-miR-210-5p* | chr11:568150-568171 | 13.048 | 15.458 | 16.348 | 6.011 | 23.676 | 17.296 | 22.210 | 55.777 | 12.478 | 64.547 | 10.168 | 1.2036 | 0.0388 | 1.0000 |
| *hsa-miR-6736-5p* | chr1:145584428-145584448 | 0.169 | 0.000 | 0.000 | 0.000 | 0.000 | 0.000 | 0.000 | 0.832 | 0.000 | 0.787 | 0.208 | 1.2006 | 0.3755 | 1.0000 |
| *hsa-miR-6811-5p* | chr2:238419575-238419596 | 0.000 | 0.174 | 0.000 | 0.000 | 0.230 | 0.000 | 0.419 | 0.416 | 0.499 | 0.262 | 0.000 | 1.1978 | 0.3777 | 1.0000 |
| *hsa-miR-4701-3p* | chr12:49165763-49165782 | 0.000 | 0.000 | 0.000 | 0.000 | 0.460 | 0.000 | 0.000 | 0.416 | 0.000 | 0.262 | 0.000 | 1.1978 | 0.3176 | 1.0000 |
| *hsa-miR-508-3p* | chrX:146318463-146318485 | 0.339 | 0.868 | 0.207 | 0.134 | 0.919 | 1.269 | 0.419 | 0.832 | 0.250 | 3.149 | 0.415 | 1.1937 | 0.2641 | 1.0000 |
| *hsa-miR-548l* | chr11:94199711-94199732 | 0.169 | 0.000 | 0.000 | 0.000 | 0.000 | 0.000 | 0.629 | 0.000 | 0.000 | 0.525 | 0.623 | 1.1929 | 0.3816 | 1.0000 |
| *hsa-miR-7113-5p* | chr11:67800332-67800352 | 0.169 | 0.000 | 0.000 | 0.000 | 0.460 | 0.000 | 0.210 | 0.416 | 0.250 | 0.262 | 0.208 | 1.1919 | 0.3793 | 1.0000 |
| *hsa-miR-3918* | chr6:159185747-159185767 | 0.000 | 0.000 | 0.000 | 0.134 | 0.460 | 0.159 | 0.000 | 0.416 | 0.000 | 0.000 | 0.623 | 1.1902 | 0.3780 | 1.0000 |
| *hsa-miR-4529-3p* | chr18:53146501-53146521 | 0.169 | 0.000 | 0.000 | 0.000 | 0.000 | 0.159 | 0.210 | 0.416 | 0.499 | 0.525 | 0.000 | 1.1902 | 0.3794 | 1.0000 |
| *hsa-miR-1273c* | chr6:155174503-155174524 | 0.000 | 0.000 | 0.000 | 0.000 | 0.000 | 0.159 | 0.000 | 0.000 | 0.749 | 0.000 | 0.208 | 1.1867 | 0.3217 | 1.0000 |
| *hsa-miR-6858-5p* | chrX:153678668-153678689 | 0.169 | 0.174 | 0.000 | 0.401 | 1.149 | 0.159 | 0.419 | 0.000 | 0.000 | 2.099 | 0.208 | 1.1835 | 0.3410 | 1.0000 |
| *hsa-miR-3648* | chr21:9825859-9825879 | 5.762 | 5.905 | 1.242 | 4.541 | 17.700 | 9.679 | 6.495 | 3.746 | 15.722 | 4.723 | 10.583 | 1.1747 | 0.0560 | 1.0000 |
| *hsa-miR-3127-5p* | chr2:97464025-97464047 | 2.542 | 3.474 | 2.690 | 4.541 | 7.356 | 4.126 | 1.886 | 5.411 | 4.991 | 14.169 | 13.281 | 1.1739 | 0.0799 | 1.0000 |
| *hsa-miR-3934-5p* | chr6:33665929-33665950 | 3.220 | 3.821 | 1.862 | 5.209 | 6.206 | 4.284 | 5.238 | 9.157 | 7.487 | 13.382 | 9.961 | 1.1708 | 0.0524 | 1.0000 |
| *hsa-miR-4642* | chr6:44403387-44403408 | 0.169 | 0.000 | 0.000 | 0.134 | 0.690 | 1.111 | 0.000 | 0.000 | 0.250 | 0.262 | 0.000 | 1.1628 | 0.3855 | 1.0000 |
| *hsa-miR-5010-5p* | chr17:40666226-40666247 | 0.000 | 0.521 | 0.000 | 0.534 | 1.839 | 0.317 | 0.629 | 0.000 | 0.749 | 0.787 | 0.830 | 1.1615 | 0.3178 | 1.0000 |
| *hsa-miR-598-5p* | chr8:10892767-10892789 | 0.000 | 0.000 | 0.000 | 0.000 | 0.000 | 0.000 | 0.419 | 0.416 | 0.250 | 0.000 | 0.000 | 1.1599 | 0.3288 | 1.0000 |
| *hsa-miR-1293* | chr12:50627965-50627986 | 0.000 | 0.000 | 0.000 | 0.267 | 0.230 | 0.159 | 0.000 | 0.000 | 1.248 | 0.262 | 0.208 | 1.1483 | 0.3955 | 1.0000 |
| *hsa-miR-1276* | chr15:86313779-86313798 | 0.847 | 0.521 | 0.207 | 1.202 | 2.758 | 1.111 | 1.676 | 1.249 | 0.998 | 2.361 | 1.660 | 1.1473 | 0.2072 | 1.0000 |
| *hsa-miR-892c-3p* | chrX:145074278-145074299 | 0.000 | 0.000 | 0.000 | 0.000 | 0.000 | 0.000 | 0.210 | 0.000 | 0.000 | 0.000 | 0.830 | 1.1447 | 0.3364 | 1.0000 |
| *hsa-miR-3655* | chr5:140027429-140027449 | 0.169 | 0.347 | 0.828 | 0.000 | 0.000 | 0.793 | 0.419 | 0.832 | 0.749 | 2.361 | 1.038 | 1.1409 | 0.3025 | 1.0000 |
| *hsa-miR-135b-5p* | chr1:205417489-205417511 | 8.473 | 19.974 | 2.483 | 3.473 | 10.114 | 2.063 | 21.582 | 10.406 | 21.711 | 47.754 | 5.810 | 1.1375 | 0.1255 | 1.0000 |
| *hsa-miR-6795-5p* | chr19:15290133-15290156 | 0.169 | 0.174 | 0.000 | 0.267 | 0.919 | 0.317 | 0.419 | 0.832 | 0.250 | 0.262 | 0.415 | 1.1349 | 0.3732 | 1.0000 |
| *hsa-miR-6516-5p* | chr17:75085510-75085531 | 0.508 | 0.521 | 0.207 | 0.534 | 0.690 | 1.269 | 1.048 | 1.249 | 0.499 | 0.525 | 2.490 | 1.1309 | 0.2733 | 1.0000 |
| *hsa-miR-7108-3p* | chr19:2434912-2434931 | 0.000 | 0.000 | 0.414 | 0.000 | 0.230 | 1.745 | 0.000 | 0.000 | 0.499 | 0.000 | 0.208 | 1.1289 | 0.3913 | 1.0000 |
| *hsa-miR-6895-3p* | chrX:53224593-53224614 | 0.339 | 0.521 | 0.828 | 0.267 | 1.149 | 1.587 | 0.838 | 1.249 | 0.250 | 2.624 | 0.830 | 1.1272 | 0.2616 | 1.0000 |
| *hsa-miR-6877-5p* | chr9:135927388-135927409 | 0.169 | 0.347 | 0.207 | 0.267 | 1.379 | 0.952 | 0.210 | 0.416 | 0.499 | 0.525 | 0.830 | 1.1222 | 0.3387 | 1.0000 |
| *hsa-miR-6777-5p* | chr17:17716832-17716854 | 0.000 | 0.174 | 0.000 | 0.134 | 0.230 | 0.793 | 0.000 | 0.416 | 0.250 | 0.525 | 0.000 | 1.1160 | 0.4056 | 1.0000 |
| *hsa-miR-5196-3p* | chr19:35836484-35836504 | 0.339 | 0.000 | 0.414 | 0.134 | 0.690 | 1.428 | 0.000 | 0.832 | 0.749 | 0.787 | 0.000 | 1.1154 | 0.3496 | 1.0000 |
| *hsa-miR-6805-3p* | chr19:55899588-55899610 | 0.000 | 0.000 | 0.207 | 0.000 | 0.230 | 0.000 | 0.000 | 0.000 | 0.250 | 1.312 | 0.000 | 1.1136 | 0.4122 | 1.0000 |
| *hsa-miR-4473* | chr9:20411159-20411180 | 0.339 | 0.347 | 0.000 | 0.000 | 0.460 | 0.793 | 0.210 | 0.416 | 0.749 | 0.787 | 0.208 | 1.1131 | 0.3765 | 1.0000 |
| *hsa-miR-450b-5p* | chrX:133674261-133674282 | 1.356 | 3.647 | 0.207 | 0.668 | 1.149 | 1.745 | 6.076 | 9.990 | 2.995 | 1.312 | 0.000 | 1.1042 | 0.1807 | 1.0000 |
| *hsa-miR-708-3p* | chr11:79113076-79113097 | 1.525 | 3.995 | 1.242 | 1.202 | 4.138 | 3.491 | 5.238 | 10.822 | 0.499 | 6.560 | 0.415 | 1.1033 | 0.1349 | 1.0000 |
| *hsa-miR-6895-5p* | chrX:53224645-53224665 | 0.000 | 0.174 | 0.414 | 0.000 | 0.460 | 0.159 | 0.838 | 0.000 | 1.248 | 0.262 | 0.208 | 1.0961 | 0.3946 | 1.0000 |
| *hsa-miR-142-5p* | chr17:56408644-56408664 | 147.598 | 269.741 | 83.396 | 85.487 | 157.460 | 446.999 | 87.793 | 1366.539 | 237.074 | 70.581 | 39.013 | 1.0946 | 0.1354 | 1.0000 |
| *hsa-miR-4488* | chr11:61276071-61276088 | 3.559 | 0.174 | 1.242 | 2.805 | 0.230 | 2.698 | 0.210 | 2.081 | 9.233 | 2.099 | 12.451 | 1.0916 | 0.2287 | 1.0000 |
| *hsa-miR-668-5p* | chr14:101521601-101521619 | 0.000 | 0.000 | 0.000 | 0.000 | 0.000 | 0.793 | 0.210 | 0.000 | 0.000 | 0.000 | 0.000 | 1.0912 | 0.3484 | 1.0000 |
| *hsa-miR-421* | chrX:73438227-73438249 | 10.845 | 22.059 | 10.761 | 17.097 | 35.630 | 17.931 | 31.849 | 52.447 | 44.420 | 16.530 | 26.977 | 1.0870 | 0.0209 | 0.8791 |
| *hsa-miR-6775-3p* | chr16:87868198-87868218 | 0.169 | 0.174 | 0.207 | 0.267 | 1.149 | 0.635 | 0.419 | 0.000 | 0.000 | 0.525 | 1.245 | 1.0837 | 0.3794 | 1.0000 |
| *hsa-miR-4517* | chr16:28969908-28969932 | 0.000 | 0.000 | 0.000 | 0.000 | 0.460 | 0.000 | 0.000 | 0.000 | 0.250 | 0.262 | 0.000 | 1.0790 | 0.3542 | 1.0000 |
| *hsa-miR-943* | chr4:1988130-1988150 | 0.169 | 0.695 | 0.414 | 0.401 | 0.000 | 1.428 | 0.838 | 1.665 | 0.749 | 1.050 | 1.453 | 1.0787 | 0.3030 | 1.0000 |
| *hsa-miR-663a* | chr20:26188879-26188900 | 0.678 | 0.521 | 0.207 | 4.675 | 6.206 | 0.952 | 1.886 | 1.249 | 3.743 | 3.673 | 5.603 | 1.0772 | 0.1838 | 1.0000 |
| *hsa-miR-3194-3p* | chr20:50069449-50069470 | 0.000 | 0.174 | 0.000 | 0.000 | 0.000 | 0.793 | 0.419 | 0.416 | 0.000 | 0.000 | 0.000 | 1.0749 | 0.4242 | 1.0000 |
| *hsa-miR-7702* | chr9:114033473-114033494 | 0.000 | 0.000 | 0.207 | 0.000 | 0.230 | 1.269 | 0.000 | 0.000 | 0.000 | 0.262 | 0.000 | 1.0727 | 0.4250 | 1.0000 |
| *hsa-miR-6716-5p* | chr11:118514733-118514752 | 0.000 | 0.000 | 0.000 | 0.000 | 0.230 | 0.317 | 0.419 | 0.000 | 0.000 | 0.000 | 0.000 | 1.0707 | 0.3561 | 1.0000 |
| *hsa-miR-5094* | chr15:90393922-90393943 | 0.169 | 0.000 | 0.000 | 0.000 | 0.230 | 0.000 | 0.000 | 0.000 | 0.499 | 0.000 | 0.830 | 1.0662 | 0.4315 | 1.0000 |
| *hsa-miR-4524a-5p* | chr17:67095747-67095768 | 0.169 | 0.174 | 1.242 | 0.134 | 1.609 | 4.602 | 0.210 | 1.249 | 0.000 | 0.000 | 0.000 | 1.0637 | 0.3184 | 1.0000 |
| *hsa-miR-7974* | chr19:11606359-11606382 | 3.050 | 4.342 | 0.414 | 0.000 | 5.287 | 0.793 | 2.724 | 2.081 | 6.987 | 1.837 | 7.471 | 1.0626 | 0.1713 | 1.0000 |
| *hsa-miR-183-3p* | chr7:129414768-129414789 | 4.745 | 6.774 | 7.036 | 6.946 | 9.425 | 9.203 | 10.686 | 12.487 | 14.474 | 18.892 | 16.809 | 1.0624 | 0.0414 | 1.0000 |
| *hsa-miR-4747-3p* | chr19:4932731-4932752 | 0.000 | 0.000 | 0.000 | 0.000 | 0.000 | 0.000 | 0.210 | 0.000 | 0.000 | 0.525 | 0.208 | 1.0621 | 0.3607 | 1.0000 |
| *hsa-miR-4657* | chr7:44921377-44921399 | 0.169 | 0.521 | 0.000 | 0.267 | 1.379 | 0.000 | 0.000 | 0.416 | 0.499 | 1.050 | 1.038 | 1.0618 | 0.3745 | 1.0000 |
| *hsa-miR-6818-3p* | chr22:30403081-30403102 | 0.169 | 0.000 | 0.000 | 0.000 | 0.230 | 0.000 | 0.000 | 0.832 | 0.000 | 0.525 | 0.000 | 1.0609 | 0.4299 | 1.0000 |
| *hsa-miR-5685* | chr6:53141800-53141821 | 0.000 | 0.000 | 0.000 | 0.000 | 0.230 | 0.317 | 0.000 | 0.416 | 0.000 | 0.000 | 0.000 | 1.0603 | 0.3579 | 1.0000 |
| *hsa-miR-4728-5p* | chr17:37882749-37882771 | 0.000 | 0.000 | 0.207 | 0.534 | 0.919 | 0.000 | 0.210 | 0.000 | 0.250 | 1.837 | 0.415 | 1.0591 | 0.3974 | 1.0000 |
| *hsa-miR-6799-3p* | chr19:50295169-50295191 | 0.000 | 0.174 | 0.207 | 0.134 | 0.690 | 0.635 | 0.629 | 0.416 | 0.000 | 0.262 | 0.208 | 1.0580 | 0.4167 | 1.0000 |
| *hsa-miR-6752-3p* | chr11:67257766-67257786 | 0.169 | 0.000 | 0.414 | 0.000 | 0.460 | 0.793 | 0.419 | 0.416 | 0.499 | 0.525 | 0.000 | 1.0565 | 0.4102 | 1.0000 |
| *hsa-miR-564* | chr3:44903395-44903413 | 0.000 | 0.174 | 0.000 | 0.000 | 0.000 | 0.000 | 0.000 | 0.416 | 0.250 | 0.262 | 0.623 | 1.0502 | 0.4381 | 1.0000 |
| *hsa-miR-6781-5p* | chr17:40975936-40975956 | 0.000 | 0.000 | 0.000 | 0.000 | 0.460 | 0.000 | 0.210 | 0.000 | 0.000 | 0.262 | 0.000 | 1.0484 | 0.3640 | 1.0000 |
| *hsa-miR-6513-3p* | chr2:219144850-219144870 | 0.508 | 0.174 | 0.414 | 0.668 | 0.690 | 0.793 | 0.629 | 2.914 | 0.998 | 1.050 | 0.415 | 1.0447 | 0.3120 | 1.0000 |
| *hsa-miR-518a-3p* | chr19:54234310-54234331; | 0.000 | 0.000 | 0.000 | 0.000 | 0.000 | 0.000 | 0.419 | 0.000 | 0.499 | 0.000 | 0.000 | 1.0375 | 0.3674 | 1.0000 |
|  | chr19:54242639-54242660 |  |  |  |  |  |  |  |  |  |  |  |  |  |  |
| *hsa-miR-1285-3p* | chr7:91833341-91833362; | 0.000 | 0.000 | 0.000 | 0.000 | 0.460 | 0.000 | 0.210 | 0.000 | 0.250 | 0.000 | 0.000 | 1.0364 | 0.3675 | 1.0000 |
|  | chr2:70480065-70480086 |  |  |  |  |  |  |  |  |  |  |  |  |  |  |
| *hsa-miR-6132* | chr7:116660285-116660303 | 0.000 | 0.000 | 0.000 | 0.000 | 0.460 | 0.000 | 0.210 | 0.000 | 0.250 | 0.000 | 0.000 | 1.0364 | 0.3675 | 1.0000 |
| *hsa-miR-6740-3p* | chr1:201972343-201972364 | 0.000 | 0.000 | 0.000 | 0.134 | 0.460 | 0.317 | 0.629 | 0.000 | 0.000 | 0.000 | 0.000 | 1.0358 | 0.4378 | 1.0000 |
| *hsa-miR-3161* | chr11:48118343-48118365 | 0.000 | 0.000 | 0.000 | 0.000 | 0.919 | 0.000 | 0.000 | 0.000 | 0.000 | 0.000 | 0.000 | 1.0354 | 0.3675 | 1.0000 |
| *hsa-miR-4649-3p* | chr7:44150490-44150510 | 0.508 | 1.563 | 1.655 | 0.801 | 4.368 | 2.698 | 2.724 | 5.827 | 0.499 | 1.312 | 0.208 | 1.0337 | 0.2055 | 1.0000 |
| *hsa-miR-6800-3p* | chr19:50335333-50335353 | 0.000 | 0.174 | 0.000 | 0.801 | 0.690 | 0.952 | 0.419 | 1.249 | 0.998 | 0.000 | 0.208 | 1.0335 | 0.3806 | 1.0000 |
| *hsa-miR-2467-3p* | chr2:240273427-240273448 | 0.000 | 0.000 | 0.000 | 0.000 | 0.230 | 0.476 | 0.000 | 0.000 | 0.000 | 0.000 | 0.208 | 1.0275 | 0.3695 | 1.0000 |
| *hsa-miR-6506-5p* | chr16:15704929-15704951 | 0.000 | 0.000 | 0.000 | 0.134 | 0.000 | 0.000 | 0.419 | 0.000 | 0.749 | 0.000 | 0.208 | 1.0238 | 0.4441 | 1.0000 |
| *hsa-miR-1304-5p* | chr11:93466890-93466911 | 0.339 | 1.042 | 0.207 | 1.202 | 1.609 | 1.745 | 1.257 | 0.832 | 1.996 | 0.787 | 2.490 | 1.0216 | 0.2672 | 1.0000 |
| *hsa-miR-4785* | chr2:161264330-161264350 | 0.508 | 0.347 | 0.000 | 0.401 | 0.919 | 1.904 | 0.838 | 0.832 | 0.499 | 0.262 | 0.208 | 1.0199 | 0.3631 | 1.0000 |
| *hsa-miR-320b* | chr1:117214409-117214430; | 248.087 | 373.955 | 356.760 | 573.564 | 901.545 | 536.335 | 421.574 | 391.272 | 321.922 | 893.944 | 1693.126 | 1.0157 | 0.0827 | 1.0000 |
|  | chr1:224444751-224444772 |  |  |  |  |  |  |  |  |  |  |  |  |  |  |
| *hsa-miR-33a-5p* | chr22:42296953-42296973 | 0.000 | 0.174 | 1.242 | 0.267 | 0.000 | 1.269 | 0.000 | 3.330 | 0.998 | 1.050 | 0.415 | 1.0109 | 0.3398 | 1.0000 |
| *hsa-miR-7111-5p* | chr6:35438286-35438307 | 0.000 | 0.000 | 0.000 | 0.000 | 0.230 | 0.000 | 0.000 | 0.416 | 0.250 | 0.000 | 0.000 | 1.0107 | 0.3744 | 1.0000 |
| *hsa-miR-1825* | chr20:30825633-30825650 | 0.000 | 0.000 | 0.000 | 0.000 | 0.000 | 0.000 | 0.210 | 0.416 | 0.000 | 0.262 | 0.000 | 1.0076 | 0.3760 | 1.0000 |
| *hsa-miR-516b-5p* | chr19:54228711-54228732; | 0.000 | 0.000 | 0.000 | 0.000 | 0.000 | 0.000 | 0.210 | 0.416 | 0.000 | 0.262 | 0.000 | 1.0076 | 0.3760 | 1.0000 |
|  | chr19:54240114-54240135 |  |  |  |  |  |  |  |  |  |  |  |  |  |  |
| *hsa-miR-659-3p* | chr22:38243700-38243721 | 0.000 | 0.000 | 0.000 | 0.000 | 0.000 | 0.000 | 0.210 | 0.416 | 0.000 | 0.262 | 0.000 | 1.0076 | 0.3760 | 1.0000 |
| *hsa-miR-497-3p* | chr17:6921257-6921278 | 0.169 | 0.000 | 0.000 | 0.000 | 0.230 | 0.000 | 0.629 | 0.416 | 0.000 | 0.000 | 0.208 | 1.0070 | 0.4539 | 1.0000 |
| *hsa-miR-6885-3p* | chr19:6389649-6389669 | 0.339 | 0.174 | 0.207 | 0.267 | 0.919 | 1.111 | 0.210 | 0.416 | 0.749 | 0.787 | 0.208 | 1.0052 | 0.3944 | 1.0000 |
| *hsa-miR-3193* | chr20:30194989-30195010 | 0.000 | 0.000 | 0.000 | 0.000 | 0.000 | 0.476 | 0.000 | 0.416 | 0.000 | 0.000 | 0.000 | 1.0011 | 0.3763 | 1.0000 |
