# Supplementary material for: The microRNA expression signature of pancreatic ductal adenocarcinoma by RNA sequencing: anti-tumour functions of the microRNA-216 cluster
Source: Oncotarget. 2017 Jul 26;8(41):70097–115. doi: 10.18632/oncotarget.19591 (PMC5642539; doi:10.18632/oncotarget.19591)
Supplement: Supplementary file 3 [file oncotarget-08-70097-s003.docx]

**Supplementary Table 1**: Downregulated miRNAs in PDAC

| **miRNA** | **locus** | **N1** | **N2** | **N3** | **N4** | **T1** | **T2** | **T3** | **T4** | **T5** | **T6** | **T7** | **logFC** | **PValue** | **FDR** |
| --- | --- | --- | --- | --- | --- | --- | --- | --- | --- | --- | --- | --- | --- | --- | --- |
| *hsa-miR-216a-5p* | chr2:56216155-56216176 | 1707.630 | 822.423 | 674.409 | 212.783 | 3.678 | 358.456 | 299.837 | 7.076 | 9.483 | 3.149 | 1.245 | -3.2184 | 0.0033 | 0.4602 |
| *hsa-miR-802* | chr21:37093030-37093052 | 13.048 | 5.037 | 10.347 | 0.801 | 0.000 | 3.808 | 0.629 | 0.000 | 0.749 | 0.000 | 0.000 | -3.1703 | 0.0001 | 0.0901 |
| *hsa-miR-3186-3p* | chr17:79418142-79418162 | 3.728 | 0.174 | 0.000 | 0.000 | 0.000 | 0.000 | 0.000 | 0.000 | 0.000 | 0.000 | 0.000 | -3.1608 | 0.0072 | 0.5030 |
| *hsa-miR-217* | chr2:56210155-56210177 | 220.126 | 154.932 | 153.754 | 34.596 | 0.919 | 57.759 | 55.106 | 1.249 | 0.499 | 1.050 | 0.000 | -3.1333 | 0.0034 | 0.4602 |
| *hsa-miR-2114-5p* | chrX:149396251-149396272 | 11.693 | 0.347 | 19.866 | 0.000 | 0.000 | 5.712 | 0.000 | 0.832 | 0.000 | 0.000 | 0.208 | -3.0839 | 0.0033 | 0.4602 |
| *hsa-miR-216b-5p* | chr2:56227899-56227920 | 825.939 | 916.737 | 881.760 | 186.068 | 0.230 | 525.228 | 249.550 | 0.416 | 5.740 | 1.312 | 1.038 | -2.7668 | 0.0286 | 0.9691 |
| *hsa-miR-3186-5p* | chr17:79418178-79418199 | 2.711 | 0.000 | 0.000 | 0.000 | 0.000 | 0.000 | 0.000 | 0.000 | 0.000 | 0.000 | 0.000 | -2.7046 | 0.0230 | 0.9200 |
| *hsa-miR-216b-3p* | chr2:56227859-56227882 | 11.693 | 8.685 | 7.864 | 2.004 | 0.000 | 4.284 | 3.981 | 0.000 | 0.000 | 0.000 | 0.000 | -2.5792 | 0.0008 | 0.1722 |
| *hsa-miR-6510-3p* | chr17:39673418-39673438 | 0.339 | 0.000 | 39.732 | 0.534 | 2.069 | 2.539 | 1.467 | 0.832 | 3.494 | 0.262 | 1.245 | -2.5658 | 0.0098 | 0.6010 |
| *hsa-miR-122-5p* | chr18:56118320-56118341 | 0.000 | 0.000 | 0.828 | 25.379 | 5.517 | 0.476 | 0.419 | 0.832 | 0.749 | 0.000 | 0.415 | -2.5149 | 0.0207 | 0.8791 |
| *hsa-miR-412-3p* | chr14:101531837-101531859 | 0.508 | 1.216 | 1.449 | 0.401 | 0.000 | 0.000 | 0.210 | 0.000 | 0.250 | 0.000 | 0.000 | -2.4160 | 0.0393 | 1.0000 |
| *hsa-miR-129-1-3p* | chr7:127847973-127847994 | 35.247 | 55.233 | 70.152 | 18.300 | 0.230 | 34.116 | 12.153 | 4.162 | 0.749 | 8.659 | 1.660 | -2.4045 | 0.0011 | 0.2082 |
| *hsa-miR-216a-3p* | chr2:56216116-56216137 | 235.208 | 121.062 | 206.523 | 57.704 | 4.597 | 132.814 | 84.860 | 2.914 | 1.248 | 0.787 | 0.415 | -2.3827 | 0.0214 | 0.8791 |
| *hsa-miR-135a-3p* | chr3:52328248-52328269 | 3.220 | 27.269 | 6.001 | 8.148 | 2.988 | 7.617 | 1.467 | 0.416 | 0.000 | 0.525 | 1.038 | -2.3680 | 0.0008 | 0.1722 |
| *hsa-miR-148a-5p* | chr7:25989580-25989601 | 1110.629 | 625.111 | 1198.374 | 299.872 | 44.365 | 749.124 | 211.625 | 114.884 | 91.585 | 45.130 | 30.712 | -2.3043 | 0.0009 | 0.1722 |
| *hsa-miR-891a-5p* | chrX:145109359-145109380 | 27.452 | 23.274 | 19.038 | 72.530 | 0.460 | 44.906 | 6.286 | 1.665 | 2.246 | 3.936 | 1.660 | -2.2454 | 0.0053 | 0.5030 |
| *hsa-miR-190b* | chr1:154166189-154166209 | 26.097 | 20.148 | 36.007 | 19.635 | 2.758 | 22.215 | 7.543 | 3.330 | 1.996 | 1.312 | 3.943 | -2.1240 | 0.0003 | 0.1429 |
| *hsa-miR-148a-3p* | chr7:25989542-25989563 | 14068.251 | 12716.904 | 9114.348 | 3617.303 | 664.550 | 5714.988 | 3843.199 | 2413.401 | 2055.308 | 955.866 | 807.031 | -2.1217 | 0.0001 | 0.0901 |
| *hsa-miR-211-5p* | chr15:31357298-31357319 | 2.542 | 2.953 | 10.761 | 0.935 | 0.460 | 2.539 | 0.210 | 0.416 | 2.745 | 0.000 | 0.208 | -2.1027 | 0.0084 | 0.5545 |
| *hsa-miR-1224-5p* | chr3:183959193-183959211 | 64.394 | 32.133 | 330.893 | 103.920 | 0.460 | 218.819 | 5.867 | 11.239 | 2.745 | 33.323 | 1.245 | -2.0477 | 0.0450 | 1.0000 |
| *hsa-miR-338-5p* | chr17:79099723-79099744 | 97.100 | 122.452 | 38.904 | 50.224 | 9.884 | 32.529 | 10.686 | 17.482 | 12.727 | 43.556 | 4.150 | -2.0236 | 0.0002 | 0.0956 |
| *hsa-miR-129-2-3p* | chr11:43603000-43603021 | 178.778 | 91.882 | 149.823 | 27.917 | 0.690 | 85.845 | 46.306 | 17.482 | 2.246 | 36.471 | 13.489 | -2.0092 | 0.0090 | 0.5650 |
| *hsa-miR-137* | chr1:98511647-98511669 | 1.186 | 0.174 | 0.000 | 0.134 | 0.000 | 0.000 | 0.000 | 0.000 | 0.000 | 0.000 | 0.000 | -2.0061 | 0.0874 | 1.0000 |
| *hsa-miR-885-5p* | chr3:10436215-10436236 | 3.559 | 5.732 | 6.001 | 1.469 | 0.230 | 3.491 | 0.419 | 0.000 | 1.248 | 0.525 | 0.830 | -1.9933 | 0.0072 | 0.5030 |
| *hsa-miR-1251-5p* | chr12:97885691-97885711 | 4.745 | 0.000 | 0.828 | 0.267 | 0.230 | 0.476 | 0.210 | 0.416 | 0.250 | 0.262 | 0.208 | -1.9657 | 0.0537 | 1.0000 |
| *hsa-miR-2114-3p* | chrX:149396287-149396307 | 6.100 | 0.174 | 5.380 | 0.534 | 0.000 | 2.698 | 1.676 | 0.000 | 0.000 | 0.525 | 0.208 | -1.9620 | 0.0257 | 0.9200 |
| *hsa-miR-154-5p* | chr14:101526106-101526127 | 32.536 | 81.808 | 18.210 | 18.433 | 5.517 | 30.625 | 5.657 | 7.492 | 8.235 | 6.822 | 3.113 | -1.9529 | 0.0004 | 0.1429 |
| *hsa-miR-7-2-3p* | chr15:89155127-89155148 | 0.169 | 0.174 | 1.449 | 0.267 | 0.000 | 0.317 | 0.000 | 0.000 | 0.000 | 0.000 | 0.000 | -1.9294 | 0.1184 | 1.0000 |
| *hsa-miR-3144-3p* | chr6:120336372-120336393 | 2.711 | 0.521 | 0.621 | 0.267 | 0.000 | 0.793 | 0.419 | 0.000 | 0.000 | 0.262 | 0.000 | -1.8068 | 0.0947 | 1.0000 |
| *hsa-miR-551b-3p* | chr3:168269702-168269722 | 1.356 | 5.037 | 10.761 | 2.671 | 0.230 | 4.284 | 1.467 | 0.832 | 1.747 | 1.050 | 0.000 | -1.8055 | 0.0130 | 0.6906 |
| *hsa-miR-3529-5p* | chr15:89155125-89155146 | 0.678 | 0.174 | 1.035 | 0.668 | 0.000 | 0.476 | 0.000 | 0.000 | 0.000 | 0.000 | 0.208 | -1.8032 | 0.1328 | 1.0000 |
| *hsa-miR-4780* | chr2:88382047-88382068 | 0.508 | 1.911 | 3.104 | 0.801 | 0.000 | 2.222 | 0.210 | 0.000 | 0.000 | 0.000 | 0.208 | -1.7904 | 0.0638 | 1.0000 |
| *hsa-miR-1250-5p* | chr17:79107065-79107085 | 3.728 | 1.390 | 2.276 | 1.069 | 0.460 | 2.222 | 0.210 | 0.416 | 0.749 | 0.000 | 0.000 | -1.7163 | 0.0487 | 1.0000 |
| *hsa-miR-375* | chr2:219866370-219866391 | 115840.614 | 45896.429 | 92195.959 | 42261.575 | 578.119 | 90230.189 | 16704.986 | 13262.466 | 4685.084 | 29917.071 | 18010.138 | -1.6934 | 0.0253 | 0.9200 |
| *hsa-miR-129-5p* | chr7:127847929-127847949; | 775.102 | 474.869 | 787.603 | 570.092 | 2.299 | 1169.783 | 161.338 | 121.544 | 7.986 | 148.510 | 56.444 | -1.6670 | 0.0632 | 1.0000 |
|  | chr11:43602958-43602978 |  |  |  |  |  |  |  |  |  |  |  |  |  |  |
| *hsa-miR-1179* | chr15:89151352-89151372 | 1.017 | 0.868 | 0.828 | 0.534 | 0.000 | 0.793 | 0.210 | 0.000 | 0.000 | 0.000 | 0.208 | -1.6664 | 0.1415 | 1.0000 |
| *hsa-miR-451a* | chr17:27188421-27188442 | 370.266 | 293.884 | 1624.458 | 2579.437 | 1420.588 | 619.007 | 58.040 | 391.689 | 223.349 | 355.793 | 28.637 | -1.6505 | 0.0341 | 1.0000 |
| *hsa-miR-204-5p* | chr9:73424947-73424968 | 580.564 | 840.313 | 1284.460 | 632.737 | 15.401 | 1378.763 | 107.908 | 205.210 | 105.311 | 269.731 | 56.029 | -1.6320 | 0.0290 | 0.9691 |
| *hsa-miR-655-3p* | chr14:101515947-101515968 | 1.525 | 3.647 | 1.035 | 0.401 | 0.230 | 0.635 | 0.629 | 0.832 | 0.250 | 0.525 | 0.000 | -1.6264 | 0.0791 | 1.0000 |
| *hsa-miR-130b-5p* | chr22:22007605-22007625 | 180.812 | 132.352 | 162.239 | 42.209 | 13.332 | 147.730 | 55.525 | 20.396 | 36.185 | 16.005 | 25.940 | -1.5926 | 0.0069 | 0.5030 |
| *hsa-miR-6855-3p* | chr9:132631929-132631950 | 0.000 | 0.521 | 0.621 | 0.267 | 0.000 | 0.000 | 0.000 | 0.000 | 0.250 | 0.000 | 0.000 | -1.5724 | 0.2072 | 1.0000 |
| *hsa-miR-6514-3p* | chr11:62560174-62560194 | 0.339 | 0.868 | 0.414 | 0.801 | 0.000 | 0.476 | 0.419 | 0.000 | 0.000 | 0.000 | 0.000 | -1.5395 | 0.1958 | 1.0000 |
| *hsa-miR-548v* | chr8:17539097-17539118 | 0.169 | 0.347 | 0.414 | 0.000 | 0.000 | 0.000 | 0.000 | 0.000 | 0.000 | 0.000 | 0.000 | -1.5169 | 0.1750 | 1.0000 |
| *hsa-miR-4423-5p* | chr1:85599489-85599510 | 0.169 | 1.911 | 0.621 | 0.000 | 0.000 | 0.317 | 0.000 | 0.416 | 0.250 | 0.000 | 0.208 | -1.4188 | 0.2186 | 1.0000 |
| *hsa-miR-138-2-3p* | chr16:56892486-56892507 | 0.508 | 0.000 | 0.414 | 0.134 | 0.000 | 0.159 | 0.000 | 0.000 | 0.000 | 0.000 | 0.000 | -1.4125 | 0.2535 | 1.0000 |
| *hsa-miR-4636* | chr5:9053977-9053998 | 1.356 | 0.521 | 0.414 | 0.000 | 0.000 | 0.000 | 0.000 | 0.000 | 0.499 | 0.262 | 0.208 | -1.4052 | 0.2379 | 1.0000 |
| *hsa-miR-130a-5p* | chr11:57408691-57408712 | 0.847 | 3.300 | 0.621 | 0.668 | 0.230 | 0.476 | 0.210 | 0.416 | 0.998 | 0.262 | 0.415 | -1.3921 | 0.1460 | 1.0000 |
| *hsa-miR-30c-5p* | chr6:72086706-72086728; | 6728.338 | 13617.836 | 5058.997 | 3096.366 | 1337.835 | 4135.178 | 2118.558 | 2795.516 | 3502.459 | 2350.177 | 2135.966 | -1.3805 | 0.0007 | 0.1722 |
|  | chr1:41222972-41222994 |  |  |  |  |  |  |  |  |  |  |  |  |  |  |
| *hsa-miR-3612* | chr12:128778650-128778671 | 0.169 | 0.000 | 0.621 | 0.000 | 0.000 | 0.000 | 0.000 | 0.000 | 0.000 | 0.000 | 0.000 | -1.3769 | 0.2095 | 1.0000 |
| *hsa-miR-381-5p* | chr14:101512264-101512285 | 1.864 | 3.647 | 2.483 | 2.538 | 0.230 | 1.904 | 1.676 | 1.665 | 0.499 | 0.525 | 0.208 | -1.3671 | 0.0773 | 1.0000 |
| *hsa-miR-548ag* | chr4:61788341-61788361; | 0.000 | 0.695 | 0.414 | 0.000 | 0.000 | 0.000 | 0.210 | 0.000 | 0.000 | 0.000 | 0.000 | -1.3668 | 0.2693 | 1.0000 |
|  | chr20:59139623-59139643 |  |  |  |  |  |  |  |  |  |  |  |  |  |  |
| *hsa-miR-6843-3p* | chr8:27468118-27468138 | 0.339 | 0.174 | 0.207 | 0.668 | 0.000 | 0.000 | 0.000 | 0.000 | 0.000 | 0.000 | 0.415 | -1.3643 | 0.2778 | 1.0000 |
| *hsa-miR-19a-3p* | chr13:92003193-92003215 | 1.186 | 10.421 | 3.311 | 0.401 | 1.839 | 0.317 | 2.095 | 2.914 | 1.248 | 0.787 | 0.208 | -1.3565 | 0.0722 | 1.0000 |
| *hsa-miR-496* | chr14:101526965-101526986 | 0.508 | 6.079 | 2.069 | 1.870 | 0.460 | 2.380 | 1.676 | 0.832 | 1.248 | 0.000 | 0.000 | -1.3514 | 0.0906 | 1.0000 |
| *hsa-miR-7855-5p* | chr14:65252381-65252402 | 0.000 | 0.000 | 0.621 | 0.134 | 0.000 | 0.000 | 0.000 | 0.000 | 0.000 | 0.000 | 0.000 | -1.3371 | 0.2199 | 1.0000 |
| *hsa-miR-548j-5p* | chr22:26951240-26951261 | 0.339 | 1.563 | 0.207 | 1.069 | 0.000 | 0.159 | 0.419 | 0.416 | 0.250 | 0.000 | 0.415 | -1.3369 | 0.2276 | 1.0000 |
| *hsa-miR-3914* | chr7:70772673-70772694; | 0.000 | 0.347 | 0.414 | 0.000 | 0.000 | 0.000 | 0.000 | 0.000 | 0.000 | 0.000 | 0.000 | -1.3334 | 0.2190 | 1.0000 |
|  | chr7:70772720-70772741 |  |  |  |  |  |  |  |  |  |  |  |  |  |  |
| *hsa-miR-379-3p* | chr14:101488446-101488467 | 3.389 | 5.732 | 1.862 | 1.603 | 1.149 | 1.269 | 1.676 | 2.914 | 0.749 | 0.525 | 0.000 | -1.3229 | 0.0731 | 1.0000 |
| *hsa-miR-135a-5p* | chr3:52328286-52328308; | 17.285 | 30.917 | 2.897 | 6.946 | 1.839 | 3.174 | 15.505 | 3.330 | 4.492 | 7.872 | 0.830 | -1.3206 | 0.0497 | 1.0000 |
|  | chr12:97957612-97957634 |  |  |  |  |  |  |  |  |  |  |  |  |  |  |
| *hsa-miR-4507* | chr14:106324294-106324313 | 0.169 | 0.174 | 0.621 | 0.000 | 0.000 | 0.159 | 0.000 | 0.000 | 0.000 | 0.000 | 0.000 | -1.3176 | 0.2842 | 1.0000 |
| *hsa-miR-539-5p* | chr14:101513666-101513687 | 4.406 | 15.285 | 2.483 | 6.011 | 1.379 | 5.712 | 1.886 | 4.995 | 1.248 | 3.149 | 0.830 | -1.3132 | 0.0336 | 1.0000 |
| *hsa-miR-16-1-3p* | chr13:50623121-50623142 | 0.339 | 0.695 | 0.000 | 0.401 | 0.230 | 0.000 | 0.000 | 0.000 | 0.000 | 0.262 | 0.000 | -1.3047 | 0.2973 | 1.0000 |
| *hsa-miR-488-3p* | chr1:176998510-176998530 | 0.508 | 0.174 | 0.207 | 0.134 | 0.000 | 0.000 | 0.210 | 0.000 | 0.000 | 0.000 | 0.000 | -1.3031 | 0.2946 | 1.0000 |
| *hsa-miR-4732-5p* | chr17:27188718-27188740 | 0.000 | 0.000 | 2.897 | 3.206 | 2.758 | 0.476 | 0.000 | 0.000 | 0.000 | 0.787 | 0.000 | -1.3026 | 0.1787 | 1.0000 |
| *hsa-miR-30c-2-3p* | chr6:72086667-72086688 | 135.228 | 265.051 | 136.372 | 99.112 | 30.573 | 148.048 | 66.421 | 27.472 | 47.664 | 40.145 | 76.159 | -1.2993 | 0.0065 | 0.5030 |
| *hsa-miR-6833-3p* | chr6:32147633-32147653 | 1.186 | 1.563 | 2.897 | 1.870 | 0.460 | 1.745 | 0.419 | 0.416 | 0.749 | 0.525 | 0.623 | -1.2972 | 0.1285 | 1.0000 |
| *hsa-miR-576-5p* | chr4:110409869-110409890 | 8.473 | 7.990 | 3.725 | 2.671 | 3.908 | 2.380 | 2.724 | 1.249 | 2.745 | 1.574 | 1.038 | -1.2962 | 0.0364 | 1.0000 |
| *hsa-miR-3938* | chr3:55886543-55886564 | 1.017 | 0.521 | 0.207 | 0.000 | 0.000 | 0.317 | 0.419 | 0.000 | 0.000 | 0.000 | 0.000 | -1.2899 | 0.2955 | 1.0000 |
| *hsa-miR-30a-3p* | chr6:72113257-72113278 | 584.461 | 742.178 | 287.436 | 269.151 | 59.536 | 298.793 | 206.597 | 131.950 | 205.631 | 118.335 | 259.603 | -1.2872 | 0.0064 | 0.5030 |
| *hsa-miR-4641* | chr6:41566502-41566525 | 1.864 | 0.521 | 0.207 | 0.267 | 0.230 | 0.635 | 0.210 | 0.000 | 0.499 | 0.000 | 0.000 | -1.2823 | 0.2572 | 1.0000 |
| *hsa-miR-5589-3p* | chr19:10149067-10149089 | 0.508 | 0.521 | 0.000 | 0.000 | 0.230 | 0.000 | 0.000 | 0.000 | 0.000 | 0.000 | 0.000 | -1.2730 | 0.3055 | 1.0000 |
| *hsa-miR-30a-5p* | chr6:72113298-72113319 | 16352.209 | 20217.717 | 10320.792 | 7382.472 | 3208.506 | 7758.299 | 10132.031 | 3555.999 | 5510.352 | 3219.457 | 4547.306 | -1.2695 | 0.0036 | 0.4661 |
| *hsa-miR-503-5p* | chrX:133680401-133680423 | 14.912 | 57.491 | 5.587 | 22.707 | 5.517 | 33.164 | 20.743 | 7.076 | 3.743 | 2.361 | 0.830 | -1.2602 | 0.0745 | 1.0000 |
| *hsa-miR-323a-3p* | chr14:101492119-101492139 | 24.741 | 60.965 | 52.355 | 61.310 | 11.034 | 107.743 | 14.039 | 14.152 | 7.487 | 8.921 | 2.283 | -1.2519 | 0.0673 | 1.0000 |
| *hsa-miR-2682-3p* | chr1:98510827-98510848 | 0.339 | 0.347 | 0.000 | 0.000 | 0.000 | 0.000 | 0.000 | 0.000 | 0.000 | 0.000 | 0.000 | -1.2444 | 0.2428 | 1.0000 |
| *hsa-miR-1269a* | chr4:67142608-67142629 | 1.017 | 0.000 | 0.000 | 0.134 | 0.000 | 0.159 | 0.210 | 0.000 | 0.000 | 0.000 | 0.000 | -1.2309 | 0.3308 | 1.0000 |
| *hsa-miR-30b-5p* | chr8:135812813-135812834 | 2510.184 | 2842.788 | 621.433 | 631.135 | 313.541 | 810.692 | 479.195 | 922.820 | 933.324 | 644.941 | 635.000 | -1.2298 | 0.0087 | 0.5588 |
| *hsa-miR-193a-3p* | chr17:29887069-29887090 | 34.061 | 56.797 | 42.836 | 14.025 | 13.103 | 36.179 | 25.772 | 24.559 | 6.488 | 6.297 | 2.490 | -1.2081 | 0.0281 | 0.9691 |
| *hsa-miR-491-5p* | chr9:20716119-20716140 | 12.709 | 32.306 | 29.385 | 8.816 | 6.896 | 20.787 | 6.495 | 8.325 | 6.239 | 11.807 | 2.905 | -1.1966 | 0.0183 | 0.8589 |
| *hsa-miR-5189-5p* | chr16:88535351-88535374 | 0.508 | 0.000 | 0.828 | 1.202 | 0.000 | 0.159 | 0.210 | 0.000 | 0.499 | 0.000 | 0.623 | -1.1895 | 0.3025 | 1.0000 |
| *hsa-miR-3133* | chr2:242417329-242417350 | 0.339 | 0.347 | 0.414 | 0.134 | 0.000 | 0.000 | 0.210 | 0.000 | 0.250 | 0.000 | 0.000 | -1.1883 | 0.3463 | 1.0000 |
| *hsa-miR-3178* | chr16:2581980-2581996 | 0.000 | 0.000 | 0.621 | 0.000 | 0.000 | 0.000 | 0.000 | 0.000 | 0.000 | 0.000 | 0.000 | -1.1722 | 0.2651 | 1.0000 |
| *hsa-miR-9-3p* | chr1:156390146-156390167; | 0.000 | 0.000 | 0.621 | 0.000 | 0.000 | 0.000 | 0.000 | 0.000 | 0.000 | 0.000 | 0.000 | -1.1722 | 0.2651 | 1.0000 |
|  | chr5:87962684-87962705; |  |  |  |  |  |  |  |  |  |  |  |  |  |  |
|  | chr15:89911302-89911323 |  |  |  |  |  |  |  |  |  |  |  |  |  |  |
| *hsa-miR-4802-5p* | chr4:40504103-40504124 | 0.000 | 0.347 | 0.414 | 0.134 | 0.000 | 0.000 | 0.210 | 0.000 | 0.000 | 0.000 | 0.000 | -1.1705 | 0.3436 | 1.0000 |
| *hsa-miR-378b* | chr3:10371946-10371964 | 0.169 | 0.868 | 0.207 | 0.134 | 0.000 | 0.317 | 0.000 | 0.000 | 0.000 | 0.262 | 0.000 | -1.1697 | 0.3503 | 1.0000 |
| *hsa-miR-2681-5p* | chr13:102620054-102620075 | 0.339 | 0.000 | 1.655 | 0.134 | 0.230 | 0.159 | 0.419 | 0.000 | 0.000 | 0.000 | 0.415 | -1.1449 | 0.3358 | 1.0000 |
| *hsa-miR-494-3p* | chr14:101496018-101496039 | 3.728 | 15.632 | 3.518 | 5.610 | 2.299 | 5.078 | 6.286 | 1.665 | 1.497 | 4.198 | 0.415 | -1.1329 | 0.0692 | 1.0000 |
| *hsa-miR-4670-3p* | chr9:95290273-95290294 | 0.000 | 0.347 | 0.414 | 0.534 | 0.000 | 0.159 | 0.000 | 0.416 | 0.000 | 0.000 | 0.000 | -1.1324 | 0.3712 | 1.0000 |
| *hsa-miR-130b-3p* | chr22:22007643-22007664 | 454.826 | 271.478 | 130.991 | 98.711 | 77.466 | 159.790 | 170.976 | 98.651 | 151.228 | 41.194 | 71.178 | -1.1215 | 0.0215 | 0.8791 |
| *hsa-miR-382-3p* | chr14:101520689-101520709 | 2.372 | 1.911 | 2.276 | 0.801 | 0.460 | 1.428 | 0.838 | 1.665 | 0.749 | 0.262 | 0.208 | -1.1112 | 0.1869 | 1.0000 |
| *hsa-miR-218-2-3p* | chr5:168195173-168195194 | 0.169 | 0.174 | 1.862 | 0.668 | 0.000 | 1.745 | 0.000 | 0.000 | 0.000 | 0.000 | 0.208 | -1.1048 | 0.3228 | 1.0000 |
| *hsa-miR-19b-3p* | chr13:92003499-92003521; | 29.486 | 262.272 | 59.391 | 22.173 | 51.491 | 19.041 | 50.916 | 62.853 | 36.185 | 50.115 | 6.848 | -1.0940 | 0.0731 | 1.0000 |
|  | chrX:133303713-133303735 |  |  |  |  |  |  |  |  |  |  |  |  |  |  |
| *hsa-miR-541-5p* | chr14:101530841-101530865 | 1.356 | 0.868 | 0.207 | 2.805 | 0.690 | 2.063 | 0.629 | 0.416 | 0.000 | 0.000 | 0.208 | -1.0932 | 0.2484 | 1.0000 |
| *hsa-miR-6884-5p* | chr17:38182636-38182657 | 0.000 | 0.174 | 1.035 | 0.134 | 0.230 | 0.159 | 0.000 | 0.000 | 0.000 | 0.262 | 0.000 | -1.0894 | 0.3875 | 1.0000 |
| *hsa-miR-377-5p* | chr14:101528393-101528414 | 80.493 | 180.290 | 115.885 | 133.573 | 47.813 | 213.582 | 56.154 | 58.275 | 31.194 | 36.209 | 10.791 | -1.0878 | 0.0447 | 1.0000 |
| *hsa-miR-670-3p* | chr11:43581264-43581284 | 0.169 | 0.000 | 1.035 | 0.267 | 0.000 | 0.159 | 0.210 | 0.416 | 0.000 | 0.000 | 0.000 | -1.0797 | 0.3898 | 1.0000 |
| *hsa-miR-29c-5p* | chr1:207975248-207975269 | 93.880 | 158.058 | 116.299 | 65.184 | 38.158 | 62.520 | 42.744 | 36.213 | 61.390 | 78.715 | 29.882 | -1.0765 | 0.0065 | 0.5030 |
| *hsa-miR-4659a-3p* | chr8:6602733-6602754 | 0.000 | 0.000 | 0.414 | 0.134 | 0.000 | 0.000 | 0.000 | 0.000 | 0.000 | 0.000 | 0.000 | -1.0752 | 0.2947 | 1.0000 |
| *hsa-miR-4294* | chr10:50193606-50193622 | 0.000 | 0.347 | 0.207 | 0.000 | 0.000 | 0.000 | 0.000 | 0.000 | 0.000 | 0.000 | 0.000 | -1.0729 | 0.2931 | 1.0000 |
| *hsa-miR-4772-3p* | chr2:103048796-103048817 | 0.339 | 1.042 | 0.621 | 0.801 | 0.230 | 0.159 | 0.210 | 0.832 | 0.499 | 0.000 | 0.000 | -1.0569 | 0.3439 | 1.0000 |
| *hsa-miR-5193* | chr3:49843598-49843619 | 0.678 | 1.737 | 0.828 | 0.935 | 0.000 | 0.159 | 0.419 | 0.000 | 1.248 | 0.787 | 0.415 | -1.0533 | 0.2896 | 1.0000 |
| *hsa-miR-6868-3p* | chr17:74094100-74094120 | 0.339 | 0.174 | 0.207 | 0.000 | 0.000 | 0.159 | 0.000 | 0.000 | 0.000 | 0.000 | 0.000 | -1.0520 | 0.3853 | 1.0000 |
| *hsa-miR-487a-5p* | chr14:101518795-101518816 | 6.609 | 16.501 | 19.452 | 17.365 | 5.057 | 27.293 | 6.914 | 7.076 | 2.745 | 4.723 | 0.830 | -1.0495 | 0.0818 | 1.0000 |
| *hsa-miR-29c-3p* | chr1:207975210-207975231 | 247.578 | 198.528 | 135.544 | 49.289 | 41.836 | 105.839 | 36.668 | 144.854 | 86.345 | 116.761 | 24.072 | -1.0248 | 0.0414 | 1.0000 |
| *hsa-miR-4695-5p* | chr1:19209744-19209765 | 0.000 | 0.174 | 0.207 | 0.134 | 0.000 | 0.000 | 0.000 | 0.000 | 0.000 | 0.000 | 0.000 | -1.0230 | 0.3103 | 1.0000 |
| *hsa-miR-495-5p* | chr14:101500105-101500126 | 0.000 | 0.695 | 0.828 | 0.267 | 0.000 | 0.635 | 0.210 | 0.000 | 0.000 | 0.262 | 0.000 | -1.0216 | 0.4003 | 1.0000 |
| *hsa-miR-7973* | chr15:51606231-51606250; | 0.000 | 0.521 | 0.000 | 0.000 | 0.000 | 0.000 | 0.000 | 0.000 | 0.000 | 0.000 | 0.000 | -1.0211 | 0.3084 | 1.0000 |
|  | chr15:51606285-51606304 |  |  |  |  |  |  |  |  |  |  |  |  |  |  |
| *hsa-miR-1911-5p* | chrX:113997755-113997777 | 0.169 | 0.347 | 0.000 | 0.000 | 0.000 | 0.000 | 0.000 | 0.000 | 0.000 | 0.000 | 0.000 | -1.0203 | 0.3098 | 1.0000 |
| *hsa-miR-7152-5p* | chr10:73550504-73550525 | 0.169 | 0.347 | 0.000 | 0.000 | 0.000 | 0.000 | 0.000 | 0.000 | 0.000 | 0.000 | 0.000 | -1.0203 | 0.3098 | 1.0000 |
| *hsa-miR-2682-5p* | chr1:98510863-98510885 | 0.339 | 0.174 | 0.000 | 0.000 | 0.000 | 0.000 | 0.000 | 0.000 | 0.000 | 0.000 | 0.000 | -1.0195 | 0.3111 | 1.0000 |
| *hsa-miR-26a-5p* | chr3:38010904-38010925; | 218090.003 | 147774.785 | 230351.456 | 118812.393 | 69330.889 | 92383.624 | 106957.296 | 154204.991 | 81683.526 | 79748.856 | 55892.251 | -1.0153 | 0.0050 | 0.5030 |
|  | chr12:58218441-58218462 |  |  |  |  |  |  |  |  |  |  |  |  |  |  |
| *hsa-miR-4788* | chr3:134156678-134156699 | 0.847 | 0.521 | 0.000 | 0.534 | 0.000 | 0.317 | 0.000 | 0.416 | 0.499 | 0.000 | 0.000 | -1.0139 | 0.4002 | 1.0000 |
